# Oxygen-releasing scaffolds in tissue engineering: design strategies, fabrication and regenerative applications

**DOI:** 10.1093/rb/rbag096

**Published:** 2026-05-13

**Authors:** Yuqing Shang, Lele Wang, Huoyun Shen, Mingzhu Jia, Hongxia Gao, Yaqiong Liu, Yumin Yang, Jian Yang, Linliang Wu, Guicai Li

**Affiliations:** Jiangsu Key Laboratory of Tissue Engineering and Neuroregeneration, Key Laboratory of Neuroregeneration of Ministry of Education, Co-Innovation Center of Neuroregeneration, Nantong University, Nantong 226001, China; Jiangsu Key Laboratory of Tissue Engineering and Neuroregeneration, Key Laboratory of Neuroregeneration of Ministry of Education, Co-Innovation Center of Neuroregeneration, Nantong University, Nantong 226001, China; Jiangsu Key Laboratory of Tissue Engineering and Neuroregeneration, Key Laboratory of Neuroregeneration of Ministry of Education, Co-Innovation Center of Neuroregeneration, Nantong University, Nantong 226001, China; Jiangsu Key Laboratory of Tissue Engineering and Neuroregeneration, Key Laboratory of Neuroregeneration of Ministry of Education, Co-Innovation Center of Neuroregeneration, Nantong University, Nantong 226001, China; Jiangsu Key Laboratory of Tissue Engineering and Neuroregeneration, Key Laboratory of Neuroregeneration of Ministry of Education, Co-Innovation Center of Neuroregeneration, Nantong University, Nantong 226001, China; Jiangsu Key Laboratory of Tissue Engineering and Neuroregeneration, Key Laboratory of Neuroregeneration of Ministry of Education, Co-Innovation Center of Neuroregeneration, Nantong University, Nantong 226001, China; Jiangsu Key Laboratory of Tissue Engineering and Neuroregeneration, Key Laboratory of Neuroregeneration of Ministry of Education, Co-Innovation Center of Neuroregeneration, Nantong University, Nantong 226001, China; Department of Neurosurgery, People’s Hospital of Deyang City, Sichuan Clinical Research Center for Neurological Diseases, Deyang 618000, China; Affiliated Rugao Hospital of Xinglin College, The People’s Hospital of Rugao, Co-innovation Center of Neuroregeneration, Nantong University, Nantong 226500, China; Jiangsu Key Laboratory of Tissue Engineering and Neuroregeneration, Key Laboratory of Neuroregeneration of Ministry of Education, Co-Innovation Center of Neuroregeneration, Nantong University, Nantong 226001, China

**Keywords:** tissue engineering, hypoxia, oxygen-releasing materials, oxygen-releasing scaffolds, tissue regeneration

## Abstract

Oxygen is indispensable for sustaining tissue and cell physiological functions, yet a hypoxic microenvironment often arises in tissue injury and regeneration, impairing graft scaffold performance and hindering repair processes. Thus, oxygen-releasing scaffolds have become a pivotal research frontier in tissue engineering. Via elaborate spatial constructions (e.g. electrospun fibers, hydrogels, microspheres) and diverse modification strategies, these scaffolds enable sustained, spatiotemporally controllable oxygen release at injury sites, alleviating local hypoxia and boosting tissue regeneration. They show great application potential in repairing skin, bone, nerve and cardiovascular tissues, as well as in tumor therapy, though comprehensive systematic summaries of their tissue engineering applications remain scarce. This review aims to delineate domain advances systematically: it first gives a holistic overview of core aspects, including oxygen carrier categories and delivery mechanisms, scaffold material modification and functionalization, intelligent responsiveness optimization and reactive oxygen species scavenging paradigms, then comprehensively recapitulates their application status and therapeutic efficacy across diverse scenarios (skin wound repair, bone defect regeneration, nerve injury remediation, tumor therapy, cardiovascular tissue reconstruction); finally, dissects practical application challenges and proposes future development trends, expecting to provide valuable references for in-depth research and clinical translation of oxygen-releasing materials in tissue repair and reconstruction.

## Graphical Abstract

**Graphical Abstract:**
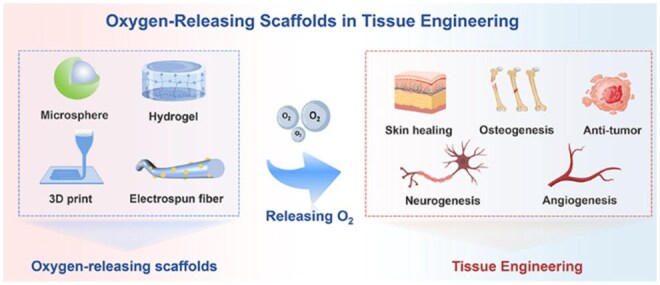

## Introduction

Oxygen is an essential substance for maintaining normal cellular physiological functions and metabolic activities. It plays a crucial regulatory role in regenerative processes such as inflammation resolution, vascular reconstruction and functional recovery following tissue injury [1, 2]. After tissue injury, the deep part of the damaged tissue is commonly accompanied by vascular network disruption, leading to the formation of a persistent hypoxic microenvironment. This hypoxic state reduces cellular activity in the damaged tissue, hinders the effective release of vascular endothelial growth factor (VEGF) and the timely formation of new blood vessels, thereby delaying the repair process [2, 3]. The mechanisms by which oxygen affects different tissues and cells vary slightly. In skin tissue, vascular network disruption caused by burns or other wound traumas leads to reduced local oxygen supply, affecting the activity of fibroblasts at the wound site and consequently slowing the healing rate [4, 5]. Bone tissue is highly vascularized. Studies have shown that treating osteonecrosis with hyperbaric oxygen therapy, which increases the partial pressure of oxygen in the blood to provide a sustained oxygen supply to hypoxic sites, can promote VEGF release, stimulate neovascularization and improve microcirculation [6, 7]. At the same time, it enhances leukocyte phagocytosis, providing a more stable repair environment for bone tissue by clearing pathogens and metabolic waste [8, 9]. Neural tissue is particularly sensitive to hypoxia. Following a brief period of hypoxia after injury, neurons can often undergo self-protection and repair through adaptive mechanisms. For example, under hypoxic conditions, hypoxia-inducible factor (HIF-1α) is activated, promoting VEGF release and facilitating vascularization. However, this is insufficient to induce the formation of new blood vessels. Since vascularization is a prerequisite for neural regeneration, providing oxygen supply for vascular reconstruction after nerve injury is particularly important [10, 11].

Tissue engineering scaffolds encompass three factors: scaffolds, growth factors and cells. Currently, they exist in various forms, including hydrogel scaffolds [12, 13], electrospun fiber scaffolds [14–16], freeze-dried porous sponge scaffolds [17], microspheres [18, 19] and 3D-printed scaffolds [20–22] and are widely used in the regeneration of skin, bone, nerve, cartilage and other tissues [4, 23–26]. However, the actual therapeutic efficacy of a scaffold depends not only on material selection and structural design but also on its active regulation of the regenerative microenvironment surrounding the damaged tissue [27, 28]. Among these factors, the survival of transplanted cells within the scaffold and host blood vessel ingrowth are highly dependent on local oxygen supply levels. Before new blood vessels form, oxygen delivery plays a critical role in cell survival throughout the tissue [29–31], while the hypoxic microenvironment deep within the tissue often limits the therapeutic efficacy of the scaffold [32]. Therefore, achieving sustained and controllable oxygen supply at the injury site has become one of the key challenges that tissue engineering urgently needs to address.

**Figure 1:**
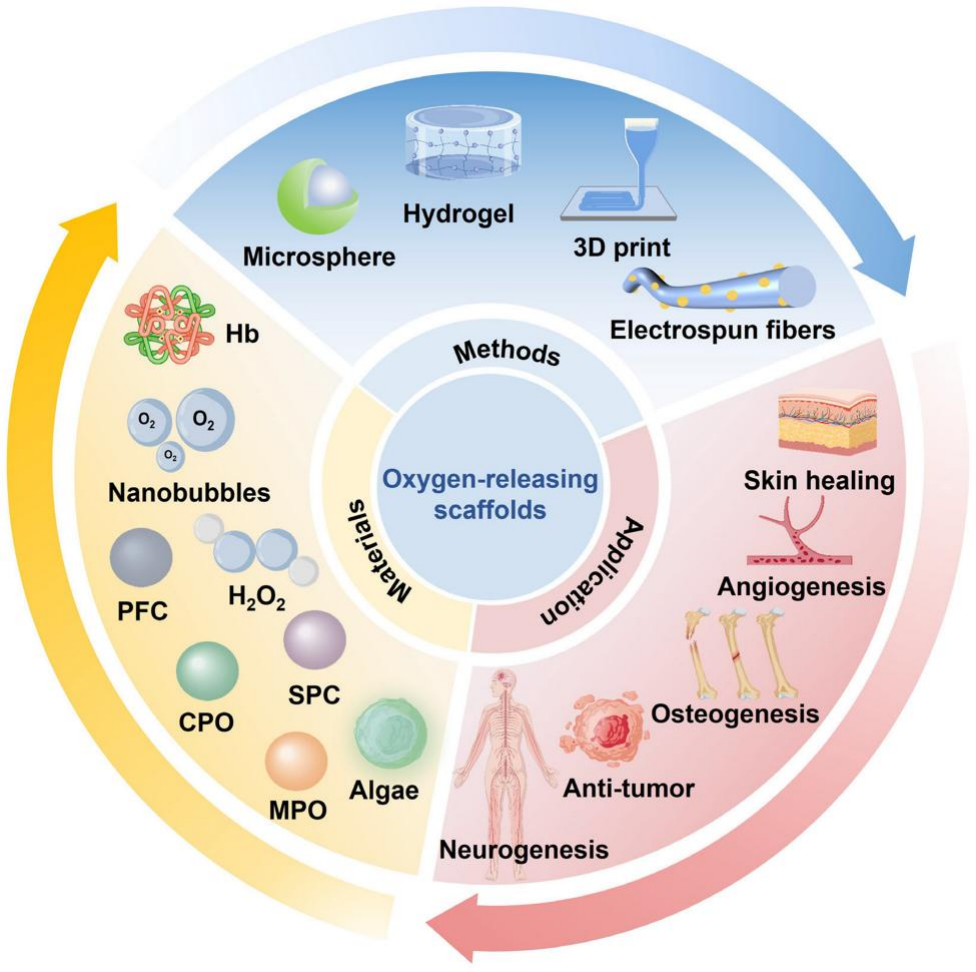
Schematic diagram illustrating an overview of oxygen-releasing materials, methods for preparing oxygen-releasing scaffolds and their applications in various tissue engineering fields. Perfluorocarbons (PFC), hemoglobin (Hb), sodium percarbonate (SPC), calcium peroxide (CPO) and magnesium peroxide (MPO) are in this figure. Created with BioGDP.com [33].

Oxygen-releasing scaffolds, as a type of biomaterial scaffold capable of sustained or responsive oxygen release, represent a feasible strategy to address the aforementioned hypoxic microenvironment challenges. This article systematically summarizes recent research progress on oxygen-releasing scaffolds in the fields of tissue engineering and regenerative medicine. It introduces the characteristics of tissue regeneration for various tissues, commonly used oxygen-releasing materials and scaffold preparation strategies and reviews the applications of oxygen-releasing scaffolds in skin, bone, nerve, cardiovascular and tumor therapy (as shown in Figure 1). Finally, the future development and clinical translation of oxygen-releasing scaffolds are discussed, with the hope of providing research ideas and strategies for the subsequent design and related applications of tissue engineering oxygen-releasing scaffolds.

## Tissue regeneration

Tissues possess a certain regenerative capacity. After injury, damaged tissues and cells can self-repair their structure and function through complex biological processes. The regenerative capacity varies among different tissue types due to differences in their structure and cellular composition. This process often relies on the regulation by tissue cells, signaling pathways and the microenvironment [34–37].

### Skin tissue regeneration

**Figure 2:**
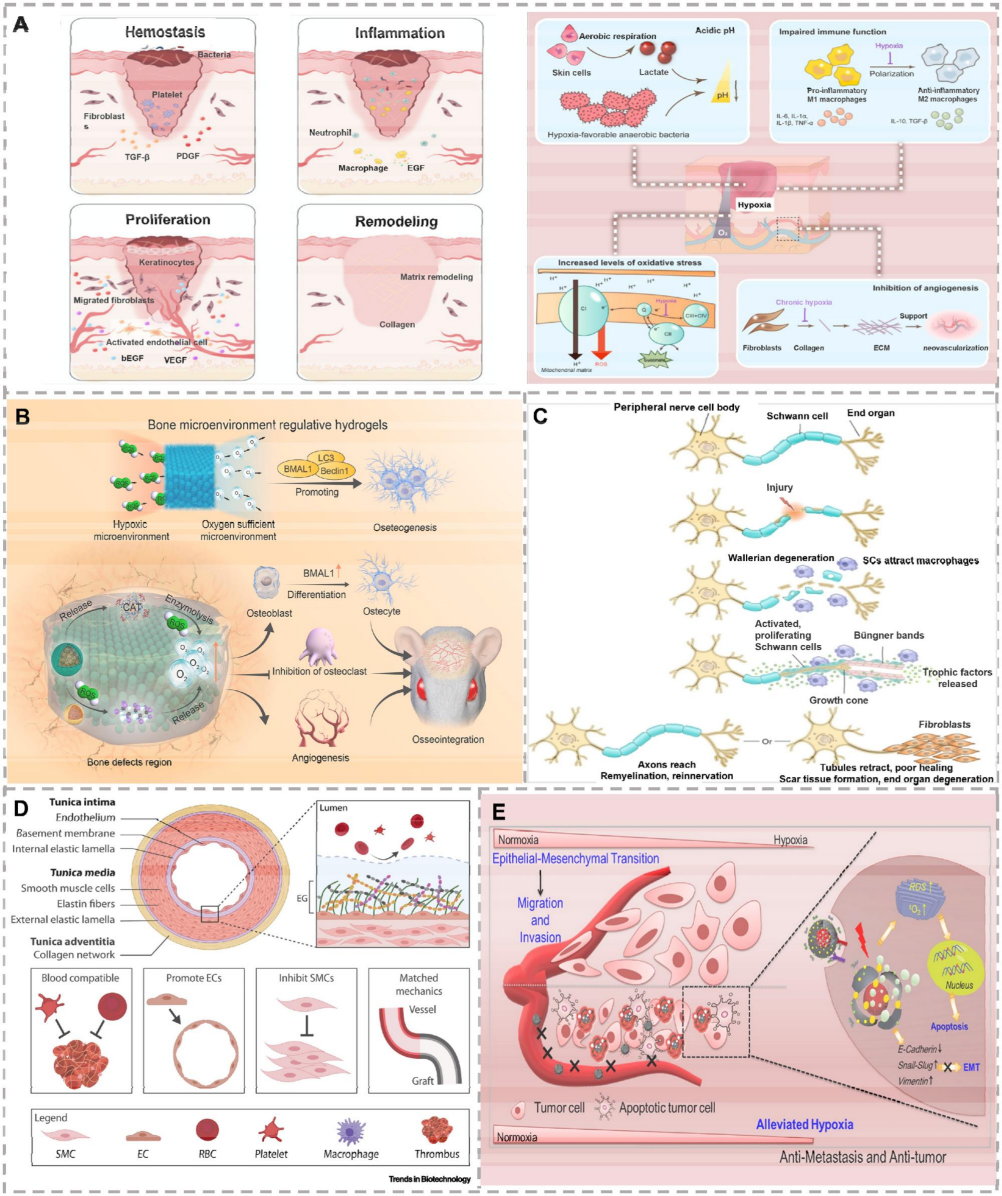
Introduction to various tissue regeneration processes. (**A**) Skin wound healing process (hemostasis, inflammation, proliferation, remodeling) and changes after skin wound hypoxia [40]. Copyright 2023, ACS Publications. (**B**) Oxygen-releasing scaffold promoting bone tissue regeneration [41]. Copyright 2023, Elsevier. (**C**) Characteristics of neural tissue regeneration [42]. Copyright 2022, American Physiological Society. (**D**) General structure of a blood vessel artery and design criteria for vascular grafts [43]. Copyright 2021, Elsevier. (**E**) Changes in the tumor microenvironment under normoxic and hypoxic conditions [44]. Copyright 2022, Springer.

The skin serves as the body’s first line of defense, consisting of the epidermis and dermis. Its structural integrity is crucial for resisting external pathogen invasion and maintaining internal homeostasis [38, 39]. Skin wound healing is divided into four stages, as shown in Figure 2A, hemostasis, inflammation, proliferation and remodeling [45]. During the hemostasis phase, platelets aggregate and release factors such as TGF-β and PDGF, initiating the coagulation process. Upon entering the inflammatory phase, vasoconstriction and increased cellular oxygen consumption cause local hypoxia. Neutrophils and macrophages sequentially infiltrate the wound to clear pathogens and necrotic tissue, during which a large amount of molecular oxygen is consumed and converted into reactive oxygen species (ROS). In the proliferation phase, keratinocytes migrate to the wound edge. During this stage, hypoxia enhances the motility of these cells by inducing urokinase plasminogen activator expression and mTORC1 signaling. However, the proliferation and maturation of keratinocytes require sufficient oxygen for ATP production. Due to insufficient oxygen, HIF-1α is activated during this phase, releasing VEGF to promote endothelial cells growth and subsequent neovascularization. In the remodeling phase, collagen fibers are reorganized to achieve structural stabilization of the wound. Both the proliferation and remodeling phases have high oxygen demands. Slight local hypoxia initiates HIF-1α activation as part of the early healing process, but persistent hypoxia may lead to the formation of chronic wounds [46]. Small wounds can heal spontaneously through the skin’s regenerative capacity without intervention, whereas larger wounds heal more slowly and are more susceptible to bacterial infection during the healing process. Moreover, large-area or deep trauma causes local blood flow interruption due to vascular network destruction, creating a sustained hypoxic microenvironment. In addition, conventional wound dressings often further hinder the diffusion of oxygen from the external environment into the wound, exacerbating local hypoxia, thereby inhibiting fibroblast activity, delaying neovascularization and increasing infection risk [47, 48]. Furthermore, the inability of chronic wounds to adapt to hypoxia exacerbates tissue damage. Excessive upregulation of HIF-1α can impede normal wound healing. Hypoxia also leads to increased oxidative stress, causing lactate accumulation and an acidic environment that favors anaerobic bacterial colonization. At the same time, hypoxia drives macrophages toward the pro-inflammatory M1 phenotype and inhibits their transition to the anti-inflammatory M2 phenotype, resulting in impaired immune function. Additionally, in a hypoxic environment, fibroblast activity is suppressed, reducing collagen synthesis and matrix deposition. Meanwhile, the expression and function of key angiogenic factors such as VEGF are impaired, ultimately leading to hindered neovascularization, poor granulation tissue formation and an overall delay in the wound healing process [49]. Therefore, skin tissue engineering scaffolds that integrate both antibacterial and oxygen-supplying functions are of great significance for alleviating wound hypoxia and accelerating healing.

### Bone tissue regeneration

Bone tissue plays a vital role in the human body. As the attachment point for muscles, it enables movement through interactions between bone cells and nerve fibers [21, 50]. In addition, bone marrow is the primary hematopoietic organ, playing an important role in maintaining blood circulation and immune function [51–53]. External trauma, surgical accidents and skeletal abnormalities can cause bone tissue defects. After injury, bone tissue possesses a certain regenerative capacity. Bone cells can self-repair small bone defects (<6 mm) through the synergistic actions of osteoblasts, osteoclasts and chondrocytes [54]. Bone injury is accompanied by vascular disruption, and the oxygen partial pressure within the hematoma can drop to as low as 1% O_2_ [55]. This early hypoxic environment stabilizes HIF-1α, inducing the expression of key osteogenic genes such as BMP2 and activating the osteogenic differentiation program of mesenchymal stem cells [56]. Subsequently, HIF-1α continuously drives the expression of angiogenic factors such as VEGF, promoting the ingrowth of new blood vessels into the defect area and providing the oxygen supply foundation for the subsequent conversion of soft callus to hard callus. During the mineralization and maturation phase, the active-matrix synthesis and calcium deposition of osteoblasts become highly dependent on adequate oxygen supply. However, while moderate hypoxia promotes vascularization and osteogenic differentiation, severe and chronic hypoxia reduces mineral deposition and impairs osteogenesis [57]. Severe bone injury is difficult to repair, as the damage process is often accompanied by the interruption of local blood supply, leading to ischemia and hypoxia. This in turn inhibits osteoblast activity and impedes the ingrowth of new blood vessels, necessitating surgical intervention [58, 59]. Clinically, treatment strategies for bone defects typically include autologous bone grafting, allogeneic bone grafting or bone graft substitutes [60]. The most promising artificial bone graft substitutes can achieve controlled drug release by incorporating intelligent responsive biomaterials for remote electrical stimulation [61], magnetic stimulation [62] or ultrasonic stimulation [63] of the scaffold. Material design and structural customization can provide good mechanical support and osteoconductivity. However, the persistent existence of the hypoxic microenvironment still severely restricts their *in vivo* osteogenic efficacy. In recent years, oxygen-releasing biomaterials have become an important strategy to address this challenge. Oxygen tension regulates cell behavior through metabolic programming, thereby affecting tissue regeneration. Biomaterials with oxygen-releasing capabilities can enhance therapeutic outcomes and reduce hypoxia-induced tissue damage [2]. Bone tissue is highly vascularized, and adequate diffusion and sustained supply of oxygen during its regeneration process are essential, serving as prerequisites for vascular reconstruction and osteogenic differentiation. Incorporating oxygen carriers into bone regeneration scaffolds to provide sustained oxygen supply has been proven to effectively promote early vascular reconstruction and osteogenic differentiation. As shown in Figure 2B, Sun *et al*. [41] prepared an oxygen-releasing hydrogel scaffold that can reverse the hypoxic microenvironment in bone defect areas, providing adequate oxygen for the bone defect, thereby promoting angiogenesis and osteogenesis, indicating the application potential of oxygen-releasing scaffolds in bone tissue repair.

### Neural tissue regeneration

The peripheral nervous system includes all nerves outside the brain and spinal cord, originating from the central nervous system. Its repair following injury involves processes such as axonal transection, myelin degeneration, Wallerian degeneration and macrophage-mediated debris clearance. Subsequently, through the synergistic actions of Schwann cell proliferation and neurotrophic factors, axonal regeneration and Büngner band formation are initiated (Figure 2C) [42]. However, the efficiency of postinjury repair depends on the local oxygenation state. In the early stage after injury, acute damage causes vascular rupture, leading to local hypoxia. Under mild hypoxia, macrophages selectively sense hypoxic signals within the bridging region, activating the HIF-1α pathway. By secreting VEGF-A, they promote vascular network formation to guide Schwann cell migration and drive the formation of new vascular networks, providing structural guidance and oxygen supply support for axonal regeneration. However, axonal extension itself is a highly energy-demanding process that relies heavily on ATP provided by mitochondrial aerobic respiration, making it extremely sensitive to hypoxia. During the myelination stage, the synthesis and wrapping of myelin by Schwann cells is also a highly oxygen-consuming process. However, endogenous hypoxia and angiogenic signals are often insufficient to support complete repair [64]. Moreover, severe or sustained hypoxia can activate p53-dependent apoptosis, which not only hinders the expression of key factors such as VEGF and delays neovascularization, but also directly inhibits Schwann cell activity and axonal extension [1]. Current tissue-engineered nerve guidance conduits (NGCs) serve as an alternative to autologous nerve grafting. Most related studies load Schwann cells into NGCs to mimic the regenerative microenvironment and enhance peripheral nerve regeneration [65, 66]. However, during the early stage of implantation, oxygen diffusion within the NGCs is limited, while the establishment of new blood vessels typically takes 1–2 weeks. The combination of these factors leads to a sustained hypoxic microenvironment inside the implant, significantly reducing the survival rate of seeded cells and severely compromising its *in vivo* therapeutic efficacy [67, 68]. Therefore, providing timely and sustained exogenous oxygen support during the early phase of neural regeneration and constructing NGCs with time-sequenced oxygen supply capabilities represent key strategies for enhancing the therapeutic efficacy of NGCs.

### Cardiovascular tissue regeneration

The critical functions of the heart largely depend on a continuous oxygen supply. When this supply is reduced, myocardial hypoxia can lead to ischemic injury and impaired cardiac function [25, 69]. The oxygen diffusion radius of natural capillaries is only 100–200 μm, whereas the ischemic microenvironment following myocardial infarction can trigger irreversible cardiomyocyte necrosis within 6 min. The hypoxic microenvironment, thus, becomes a key bottleneck for cardiovascular regeneration and repair [70]. The formation of blood vessels is a crucial prerequisite for tissue regeneration in the organism. Under normal physiological conditions, tissues rely on the vascular circulatory system to provide cells with essential nutrients and oxygen for survival, aiding cellular respiration. As shown in Figure 2D, the three-layer structure of the blood vessel wall is clearly illustrated: the intima formed by endothelial cells, the middle layer composed of smooth muscle cells and elastic fibers and the adventitia consisting of collagen fibers. The process of new blood vessel formation includes vasculogenesis, angiogenesis and arteriogenesis [71]. Vasculogenesis refers to the process where progenitor cells migrate to sites of vessel formation and differentiate into vascular endothelial cells, which mainly occurs during embryonic development [72]. Angiogenesis refers to the sprouting of new capillary branches (with diameters <15 μm) from pre-existing blood vessels in the organism [73]. Arteriogenesis refers to the remodeling of existing arteries in the body, increasing their luminal diameter in response to increased blood flow. Among these, angiogenesis occurs during embryonic development, wound healing and pathological processes. Its main process is as follows: after vascular injury, coagulation and inflammation phases occur first. Subsequently, growth factors such as VEGF promote the migration and proliferation of vascular endothelial cells. Vascular smooth muscle cells then proliferate and migrate, participating in the remodeling of the vascular wall and promoting the maturation and stabilization of the regenerated vessel. In tissue engineering, constructing bioactive vascular scaffolds is key to promoting tissue regeneration. Their design must meet multiple requirements: excellent biocompatibility and degradability, a three-dimensional porous structure conducive to cell growth and material transport, mechanical properties matching native tissues and the ability to promote vascular endothelial cell adhesion and growth while inhibiting smooth muscle cell overproliferation through surface functionalization. However, the actual therapeutic efficacy of a scaffold depends not only on the aforementioned design elements, but also on the oxygenation status of the local microenvironment at the injury site. In the early phase following vascular injury, the disruption of blood supply leads to local tissue hypoxia, which activates the HIF-1α signaling pathway. Under hypoxic conditions, HIF-1α accumulates, dimerizes with HIF-β, recruits co-activators CBP/P300 and activates the gene transcription of angiogenic cytokines such as VEGF. These factors bind to receptors on endothelial cells, initiating the signaling transduction pathways for endothelial cell sprouting and angiogenesis [74]. Notably, the oxygen demand varies across different stages of vascular regeneration. Early hypoxia is a key signal for activating the HIF-1α pathway and initiating vascular sprouting. During the intermediate stage, the hypoxic environment activates factors such as VEGF to promote endothelial cell proliferation and migration. At this stage, HIF-1α drives glycolytic reprogramming by upregulating PDK1 and LDHA, providing energy and synthetic precursors for endothelial cell proliferation. In the later stage, an adequate oxygen supply is required to promote the proper remodeling of the vascular wall [75]. As local oxygen supply is restored, the activity of PHDs increases, leading to the hydroxylation of HIF-1α and its subsequent degradation via the VHL-mediated ubiquitin-proteasome pathway, accompanied by a decrease in VEGF levels. Following HIF-1α degradation, platelet-derived growth factor-BB (PDGF-BB) and angiopoietin-1 secreted by endothelial cells begin to play dominant roles. They recruit pericytes and smooth muscle cells to encircle the lumen of new blood vessels, promoting basement membrane deposition and the formation of intercellular tight junctions, ultimately achieving functional maturation of the vessels. If hypoxia persists and is not resolved, the sustained activation of HIF-1α leads to chronically elevated VEGF expression. New blood vessels remain in an immature state, which not only fails to effectively deliver oxygen but also exacerbates tissue edema and inflammatory responses due to increased vascular permeability. However, the VEGF secreted in response to endogenous hypoxia is often insufficient to fully support the entire process of angiogenesis, and prolonged hypoxia exacerbates inflammatory responses, causing secondary damage to surrounding tissues. Therefore, incorporating exogenous oxygen carriers into the design of cardiovascular tissue engineering scaffolds and precisely matching the stage-specific oxygen demands of vascular regeneration through time-sequenced oxygen delivery is of decisive significance for achieving successful cardiovascular tissue regeneration. Specifically, this involves maintaining moderate hypoxia in the early implantation stage to preserve sprouting signals and restoring adequate oxygen supply in the later stage to promote vascular maturation [76].

### Tumor tissue

Cancer is a global public health issue. It is often induced by various causes and can originate from any organ or structure in the body [77]. Tumors are characterized by abnormal cell growth. When the number of newly formed cancer cells is small, the body’s immune cells have difficulty recognizing and destroying them. Tumors can typically only be detected when they reach a size of approximately 1 cm [78]. Clinically, there is currently no definitive cure for cancer. For early-stage tumors without metastasis, surgical resection can be employed. Additionally, interventions such as radiotherapy, chemotherapy and immunotherapy can be used to inhibit cancer cell growth [79]. However, in general, tumor cells are highly invasive with indistinct boundaries, making complete tumor removal by surgery alone difficult and highly prone to tumor recurrence. Therefore, postoperative radiotherapy and chemotherapy are particularly important. Hypoxia is one of the hallmark features of the solid tumor microenvironment, with approximately 90% of solid tumors containing hypoxic regions to varying degrees [80]. Figure 2E illustrates that cancer cells in a hypoxic environment are more prone to spontaneous metastasis. The hypoxic microenvironment drives tumor cells to undergo epithelial-mesenchymal transition, significantly enhancing their migration and invasion capabilities. At the same time, hypoxia promotes a metabolic shift toward glycolysis for energy production in tumor cells, generating lactate that acidifies the microenvironment. Moreover, hypoxia impairs DNA repair mechanisms and upregulates the expression of drug resistance-related proteins, which contributes to the poor efficacy of radiotherapy and chemotherapy. The increased propensity for spontaneous metastasis of cancer cells under hypoxic conditions is a key factor leading to local tumor recurrence and distant metastasis in cancer patients following surgery or radiotherapy. Alleviating hypoxia can inhibit this process, induce cell apoptosis and exert antitumor and anti-metastatic effects [81, 82]. Furthermore, hypoxia can lead to drug resistance at the tumor site, which is detrimental to cancer treatment. For example, temozolomide is a drug effective in reducing tumor cell invasion, but its efficacy is related to the overexpression of O6-methylguanine-DNA-methyltransferase (MGMT) in tumors. MGMT expression is influenced by the oxygen content in the tumor microenvironment. Hypoxia upregulates MGMT expression, hindering the effectiveness of temozolomide treatment. Additionally, studies have shown that in cancer treatment, combining radiotherapy with oxygen supply can effectively enhance tumor cell sensitivity to radiation [83]. The mechanism involves oxygen promoting excessive active oxygen (ROS) production and inhibiting the self-repair of DNA damage, playing a key role in sensitizing radiation-induced cancer cell killing [84]. Photodynamic therapy (PDT), due to its advantages such as minimal invasiveness and low systemic toxicity, has become an important cancer treatment modality. The principle of PDT is that a photosensitizer absorbs light energy to stimulate the generation of toxic ROS, thereby killing tumor cells. However, most current PDT relies on an O_2_-dependent Type II photoreaction mechanism: energy is transferred from the excited photosensitizer to O_2_ molecules, consuming O_2_ to produce singlet oxygen (^1^O_2_), thereby killing tumor cells. However, the hypoxic microenvironment of solid tumors severely limits the efficacy of PDT, and the consumption of O_2_ during treatment further exacerbates hypoxia, promoting tumor growth and metastasis. Therefore, fabricating scaffolds capable of providing oxygen and combining them with PDT offers a promising approach to overcoming the therapeutic bottleneck in tumor treatment.

## Oxygen-releasing materials

The regeneration processes of various tissues all require an oxygen supply. Oxygen-releasing materials have evolved into a major research focus in the biomedical field in recent years. Their primary principle is to provide sufficient oxygen during tissue repair through physical or chemical means. Based on their different oxygen-release mechanisms, oxygen-releasing materials can be categorized as follows.

### Oxygen-carrier materials

This type of material stores oxygen through physical adsorption or chemical binding and releases it under specific conditions. For example, biological macromolecules such as hemoglobin (Hb) and perfluorocarbons (PFCs) possess good oxygen-carrying capacity and can serve as oxygen carrier materials. Table 1 lists the oxygen release mechanisms and advantages/disadvantages of various oxygen-releasing materials.

#### Perfluorocarbons

**Figure 3:**
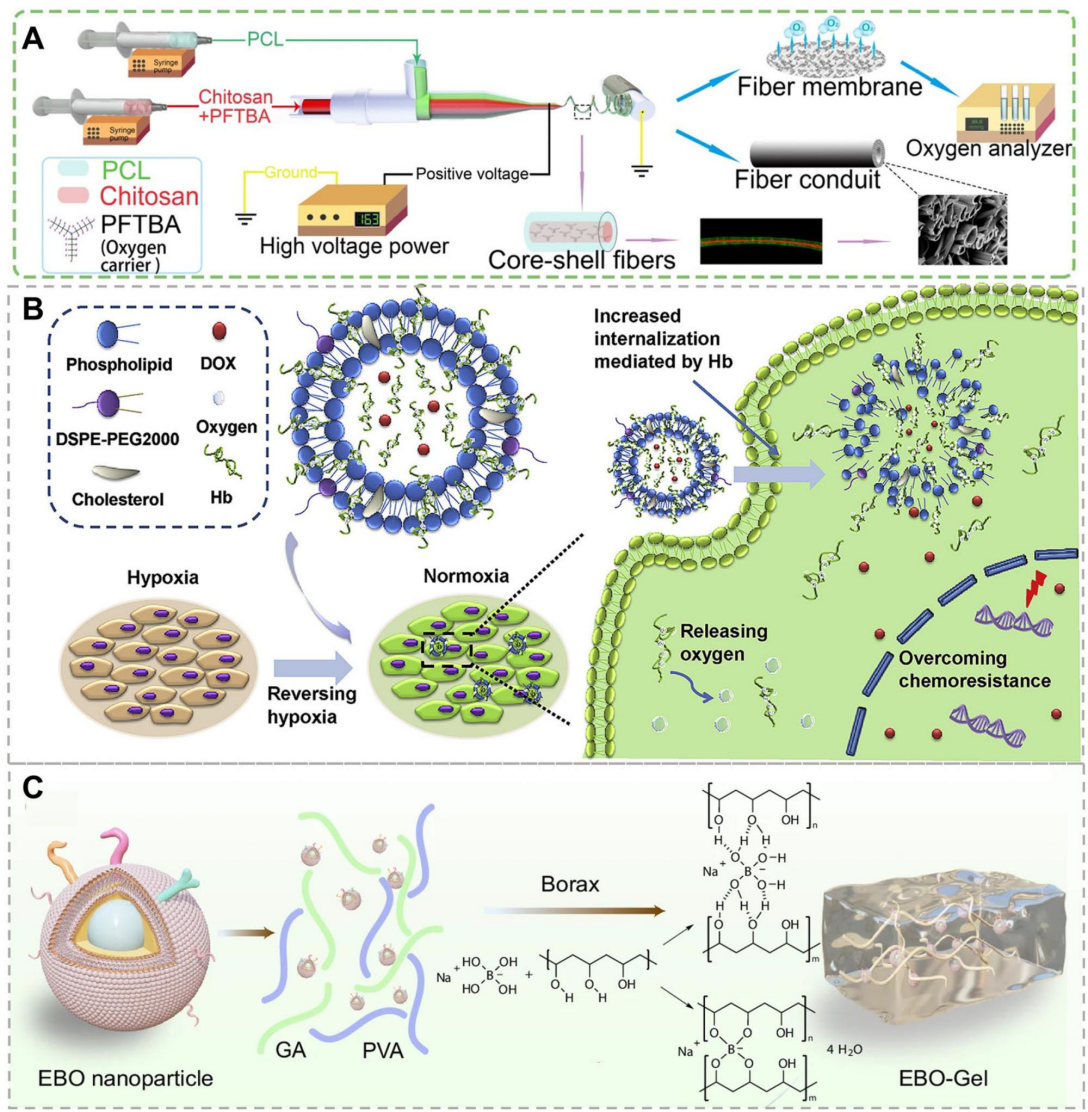
Research progress in oxygen-carrying materials. (**A**) Diagram of the preparation of coaxial electrospun fibers loaded with PFTBA [88]. Copyright 2025, Ivyspring International Publisher. (**B**) Schematic diagram illustrating liposome-based Hb-mediated endocytosis, oxygen level enhancement and reversal of hypoxia-induced chemoresistance [89]. Copyright 2018, Elsevier. (**C**) Diagram illustrating the synthesis of an exosomal nanovesicle-embedded hydrogel designed for oxygen transport [90]. Copyright 2024, Springer Nature.

PFCs are a class of organic compounds with high chemical stability. Owing to the weak van der Waals interactions among PFC molecules, liquid PFCs exhibit high oxygen solubility, enabling them to carry a substantial amount of oxygen [85]. They transport oxygen to hypoxic tissues via passive diffusion and possess the ability to permeate red blood cells [83]. Currently, PFCs are clinically used in applications, for instance, ultrasound (US) imaging and fluorine magnetic resonance imaging, artificial blood substitutes, and organ preservation [86]. PFCs include various compounds such as perfluorotributylamine (PFTBA), pentafluorophenyl, perfluorohexane and perfluorooctyl bromide. Among these, PFTBA, as an ideal carrier for oxygen delivery [68], has been implemented within the field of tissue engineering. Ma *et al*. [87] incorporated PFTBA into fibrin hydrogel and injected it into a collagen-chitosan conduit seeded with Schwann cells. The study found that this oxygen-carrying scaffold could support Schwann cell survival for 28 days, which was significantly longer than scaffolds without PFTBA. Furthermore, Ma *et al*. [88] also adopted a coaxial electrospinning approach to encapsulate PFTBA, forming a core-shell structure. The preparation process is illustrated in Figure 3A. The results demonstrated that this approach could significantly promote the recovery of sciatic nerve injuries in rats. Relevant studies also exist in bone tissue engineering. To obtain microparticles capable of sustained oxygen release, Kim *et al*. [91] prepared hollow microparticles loaded with PFC emulsion using a water-in-oil-in-water (W/O/W) emulsion solvent evaporation technique. These were used as oxygen carriers for mandible regeneration in pigs. The results demonstrated that the oxygen-releasing particles significantly enhanced cell viability under hypoxic conditions, and *in vivo* experiments demonstrated faster enhanced osteogenesis and increased bone mineral density. This highlights the application potential of PFCs in the field of tissue engineering. However, although studies indicate PFCs are nontoxic, their long-term toxicity and safety after implantation have not been sufficiently verified. PFCs have not been granted FDA approval for clinical administration. Moreover, the high purification cost of PFCs is also a factor limiting their application [92].

#### Hemoglobin

Hb is defined as a red blood cell protein whose principal role is to bind and transport oxygen in the bloodstream. Hb is composed of four subunits (α1, β1, α2 and β2), each containing a heme prosthetic group. The iron ion within the heme binds with oxygen to form oxyhemoglobin, thereby transporting oxygen from the lungs to various organs and tissues throughout the human body [93, 94]. As a natural protein material, hemoglobin exhibits good biocompatibility with the human physiological environment and possesses high oxygen affinity. Reversible binding of oxygen to Hb can be achieved by modulating factors such as temperature and pH [95–98]. However, when implanted as an oxygen carrier, Hb exhibits poor stability, rapidly dissociating into dimers and monomers, which possess nephrotoxicity and oxidative toxicity [99]. Chemical modifications are often necessary to enhance its safety [98]. Researchers have continuously improved the crosslinking and preparation processes for Hb, achieving certain progress, including modification methods such as glutaraldehyde crosslinking and liposome encapsulation in recent years [70]. For example, Hu *et al*. [100] combined co-precipitation of Hb with an inorganic carbonate template and the spontaneous adhesion of Polydopamine (PDA) to reduce Hb dissociation. The results showed that this modification method enhanced Hb stability, reduced Hb dissociation and the toxicity of free Hb monomers, and decreased oxidative damage to human umbilical vein endothelial cells (HUVECs), demonstrating application potential. As shown in Figure 3B, Yang *et al*. [89] combined Hb with the anticancer drug doxorubicin (DOX) to prepare composite liposomes, achieving oxygen delivery to the tumor site. This led to increased drug accumulation at the tumor site, enhanced endocytosis by cancer cells and elevated oxygen levels in the tumor region, demonstrating stronger antitumor effects. In summary, although Hb exhibits poor stability after implantation, its structural stability can be effectively enhanced through mature modification strategies such as polymer conjugation or microencapsulation. Benefiting from its inherent biocompatibility as a natural protein and ease of functional modification, hemoglobin-based oxygen carriers demonstrate significant advantages in tissue engineering, showing clear potential, particularly in alleviating hypoxia in transplantation areas and supporting cell survival and function. Therefore, with rational engineering design, hemoglobin remains a highly promising oxygen carrier material, expected to provide effective oxygenation support for tissue regeneration.

#### Oxygen-carrying nanobubbles

Nanobubbles are a novel type of micro/nano-scale structure that has received significant attention in recent years. Composed of a gas core and a stable shell, they possess a series of unique physicochemical properties, granting them significant application potential in the domain of regenerative medicine. Nanobubbles (with particle sizes around 100 nm) exhibit an extraordinarily high surface area relative to their volume, slow rising velocity and a significant self-pressurization effect. These properties collectively ensure their long-term stability in liquid environments and enable the controlled, sustained release of gases [101]. Although nanobubbles were relatively limited in early tissue engineering research, primarily constrained by their complex preparation processes and the uncertainty of the *in vivo* environment, their slow and sustained gas release characteristics confer unique advantages in regenerative tissues requiring long-term oxygen supply, such as nerve regeneration, cartilage repair and diabetic wounds [102, 103]. Studies have shown that by adjusting the size and surface chemistry of oxygen-loaded nanobubbles, they can undergo deformation or rupture triggered by specific biological signals such as US, pH or enzymes. This enables on-demand release of drugs or gases, adapting to different tissue regeneration needs. For example, Ma *et al*. [102] developed an oxygen-carrying microbubble loaded with Mn^2+^, capable of releasing oxygen upon US triggering, enhancing the repair of diabetic wounds. Furthermore, as shown in Figure 3C, Han *et al*. [90] constructed a composite hydrogel system with exosome-coated oxygen nanobubbles, which enhanced the delivery efficacy of exosomes under hypoxia and synergistically improved the wound microenvironment. Although nanobubble technology holds significant potential in regenerative medicine, its clinical application still faces several challenges, including the difficulty of large-scale standardized production, unclear long-term fate and metabolic pathways *in vivo* and potential free radical-related biosafety concerns. Current research is focused on addressing these challenges through methods such as surface modification, integration of smart materials and optimization of release kinetics.

### Self-oxygen-releasing materials

These materials generate oxygen through chemical reactions, such as peroxide decomposition reactions. Materials like hydrogen peroxide (H_2_O_2_) and calcium peroxide (CPO) can decompose under specific conditions to produce oxygen, thereby supplying oxygen to hypoxic tissues.

#### Inorganic peroxides

##### Calcium peroxide

**Figure 4:**
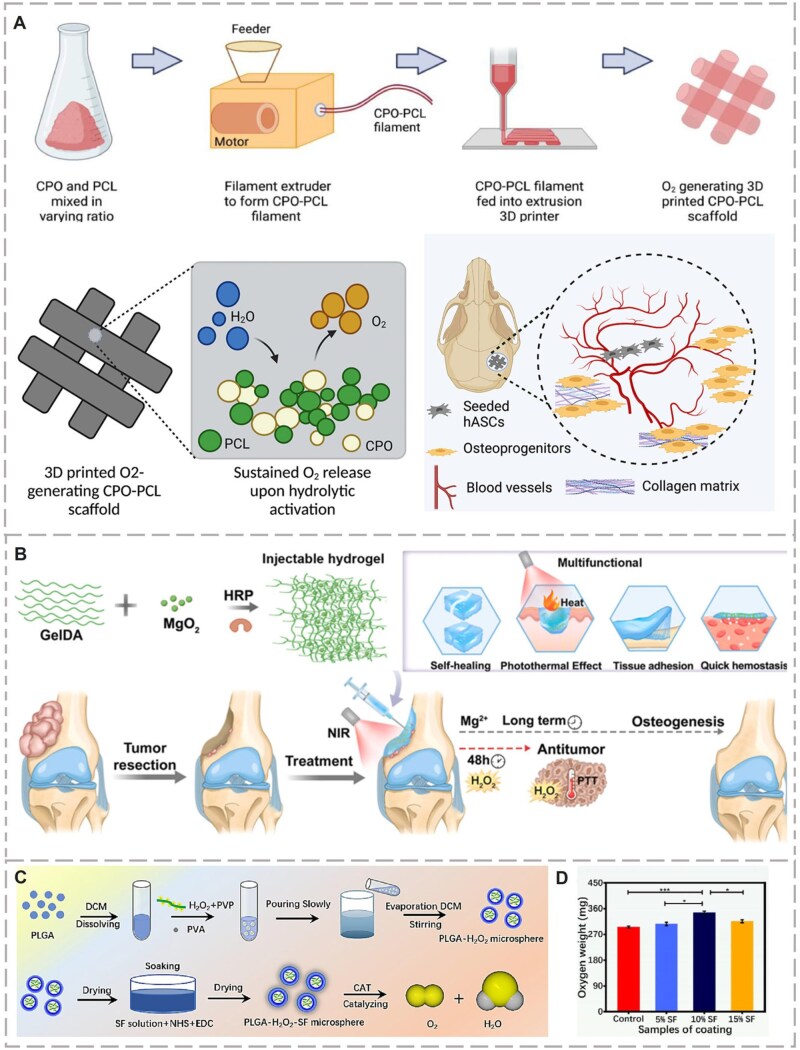
Research progress on inorganic peroxide-based oxygen-releasing materials. (**A**) Schematic illustration of CPO-PCL oxygen-releasing scaffold preparation and its application in cranial defect treatment [29]. Copyright 2024, Elsevier. (**B**) Schematic diagram of MPO gelatin hydrogel for treating bone injury [110]. Copyright 2024, Elsevier. (**C**) Preparation process of core-shell structured PLGA-H_2_O_2_ microspheres, (**D**) Oxygen release amount from H_2_O_2_ microspheres with different SF coating concentrations [111]. Copyright 2023, Elsevier.

CPO is a type of solid peroxide that reacts with water to generate oxygen and calcium ions. It is characterized by low toxicity and strong oxygen production capacity and has been increasingly used as an oxygen carrier in tissue repair and regeneration processes in recent years [4, 104–106]. CPO reacts with water to produce H_2_O_2_ and Ca (OH)_2_. H_2_O_2_ subsequently decomposes into water and oxygen. Due to its strong oxygen production capacity, CPO often requires surface modification with hydrophobic substances such as Polycaprolactone (PCL) [104, 107], polylactic acid (PLA) [108] or PLLA [109] to achieve prolonged and sustained oxygen supply. Because Ca^2+^ is generated during the reaction, CPO is commonly applied in the field of bone regeneration [108]. As shown in Figure 4A, Sarkar *et al*. [29] prepared a 3D-printed hydrogel containing CPO. The study confirmed that even without adding exogenous osteoinductive growth factors, the CPO-PCL group still exhibited a higher volume of new bone formation compared to the PCL group, demonstrating the sustained oxygen release capability of CPO. Furthermore, recent studies indicate that CPO possesses anti-inflammatory and antibacterial effects, which can reduce infection risk during application [112–114]. This is because H_2_O_2_ is generated in the early stage of CPO implantation. Grafting CAT onto the CPO surface can prevent H_2_O_2_ accumulation. A small amount of H_2_O_2_ exhibits antibacterial properties and can avoid causing damage to cells [115]. For example, Huang *et al*. [116] prepared an alginate hydrogel loaded with CPO, which significantly suppressed the proliferation of *Escherichia coli* and *Staphylococcus aureus*. Exposure to the oxygen-carrying alginate hydrogel led to wrinkling and rupture of bacterial surfaces, suggesting severe membrane damage and consequent leakage of intracellular components such as enzymes, proteins and nucleic acids. This demonstrates the antibacterial properties of CPO and its potential as an alternative to antibiotic antimicrobials. In summary, CPO, as a chemical oxygen generator, slowly releases O_2_ upon contact with water, exhibiting strong oxygen production capacity. It simultaneously releases Ca^2+^ and H_2_O_2_, offering dual benefits of promoting osteogenesis and antibacterial activity. Furthermore, surface modification of CPO with PCL and the construction of composite carriers with hydrophobic materials can effectively overcome challenges such as overly rapid reactions and excessive by-products, demonstrating good biocompatibility. For example, Sarkar *et al*. [29] incorporated CPO into PCL-based 3D-printed scaffolds to prepare scaffolds containing 5%, 10% and 20% CPO and cultured human adipose-derived mesenchymal stem cells under normoxic and hypoxic conditions. The results showed that on Day 1, 3 and 5, scaffolds with 5% and 10% CPO exhibited no cytotoxicity and significantly promoted cell proliferation compared to the CPO-free control group. However, the scaffold containing 20% CPO showed slight cytotoxicity, and its proliferation-promoting effect was diminished, indicating that a low concentration of CPO has good biocompatibility. Its *in vivo* biosafety has also been validated. In the *in vivo* subcutaneous implantation study by Xu *et al*. [117], the results showed that at 1 week postimplantation, the material-only group exhibited relatively obvious inflammatory cell infiltration, while the addition of CPO significantly alleviated this inflammatory response. By 3 weeks postimplantation, no obvious inflammatory infiltration or toxic reactions were observed in any group. By achieving controlled oxygen release and precise functionalization, CPO holds application prospects in the fields of tissue engineering and regenerative medicine.

##### Magnesium peroxide

Magnesium peroxide (MPO) also belongs to inorganic solid peroxides. Similar to CPO, this type of material undergoes a chemical reaction with water to generate H_2_O_2_ and Mg^2+^, which subsequently decomposes into water and oxygen. However, unlike CPO, MPO has a slower decomposition rate and a slower oxygen release rate. Consequently, research on MPO-based oxygen-releasing scaffolds is limited. Nevertheless, this characteristic also results in lower cytotoxicity and higher biosafety for MPO [118]. Furthermore, Mg^2+^ is an important divalent ion in the formation of biological apatite, and its presence can promote the bone regeneration process [7, 119, 120]. Studies have shown that Mg^2+^ plays a significant role in promoting osteoblast differentiation and skeletal development [121, 122]. For example, as shown in Figure 4B, Zhang *et al*. [110] developed a PDA-MPO gelatin hydrogel material for tumor therapy and bone repair in rats. The results showed that the material could continuously release oxygen to enhance tumor recurrence suppression and sustainably release Mg^2+^ to promote vascularization and bone regeneration at the defect site. *In vitro* experiments using human bone marrow mesenchymal stem cells (hBMSCs) demonstrated that hydrogels containing 5, 10 and 50 mg/mL MPO exhibited no obvious cytotoxicity at 1, 3 and 7 days. However, the absorbance of the group containing 100 mg/mL MPO was significantly lower than that of the other groups, indicating that an excessively high concentration of MPO had mild cytotoxicity. Further *in vivo* safety evaluation showed that after implanting the hydrogel containing 10 mg/mL MPO into rats for 21 days, no obvious pathological changes were observed in the heart, liver, spleen, lungs or kidneys, indicating that the material did not cause significant toxic reactions *in vivo*. Therefore, MPO exhibits considerable potential for applications in tissue regeneration. Particularly in bone regeneration. Although MPO demonstrates many advantages in tissue repair and regeneration, its application, like that of CPO as an inorganic peroxide, still faces challenges. For instance, excessive H_2_O_2_ generated during the reaction may cause oxidative damage to cells. Therefore, it is necessary to regulate H_2_O_2_ concentration through surface modification or the addition of antioxidants like CAT. Nasrollah *et al*. [120] used poly (lactic-co-glycolic acid) (PLGA) to modify MPO and prepared PLGA/MPO microspheres loaded into a thiolated alginate/methacrylated gelatin photocrosslinked hydrogel. Over a 14-day period, the released magnesium and oxygen concentrations ranged from 13 to 22 mg/L and 7.7–8.2 mg/L. *In vitro* experiments using human amniotic mesenchymal stem cells showed that the PLGA/MPO microsphere hydrogel group exhibited no obvious cytotoxicity at 7, 14 and 21 days, demonstrated significant pro-proliferative effects; and effectively promoted the differentiation of human adipose-derived mesenchymal stem cells into osteoblasts. Although some studies have been conducted on MPO, confirming the cytocompatibility and biosafety of an appropriate amount of MPO, future studies still need to further optimize the oxygen release performance, biocompatibility and long-term safety of MPO to advance its clinical application across different tissue engineering fields. By integrating with other biomaterials and refining surface modification techniques, MPO is poised to assume a greater role in future regenerative medicine.

##### Hydrogen peroxide

H_2_O_2_ is a colorless, transparent liquid peroxide with strong oxidizing properties, biocompatibility and oxygen-release capability. It decomposes into oxygen and water *in vivo* without generating by-products, enhancing the safety of oxygen carriers. It is widely applied in the field of tissue engineering [102, 123]. However, encapsulation or other modifications of H_2_O_2_ are also necessary to prevent direct contact with water, which could release high concentrations of ROS and cause oxidative damage to the organism. As shown in Figure 4C, Ru *et al*. [111] employed a solvent evaporation (W1/O/W2) method to prepare core-shell microspheres, in which H_2_O_2_ served as the oxygen source within the core, encapsulated by an outer layer of PLGA and silk fibroin (SF) grafted with catalase. The oxygen release results are presented in Figure 4D. The study found that 10% SF achieved delayed and controllable oxygen release. In contrast to many oxygen carriers, H_2_O_2_ shows negligible uncatalyzed decomposition. Therefore, in related studies, materials using H_2_O_2_ as the core oxygen carrier often require grafting with catalase to improve efficiency. In the study by Abdi *et al*. [124], H_2_O_2_ microspheres with a PLGA shell were similarly prepared, but alginate was combined on their surface, and catalase was then grafted onto the carboxyl groups of the alginate. The results showed that the higher the alginate concentration, the slower the oxygen release efficiency. Moghassemi *et al*. [123] constructed an oxygenation system comprising liposomes loaded with CAT and liposomes loaded with H_2_O_2_. They found that cells cultured under hypoxic conditions using this oxygenation system exhibited cell viability comparable to that of cells cultured with hydrogels under normoxic conditions. The system effectively alleviated hypoxic damage to cells without generating significant ROS, indicating that the material possesses good cytocompatibility.

#### Sodium percarbonate

Sodium percarbonate (SPC) is a moderately water-soluble salt, which is an adduct formed between H_2_O_2_ and sodium carbonate. Its mechanism of releasing oxygen involves reacting with water to generate H_2_O_2_ and sodium carbonate, which then decompose into water and oxygen [125]. Studies have used it as an oxygen-releasing material, finding that SPC-based oxygen-releasing materials can effectively treat skin wound damage and promote repair [126]. Azari *et al*. [127] used SPC as the oxygen-releasing material, embedding SPC into the intermediate layer of PCL and gelatin to prepare a multilayer electrospun scaffold. This scaffold effectively released oxygen and promoted the synergistic effect of co-culturing keratinocytes and adipose-derived stem cells in enhancing skin tissue regeneration. Waris *et al*^.^ [128] loaded SPC into a chitosan hydrogel via a physical loading method. The study found that the hydrogel containing 2% SPC could sustainably release oxygen, exhibited no obvious cytotoxicity at 3 and 5 days, significantly promoted cell proliferation, and the chick embryo angiogenesis assay indicated that it could promote angiogenesis and accelerate wound healing. In summary, SPC shares similar characteristics with other peroxides. It can be incorporated into hydrogels or used to prepare electrospun scaffolds to avoid O_2_ burst release. However, current research progress has been made in the field of tissue engineering, particularly in skin wound healing. Nevertheless, studies using SPC as an oxygen-releasing material remain scarcely reported. In the future, SPC can be further functionalized to prepare composite scaffolds that match the mechanical properties and regeneration conditions of various tissues, thereby achieving its application.

#### Photosynthetic algae

**Figure 5:**
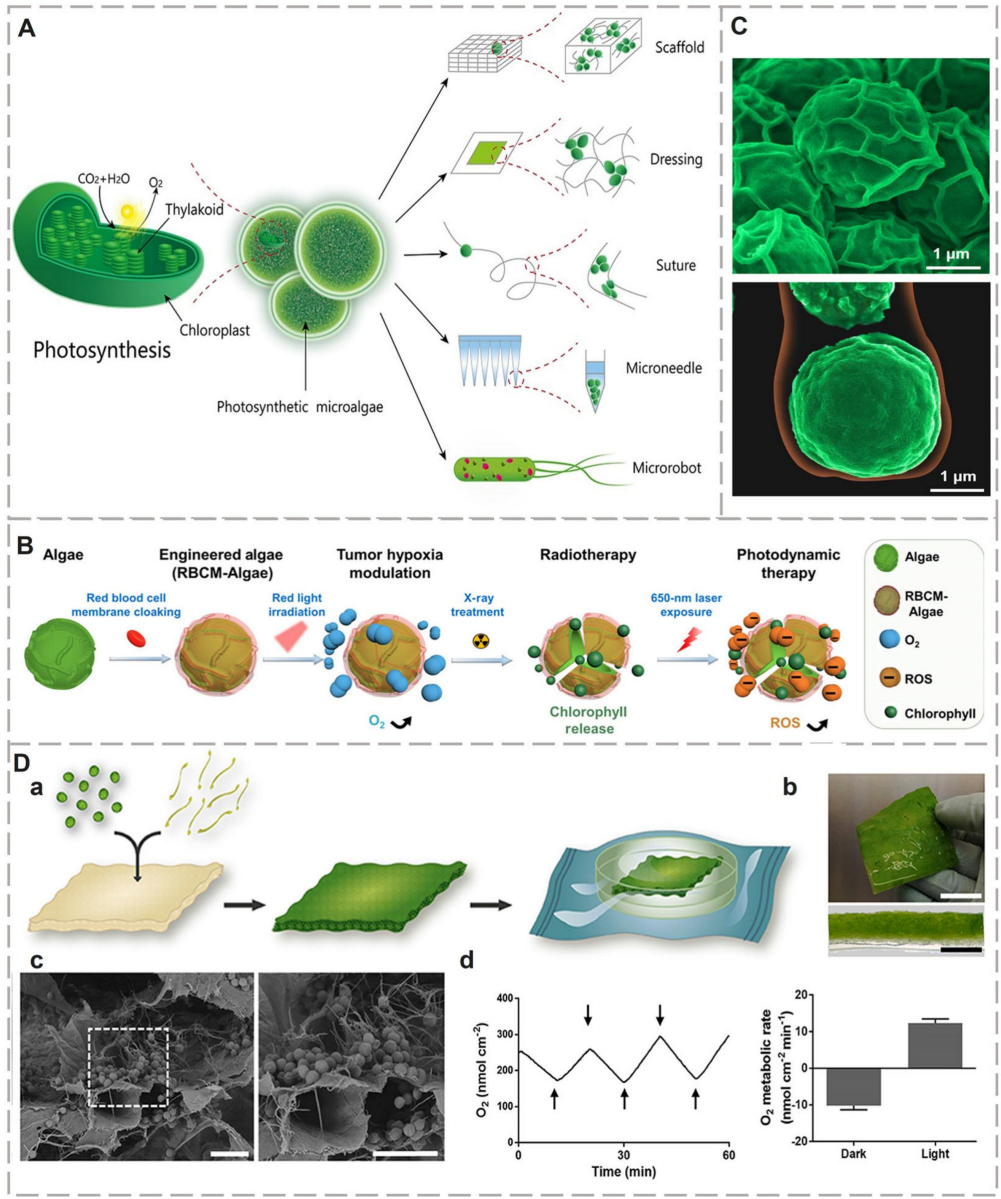
Research progress on photosynthetic algae-based oxygen-releasing scaffolds. (**A**) Principle of oxygen production through photosynthesis and composition of photosynthetic oxygen production systems [129]. Copyright 2024, Oxford Academic. (**B**) Schematic representation of engineered processes and therapeutic interventions, (**C**) Pseudocolor SEM images of algae and RBCM-coated algae [130]. Copyright 2020, AAAS. (**D**) Research results of *Chlamydomonas reinhardtii*. (a) Schematic illustration of scaffold fabrication. (b) Macroscopic view (top) and corresponding cross-sectional image (bottom) of the scaffold. (c) SEM micrograph revealing microalgae uniformly embedded within the fibrin matrix of the scaffold. (d) Dynamic oxygen concentration profiles under alternating dark/light cycles (10 min each, indicated by upward and downward arrows, respectively; left), alongside the corresponding scaffold metabolic rates (right). Scale bars: 2 cm (b, top), 2 mm (b, bottom) and 20 μm (c) [131]. Copyright 2021, Frontiers.

In nature, the light reaction phase of photosynthesis directly generates oxygen, which relies on the thylakoid membranes within chloroplasts. The photosynthetic pigments on their surface absorb light energy, simultaneously releasing oxygen and generating ATP and NADPH. As shown in Figure 5A, photosynthesis-based oxygen production systems constructed on thylakoid membranes are also relatively common in the field of tissue engineering, appearing in different forms such as scaffolds, dressings, sutures and microneedles [129]. Among these, photosynthetic algae like *Chlorella* and *Chlamydomonas reinhardtii* have been extensively studied.

##### Chlorella

Recent studies have demonstrated the efficacy of live algae in ameliorating hypoxia within diabetic wounds, offering superior oxygen delivery efficiency relative to conventional oxygen therapy [83]. *Chlorella* is a natural biological algae with extremely high photosynthetic efficiency and oxygen production capacity far exceeding that of other algae. It can continuously and steadily produce oxygen through photosynthesis, providing sufficient oxygen supply to tissues [130, 132]. *Chlorella* possesses advantages such as simple cultivation conditions, strong proliferation ability, the ability to obtain a large number of *Chlorella* cells in a short time and relatively low cost [133, 134]. Wu *et al*. [135] encapsulated *Chlorella* in a hydrogel. During the day, the live *Chlorella* could reverse severe hypoxia and consume excess ROS, while at night, the *Chlorella* was deactivated to eliminate the side effects of its respiration. The released contents were then used for nutritional supplementation and inflammation relief. As shown in Figure 5B, Qiao *et al*. [130] modified the surface of *Chlorella* with red blood cell membranes to enhance its biocompatibility. The results, visible in Figure 5C, show that *Chlorella* was successfully modified with red blood cell membranes, with its surface enveloped by the membranes, reducing the uptake and clearance of the algae by macrophages. Zhong *et al*. [83] employed biomineralization to modify the *Chlorella* cell surface with a calcium phosphate shell. Positively charged calcium phosphate was applied to the negatively charged *Chlorella* surface via a dip-coating method, improving its stability and biocompatibility. However, as a xenograft, *Chlorella* frequently coexists with bacteria, posing a risk of triggering immune responses and exacerbating oxidative stress, thus restricting its applicability in wound management. It requires modification treatments to reduce its immunogenicity, along with microbial testing [135].

##### Chlamydomonas reinhardtii

*Chlamydomonas reinhardtii*, another type of single-celled algae, has been approved by the U.S. FDA for application in conjunction with collagen-based scaffolds for skin repair, thereby demonstrating favorable biocompatibility [136]. Furthermore, the oxygen release principle of microalgae allows control over oxygen release via light switching, making them suitable for skin and subcutaneous wound therapy. As shown in Figure 5D, Obaíd *et al*. [131] cultured *Chlamydomonas reinhardtii* on a collagen scaffold. SEM images of the hydrogel show the algae evenly distributed within the hydrogel pores, and variations in oxygen release from the scaffold were observed through light-dark cycles. This study also confirmed its safety in clinical trials for treating full-thickness skin wounds. Additionally, as a single-celled organism, *Chlamydomonas reinhardtii* can be custom-optimized for growth rate and oxygen production capacity through methods like genetic engineering. For instance, in the study by Chávez *et al*. [137] transgenic modifications were made to the cell wall-deficient cw15-302-derived UVM4 strain of *Chlamydomonas reinhardtii*, enabling it to secrete VEGF. The safety of its implantation in mice was also verified. As emerging natural oxygen carriers, *Chlorella* and *Chlamydomonas reinhardtii* function like tiny photosynthetic factories, utilizing light energy to produce oxygen *in situ*. They offer the advantage of controllable oxygen release through photosynthesis, along with low cultivation costs and rapid reproduction [129]. Furthermore, the immunogenicity issues of *Chlorella* can be addressed through methods such as red blood cell membrane modification, while *Chlamydomonas reinhardtii* exhibits high biocompatibility and has received FDA approval. However, as single-celled organisms, the forms of oxygen-releasing scaffolds involving algae are mostly limited to hydrogel systems, and their applications are confined to areas such as skin wounds or superficial tissue tumor therapy. Future research should prioritize the advancement of applications for single-celled algae (Table 1).

**Table 1:** Oxygen release mechanisms and advantages/disadvantages of various oxygen-releasing materials.

Category	Material name	Oxygen release principle/mechanism	Advantages	Disadvantages	Applications	Ref.
Oxygen delivery	PFC	High oxygen solubility in the liquid state	No chemical reaction	Short half-life	Nerve regeneration, bone regeneration	[83]
Oxygen delivery	Hb	The iron ion in the Hb subunit binds with oxygen, carrying and delivering it to specific tissues.	High biocompatibility, nontoxic.	Low oxygen concentration	Skin regeneration	[94]
Self-oxygen releasing	CPO	Reacts with water, 2CaO_2_+2H_2_O→2Ca(OH)_2_+O_2_↑	Ca^2+^ promotes bone calcification and bone regeneration.	By-products may pose potential risks, needs resolution, early-stage ROS release requires control.	Releases Ca^2+^, promoting nerve regeneration and bone formation; releases H_2_O_2_, whose antibacterial properties promote skin regeneration.	[29, 116]
Self-oxygen releasing	MPO	Reacts with water, 2MgO_2_+2H_2_O→2Mg(OH)_2_+O_2_↑	Mg^2+^ promotes vascular regeneration and bone regeneration.		Releases Mg^2+^ to promote bone formation; tumor therapy.	[110]
Self-oxygen releasing	H_2_O_2_	Reacts with water, 2H_2_O_2_→2H_2_O+O_2_↑	No by-products		Skin regeneration	[124]
Self-oxygen releasing	SPC	Reacts with water: Na_2_CO_3_⋅1.5H_2_O_2_→Na_2_CO_3_+1.5H_2_O_2_2H_2_O_2_→2H_2_O+O_2_↑	Nontoxic by-products		Nerve regeneration	[126]
Self-oxygen releasing	*Chlorella*	Photosynthesis	Light-controlled oxygen release	Immune rejection remains a challenge to be addressed.	Repair of skin and other superficial tissue injuries.	[130]
Self-oxygen releasing	*Chlamydomonas reinhardtii*	Photosynthesis	FDA-approved; light-controlled oxygen release.	Immune rejection remains a challenge to be addressed.	Repair of skin and other superficial tissue injuries.	[131]

## Oxygen-releasing scaffolds

### Passive oxygen release

#### Modification of oxygen carriers

Among self-oxygen-releasing materials, especially peroxide-based oxygen carriers, there is an issue of burst oxygen release. It is necessary to control the oxygen release amount to avoid tissue damage caused by ROS generated by the oxygen-releasing scaffold. The simplest and most efficient method is to apply a surface coating to the oxygen carrier. Hydrophobic materials such as PCL, PLA and PLLA are typically chosen to regulate the oxygen release amount. Ru *et al*. [111] used H_2_O_2_, which has a high oxygen release capacity, as the oxygen carrier to prepare a core-shell structured oxygen-releasing material via the W_1_/O/W_2_ method. PLGA was used as the shell layer, and H_2_O_2_ was used as the core. By adding 1% PVA to the aqueous phase, the resulting H_2_O_2_-PVP microspheres extended the oxygen release time from 3 to 5 days, slowing down H_2_O_2_ decomposition and achieving delayed O_2_ release. Subsequently, coatings of different concentrations of SF were applied to regulate the release behavior through the degradation of SF into amino acids. The optimal oxygen delay capability was achieved at an SF concentration of 10%. Furthermore, Yang *et al*. [23] used metal-organic frameworks (MOFs) to encapsulate CPO and introduced them into a sodium alginate-acrylamide composite hydrogel system. The experimental results showed that the unencapsulated CPO exhibited the highest oxygen release on Day 2, demonstrating a significant burst-release behavior. Subsequently, the oxygen release trend over 14 days showed no significant difference compared to the hydrogel group without CPO. In contrast, the composite hydrogel loaded with MOF-encapsulated CPO exhibited more sustained oxygen release performance, maintaining continuous oxygen release at low concentrations over 14 days. From Day 4 onwards, its oxygen release level was significantly higher than that of the CPO-free control group and the unencapsulated CPO hydrogel group. These results indicate that MOF encapsulation can effectively mitigate the initial burst release of CPO, achieving a more controlled oxygen release process. In summary, the use of encapsulation strategies can effectively retard the oxygen release rate of oxygen-releasing materials, representing a feasible and effective method for achieving sustained oxygen release.

#### Structural oxygen release

In recent years, research on tissue engineering scaffolds such as electrospun nanofibers, hydrogels and microspheres has been extensive in the field of tissue engineering [138–141]. Among these, electrospun nanofibers can achieve nanofibers with controllable diameters by adjusting electrospinning parameters. The fibrous membranes demonstrate favorable permeability and are capable of emulating the fundamental architecture of the extracellular matrix. Therefore, scaffolds with excellent oxygen release effects can be fabricated by adjusting the physical structure of the nanofibers [142, 143]. Hydrogels possess an interconnected three-dimensional network. These constructs create an aqueous, encapsulated niche conducive to the transport of nutrients and oxygen, which are vital for promoting angiogenesis and tissue regeneration [106, 122]. The micro/nanoscale structure of microspheres provides them characterized by a high surface area relative to volume, enabling efficient loading of cells, drugs or growth factors [144–146].

### Controlled oxygen release

Oxygen serves as an essential requirement for the repair and regeneration of diverse tissues following injury. In recent years, many types of oxygen carriers have been studied. However, damaged tissues often require more oxygen supply in the early stages and a long-term stable supply during the repair process. Yet, the simple oxygen release strategies and uncontrolled oxygen release used in many current studies still struggle to achieve precise regulation of oxygen supply to damaged tissues. Therefore, developing oxygen-releasing scaffolds that can be precisely regulated by changing external conditions is particularly important. In recent years, controlled oxygen release methods have been successively developed [147–150]. Precisely regulating the oxygen release of oxygen-releasing scaffolds holds significant importance for tissue regeneration and repair.

#### Photothermally controlled oxygen release

**Figure 6:**
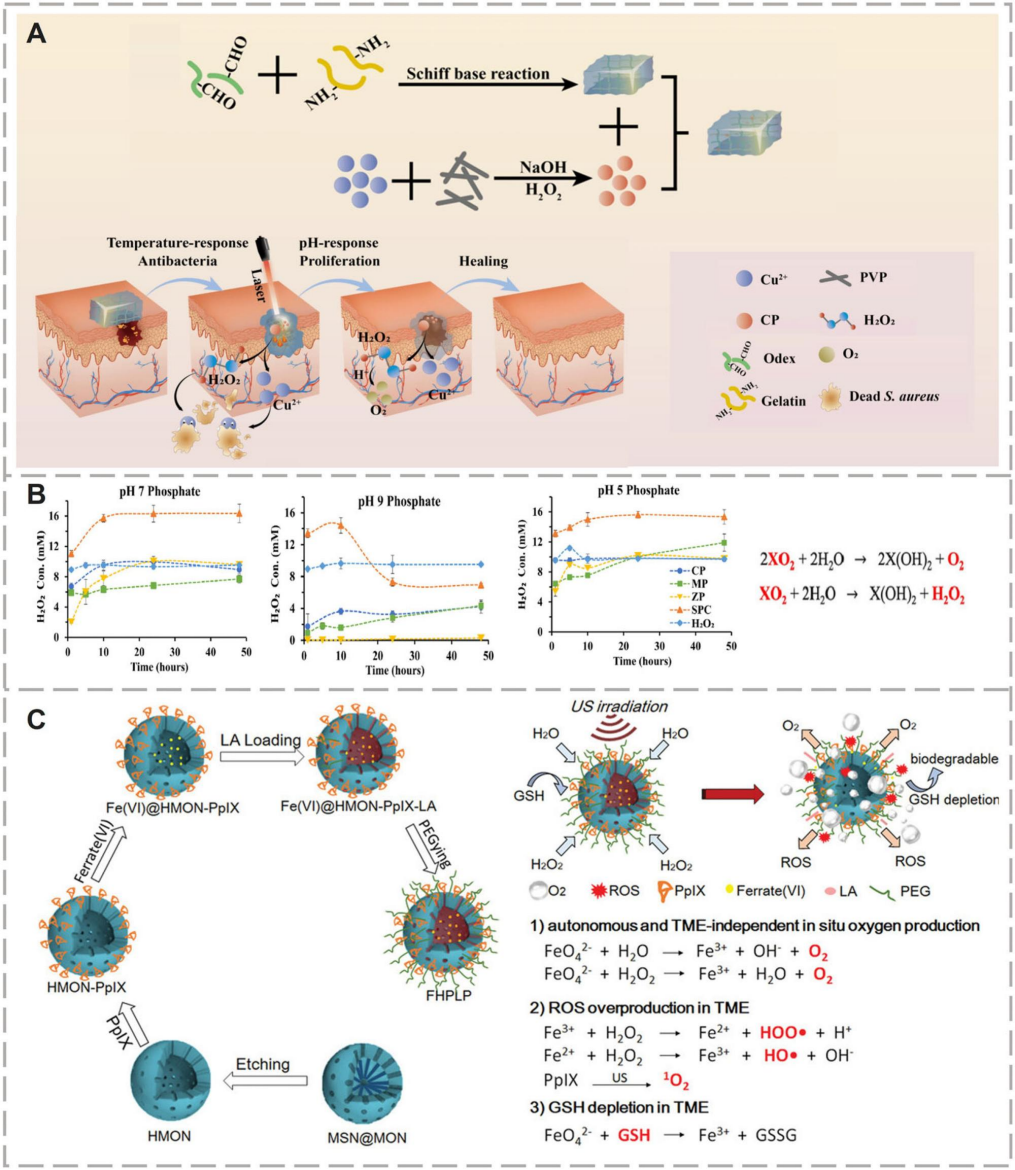
Research progress on controlled oxygen release. (**A**) Schematic diagram of the preparation of photothermal-responsive GGO hydrogel and antibacterial mechanism [154]. Copyright 2024, Elsevier. (**B**) Chemical equations for the reaction of peroxides with water and H_2_O_2_ release curves of different peroxides at pH 5, 7 and 9. (CP: CPO, MP: MPO, ZP: ZnO_2_ and SPC) [155]. Copyright 2022, Elsevier. (**C**) Schematic diagram of the preparation of the US-stimulated FHPLP nanosystem and its oxygen release mechanism [156]. Copyright 2019, Wiley.

Photothermally controlled oxygen-releasing scaffolds typically use photothermal-responsive materials to coat the oxygen carrier or dope them into the main body of the scaffold, achieving a temperature increase through methods such as near-infrared (NIR) irradiation, thereby triggering oxygen release. NIR is widely used in the biomedical field due to its high tissue penetration capability. Experimental results have shown that in rat skin tissue, the penetration depth at 633 nm is 6.3 ± 0.5 mm, while at 705 nm it can reach 7.5 ± 0.5 mm, compared to only 1.0 ± 0.02 mm at a certain lower wavelength, confirming the significant gain effect of wavelength redshift on penetration depth. However, despite the high penetration capability of NIR, its effective penetration depth is still limited by multiple factors. Endogenous chromophores in tissues, such as water, hemoglobin and melanin, absorb and scatter photons, causing the light intensity to decay exponentially with depth. Moreover, the extinction coefficient and conversion efficiency of the photothermal agent itself also directly affect the dosage of heat generation and oxygen release [151]. Therefore, current research on NIR combined with photothermal oxygen-releasing materials is mostly focused on relatively superficial sites such as skin wounds, superficial tumors and shallow bone injuries. For example, black phosphorus nanosheets (BP) are widely used in the fabrication of oxygen-releasing scaffolds due to their high photothermal conversion efficiency, high NIR extinction coefficient and excellent biocompatibility and degradability [147]. Zhang *et al*. [147] prepared a BP-loaded photothermally responsive microneedle patch using Hb as the oxygen carrier. After NIR treatment, BP rapidly converts light energy into thermal energy, increasing local skin temperature, which in turn reduces the oxygen-binding capacity of hemoglobin, leading to controlled oxygen delivery and benefiting skin wound repair. Liu *et al*. [152] combined indocyanine green (ICG) with lauric acid (LA) to coat the surface of CPO, preparing a photothermally controlled oxygen-releasing scaffold. Under NIR irradiation, ICG generates heat, melting the low-melting-point LA coating, exposing CPO to water and thereby triggering oxygen release. This system cleverly utilizes the phase change property of LA to achieve precise regulation of light-controlled oxygen release. PDA has good photothermal conversion efficiency. Wang *et al*. [153] coated the scaffold surface with PDA and loaded it with CPO and CeO_2_ nanoparticles based on PDA’s photothermal properties, preparing a photothermally responsive scaffold material with antitumor and osteogenic effects. As shown in Figure 6A, Shen *et al*^.^ [154] prepared a copper peroxide nanoparticle hydrogel composed of gelatin and oxidized dextran. This hydrogel exhibits good photothermal properties and temperature responsiveness. The copper peroxide nanoparticles generate a photothermal effect upon NIR irradiation and are released, degrading to release Cu^2+^ and H_2_O_2_, which, in combination with PTT can effectively kill bacteria. These studies demonstrate the advantages of photothermal oxygen release systems in spatiotemporal control and noninvasiveness. However, current research using NIR combined with photothermal oxygen-releasing materials mostly focuses on skin and tumor therapy, where oxygen release in deep tissues is difficult to control. In the future, the development of NIR-II responsive materials with strong absorption and high photothermal conversion efficiency at longer wavelengths can be explored to further enhance penetration depth.

#### pH-responsive oxygen release

pH-responsive oxygen-releasing scaffolds can regulate oxygen release capacity based on changes in the surrounding environmental pH. In recent years, they have attracted considerable interest in fields including tissue engineering, wound healing and tumor therapy [154, 157, 158]. These materials achieve oxygen release by utilizing chemical or physical changes of oxygen-releasing carriers (such as CPO, MPO, etc.) or pH-responsive materials under specific pH conditions, thereby meeting the needs of different physiological environments. For solid peroxides, their hydrolysis rates vary under different pH conditions, thereby regulating oxygen release. Under acidic conditions, the reaction between the peroxide and water is accelerated, producing H_2_O_2_ and the corresponding metal hydroxide. This may provide sustained release of H_2_O_2_, endowing the scaffold with antibacterial properties, thus making it suitable for oxygen release in acidic microenvironments such as tumor tissues or infected wounds. Different peroxides exhibit significant differences in pH sensitivity. MPO and ZnO_2_ are notably affected by pH, as shown in Figure 6B, in the study by Rastinfard *et al*. [155], the H_2_O_2_ release amounts from different oxygen carriers under various pH conditions were compared. The results showed that at pH 5, compared to other peroxides, MPO and ZnO_2_ released more H_2_O_2_ in an approximately linear manner, while at pH 9, their behavior was similar to that at pH 7, with zinc peroxide releasing almost no H_2_O_2_. Additionally, Peng *et al*. [159] developed a gelatin-phenylboronic acid-PVA-MPO composite hydrogel. Due to the formation of a boronic ester bond with dynamic formation and dissociation characteristics between PVA and phenylboronic acid, the hydrogel exhibited pH-dependent release of H_2_O_2_ and Mg^2+^, with sustained release at physiological pH (7.4) and significantly increased release at infection pH (5.5). This study also revealed the potential advantages of pH-responsive oxygen release. In the superficial-to-deep interface of bone tissue infection, pH-triggered oxygen release can achieve dual functions of on-demand antibacterial activity and oxygen supply at the local infection site. However, this study also indicated that the spatial range of its osteogenic and antibacterial effects is mainly limited to the peripheral area surrounding the scaffold implantation, whereas the central region of deep bone defects still requires longer-term sustained oxygenation support. In addition, pH-responsive polymers undergo swelling, degradation or conformational changes at specific pH values, thereby regulating drug release [158]. Shen *et al*^.^ [154] prepared a copper peroxide nanoparticle hydrogel (GGO) composed of gelatin and oxidized dextran. The hydrogel is formed via a Schiff base reaction involving gelatin and oxidized dextran. When the pH is less than 6, owing to the formation of the Schiff base, the hydrogel exhibits pH responsiveness, rapidly degrading and releasing copper peroxide. Subsequently, the released O_2_ and Cu^2+^ promote angiogenesis and accelerate wound healing. In summary, the core advantage of pH-responsive oxygen release strategies lies in their ability to spontaneously induce oxygen generation and release in response to pH changes in the physiological microenvironment, without requiring external secondary intervention, thus offering unique application value in specific pathological scenarios such as infected wounds and tumors. The limitation of pH-responsive oxygen-releasing scaffolds is that pH is dynamically changing during the *in vivo* injury and repair process, and the validation of their actual performance in the complex *in vivo* environment remains limited.

#### US-responsive oxygen release

Low-frequency US enables spatiotemporal control of drug release and has been widely reported [160, 161]. In recent years, there have also been relevant reports on the US in the field of oxygen-releasing scaffolds. Song *et al*. [162] prepared a composite microsphere system using SiO_2_, MnO_2_, PLGA, etc. They used US to promote oxygen release from within the microspheres, thereby effectively reversing the hypoxic environment in the bone injury microenvironment. Fu *et al*. [156] prepared an FHPLP nanodelivery system for tumor therapy. US stimulation offers stronger penetration and is accompanied by heat generation. US induction can raise the temperature of the nanosystem, thereby triggering the temperature responsiveness of LA. The ferrite in the FHPLP nano system reacts with H_2_O, H_2_O_2_ or GSH in the tumor microenvironment, producing excess O_1_ to kill tumor cells and provide sufficient. Compared to photodynamic therapies such as NIR, US-mediated sonodynamic therapy has a broader application scope and stronger penetration capability, making it more suitable for deep tissue injury repair (Figure 6C).

### ROS scavenging

**Figure 7:**
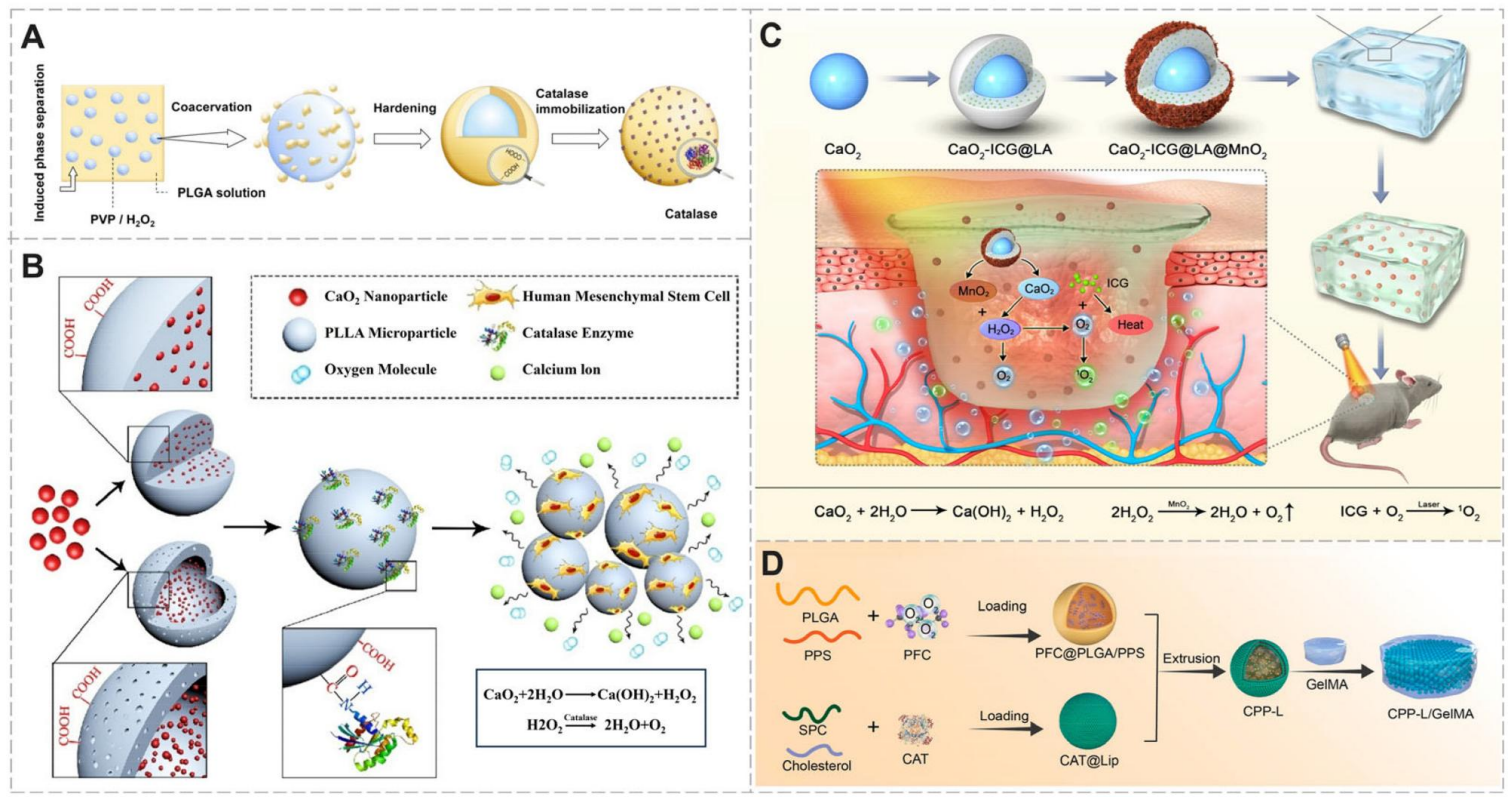
ROS scavenging strategies. (**A**) Schematic diagram of catalase grafting onto the surface of PLGA-H_2_O_2_/PVP microspheres [115]. Copyright 2016, Elsevier. (**B**) Schematic illustration of the mechanism for catalase immobilization on the surface of PLLA-CPO [109]. Copyright 2020, Elsevier. (**C**) Schematic diagram of a MnO_2_-grafted photothermally controlled oxygen-releasing hydrogel for skin wound injury treatment [4]. Copyright 2023, Springer Nature. (**D**) Schematic illustration of the preparation process for PLGA and PPS-co-modified PFC microspheres with surface-immobilized CAT [41]. Copyright 2023, Elsevier.

ROS are derived from O_2_ and are formed through oxidation reactions or electron excitation. They are predominantly composed of hydroxyl radicals (•OH), singlet oxygen (^1^O_2_) and superoxide anions (O_2_•^-^). When ROS levels surpass a critical threshold, they induce oxidative damage to cellular components, thereby impeding cell growth [163, 164]. During the oxygen release process, excessive H_2_O_2_ may be generated, which can adversely affect cells and tissues. To improve the safety of oxygen-releasing scaffolds, researchers often combine oxygen carriers with materials such as MnO_2_, CAT or ascorbic acid to promptly scavenge excess ROS [150, 164, 165]. As shown in Figure 7A, Montazeri *et al*. [115] immobilized CAT on oxygen-generating microparticles with a H_2_O_2_/PVP core and a PLGA shell to catalyze the decomposition of H_2_O_2_ into H_2_O and O_2_. The enzyme was stably anchored via covalent bonding with carboxyl groups on the PLGA polymer chains, retaining up to 40% of its initial activity over a period of 14 days. As shown in Figure 7B, Mohseni-Vadeghani *et al*. [109] similarly used CAT as an ROS scavenger by grafting CAT onto the surface of CPO microspheres coated with poly(L-lactic acid) (PLLA). Cytotoxicity results showed that CAT grafting significantly reduced the cytotoxicity of the oxygen-releasing microspheres, whereas CPO microspheres without CAT modification exhibited less than 75% cell viability. However, MnO_2_ is also commonly used as an H_2_O_2_ decomposition catalyst for ROS scavenging. For example, Li *et al*. [4] developed a hydrogel-based photothermal-responsive oxygen release platform where catalyst MnO_2_ was coated on the surface of CPO to enhance the decomposition rate of H_2_O_2_. A schematic diagram is shown in Figure 7C, thereby reducing oxidative stress in cells. Additionally, polyphenylene sulfide (PPS) is a hydrophobic substance. The sulfide moiety in the central region of PPS can undergo stepwise oxidation upon ROS exposure, progressing from sulfide to sulfoxide and finally to sulfone. This transformation increases the hydrophilicity of the initially hydrophobic core. This can drive material decomposition and lead to drug release [166]. As shown in Figure 7D, Sun *et al*. [41] co-modified PFC with PLGA and PPS, grafted CAT on the surface, and incorporated it into a GelMA gel. They found that CAT release could degrade H_2_O_2_ to produce oxygen. Moreover, excess ROS triggered PPS, promoting sustained oxygen release for over 2 weeks.

## Applications of oxygen-releasing materials in tissue engineering

Hypoxia during tissue regeneration and repair represents a major challenge in tissue engineering. Although different implant scaffolds, such as hydrogels, membranes and cell-loaded constructs, have been developed, most lack regulation of oxygen in the injury microenvironment, making it difficult to achieve ideal therapeutic outcomes. Oxygen-releasing scaffolds facilitate oxygen delivery to impaired tissues, thereby promoting angiogenesis and tissue regeneration at the injury site [32, 112, 156, 167]. Oxygen is essential for sustaining cell survival, proliferation, differentiation and tissue regeneration. Currently, large-sized implants (>1 mm³) often suffer from hypoxia in central regions due to limited oxygen diffusion, leading to cell apoptosis and reduced therapeutic efficacy of the graft. Chronic wounds, cardiovascular injuries, bone defects and solid tumor microenvironments also suffer from severe hypoxia, hindering healing and treatment. Through sustained and controllable oxygen release, oxygen-releasing materials can effectively alleviate local hypoxia, promote angiogenesis, collagen synthesis and antibacterial activity, and reshape the local hypoxic microenvironment, providing innovative solutions for skin regeneration, bone repair, nerve reconstruction, cardiovascular repair and tumor therapy. They have become a research hotspot in tissue engineering. Table 2 lists the applications of oxygen-releasing materials in tissue engineering.

### Skin tissue engineering

Chronic wounds are hindered by a hypoxic microenvironment that impairs epithelialization and angiogenesis. Oxygen-releasing scaffolds provide a new strategy to accelerate wound healing by improving local oxygenation, promoting collagen deposition and enhancing angiogenesis. Shiekh *et al*. [91] encapsulated CPO in polylactic acid microspheres and co-loaded exosomes to prepare a CPO composite hydrogel, achieving 14 days of sustained oxygen release and significantly enhancing collagen deposition and angiogenesis while reducing oxidative stress levels. Azaria *et al*. [71] embedded SPC into PCL/gelatin multilayer scaffolds via electrospinning, promoting the proliferation and migration of a keratinocyte-adipose-derived stem cell co-culture system and accelerating full-thickness skin defect regeneration. Waris *et al*. [72] loaded SPC into a chitosan hydrogel and used a polyurethane coating to achieve sustained oxygen release. This system demonstrated good antibacterial effects while continuously promoting angiogenesis. By increasing local oxygen partial pressure, it activated antibacterial immune responses and broke the hypoxia-infection vicious cycle. In addition to single-function oxygen release, multifunctional systems integrating physical barriers and inflammatory regulation have also shown unique advantages. In the study by Li *et al*. [4], a composite hydrogel system consisting of CPO-ICG-LA-MnO_2_ loaded in a PDA-hyaluronic acid hydrogel was prepared. The hydrogel could form a physical barrier on the wound surface while controllably releasing oxygen. The ROS generated during the early stage of wound healing effectively inhibited the inflammatory burst, and the controlled and sustained oxygen supply promoted the regeneration and repair of full-thickness skin wounds in rats. Han *et al*. [168] developed an oxygen-loaded nanobubble hydrogel. By actively regulating the stage-specific healing cascade, it significantly downregulated the expression of inflammatory factors IL-6 and TNF-α, effectively alleviated tissue hypoxia, accelerated complete wound closure by more than 25%, and reduced the final scar width by over 50%, thereby accelerating the closure of surgical wounds in rats and promoting the restoration of native skin structure. Krishnadoss *et al*. [169] used a biocompatible ionic liquid (BIL) and gelatin methacrylamide to prepare a smart self-oxygenating platform. This platform generates oxygen by electrochemically decomposing water molecules at the electrode interface, with the release rate adjustable by regulating the current. In a diabetic mouse wound model, it promoted angiogenesis and collagen deposition under hypoxia, accelerating wound healing. Collectively, these studies demonstrate that scaffolds with oxygen-releasing capability can effectively regulate the oxygen microenvironment during skin tissue repair and promote skin healing.

### Bone tissue engineering

Large-sized bone defect repair faces challenges of osteoblast apoptosis and impaired vascularization due to deep hypoxia. To address this challenge, researchers have developed various functionalized bone repair materials that integrate oxygen release, ROS scavenging and immune regulation into one system. Han *et al*. [41] co-loaded CAT and ROS-responsive oxygen-releasing nanoparticles (PFC@PLGA/PPS) into liposomes and then combined them with GelMA hydrogel for bone defect treatment. This system continuously scavenged ROS and synergistically provided oxygen, promoting bone regeneration, inhibiting osteoclast generation and achieving regulation of the bone immune microenvironment. Hwang *et al*. [170] prepared PFO hollow microparticles using a W/O/W emulsion method. The results showed they could significantly prolong the survival and differentiation capacity of human periosteum-derived osteoblasts under hypoxia. Zhang *et al*. [171] applied a PLLA coating to strontium peroxide (SrO_2_)-modified titanium (Ti) implants. They found this enhanced bone defect repair capability, improved osseointegration, mitigated the inhibitory effect of the hypoxic microenvironment on bone regeneration, and provided immunomodulatory functions. Peng *et al*. [118] used 3D-printing technology to composite PCL, β-tricalcium phosphate (β-TCP) and MPO into a scaffold (PCL/β-TCP/MPO). The study found it could sustain oxygen release, activate the osteogenic differentiation pathway in BMSCs and significantly improve the survival, proliferation, migration and osteogenic differentiation capacity of BMSCs, promoting bone defect repair. Yang *et al*. [23] used CPO as an oxygen carrier to construct a self-oxygenating, 3D-printed active hydrogel scaffold. The pore structure fabricated via 3D-printing facilitates stable oxygen diffusion within the scaffold. Furthermore, the incorporation of CPO endows the scaffold with sustained oxygen supply capability, effectively promoting angiogenesis and osteogenic differentiation under hypoxic conditions. In another study, Liu *et al*. [172] combined oxygen release with topographical structure to design a 3D-printed oxygen-releasing scaffold based on photocrosslinked SF, lithium magnesium silicate (XLG) and CPO. *In vitro* and *in vivo* experiments showed that this scaffold upregulated the expression of VEGF, CD31 and the key osteogenic proteins RUNX2 and BMP-2, promoting collagen production and calcium deposition. The synergistic effect of the scaffold’s topographical structure and oxygen release induced neovascularization and osteogenic differentiation. Current oxygen-releasing scaffolds for bone repair have evolved into integrated functional scaffolds incorporating ROS scavenging, immune regulation and osteogenesis promotion, holding great application prospects in the field of bone defects.

### Neural tissue engineering

In the early phase after neural tissue injury, adequate and timely oxygen supply for vascular network reconstruction is crucial for preventing tissue necrosis and ensuring cell survival [173]. Haki *et al*. [106] found that a CPO combined with SF combined with brain decellularized extracellular matrix (dECM) composite hydrogel could provide neuroprotective oxygen release for over 2 weeks, promoting primary neuronal synaptic extension and reducing hypoxic apoptosis. This system mimics the mechanical properties of brain tissue, synergistically combining oxygen along with bioactive factors to facilitate the directed differentiation of neural stem cells and establish a biomimetic microenvironment conducive to brain injury repair. Photoresponsive oxygen release systems can regulate oxygen release from materials in the early phase after neural injury. Wei *et al*. [174] prepared a *Chlorella* hydrogel and found that light response enabled controlled oxygen release, suppressing early inflammation and promoting myelin regeneration. RNA sequencing identified upregulation of the phosphatidylinositol 3-kinase-protein kinase (PI3K-Akt) and calcium ion (Ca^2+^) signaling pathways, which may be related to oxygen release. In addition, oxygen plays a regulatory role in the immune microenvironment. Furthermore, Liu *et al*. [167] also demonstrated that oxygen supply could effectively promote the polarization of M1-type macrophages to M2-type macrophages. An albumin-biomimetic cerium oxide nanoparticle (CeO_2_@BSA nanoparticle, CeNPs) was dispersed in gelatin methacryloyl to obtain an ROS-scavenging hydrogel (CeNP-Gel). Through the hydrogel’s sustained-release system, the hydrogel progressively mitigated the oxidative stress microenvironment and the oxygen released therefrom enhanced the viability of encapsulated neural stem cells, acting synergistically to promote neural repair. In terms of temporal regulation, Ma *et al*. [175] used PFTBA as an oxygen carrier to prepare a coaxial electrospun temporally ordered oxygen-releasing scaffold with PCL as the shell and PFTBA plus VEGF as the core, achieving sequential release of oxygen and VEGF. This scaffold provided sustained oxygen supply to Schwann cells *in vitro*, protecting them from hypoxia. *In vivo* experiments showed that after the oxygen carried by PFTBA was depleted, VEGF induced neovascularization and promoted nerve regeneration. This indicates that oxygen-releasing scaffolds hold application potential in the field of neural tissue engineering for repair and regeneration.

### Cardiovascular tissue engineering

Restoration of oxygen supply following cardiovascular tissue ischemia is critical for cardiomyocyte survival and functional reconstruction. To address this need, researchers have developed various forms of oxygen-releasing systems. For example, Zhang *et al*. [48] prepared microspheres with a PLGA shell encapsulating H_2_O_2_ and PVP (PLGA-encapsulated H_2_O_2_/PVP) via an emulsification method, combined with CAT to achieve on-demand oxygen release through catalytic decomposition of H_2_O_2_. Under hypoxic conditions, this system sustainably released oxygen, improving stem cell survival and cardiomyocyte differentiation efficiency. Building on this, a platelet membrane coating-targeting modification strategy [98] further enhanced the enrichment of the microspheres in the ischemic area and improved cardiac function parameters. Hassan *et al*. [176] developed an injectable, self-oxygenating SF-tyramine-alginate hybrid hydrogel using H_2_O_2_ as the oxygen carrier. This system locally and sustainably released stromal cell-derived factor-1α (SDF-1α) and oxygen. Under 0.5% O_2_ hypoxic conditions, the survival rate of endothelial cells cultured in this system exceeded 90% and the survival rate of cardiomyocytes increased by 30%. In a rat myocardial infarction model, this hydrogel significantly reduced the fibrotic scar area, improved left ventricular systolic and diastolic function by approximately 10% and 20%, respectively, and increased ejection fraction by about 25% on Day 7. The synergistic delivery of oxygen supply and pro-angiogenic factors achieved better therapeutic outcomes than oxygen supply alone, as it actively recruited endogenous repair cells while improving local oxygenation. Electrical conductivity can be combined with oxygen release strategies. Zhao *et al*. [177] used PCL and gelatin to encapsulate SrO_2_, preparing an ion-conductive hydrogel that gradually released oxygen and strontium ions. This hydrogel improved cell survival under hypoxia. Strontium ions promoted microvascular formation, and the incorporation of conductive materials protected ischemic tissue from oxidative stress damage, effectively supporting cardiac function improvement. Furthermore, Wang *et al*. [178] adopted a natural oxygen supply strategy, encapsulating cyanobacteria in a biocompatible hydrogel. The cyanobacteria continuously produced and released oxygen through photosynthesis, and the oxygen release rate could be precisely controlled by adjusting the light intensity and duration. After implantation, the oxygen released from this photosynthetic hydrogel induced local vascular growth, providing a sustainable, external oxygen source-free strategy for vascularized tissue engineering applications.

### Tumor therapy

The hypoxic microenvironment within solid tumors not only drives malignant progression but also reduces sensitivity to radiotherapy, chemotherapy and photodynamic therapy. To overcome this challenge, various oxygen-generating therapeutic nanoplatforms have been developed in recent years. Xavierselvan *et al*. [99] loaded PFP nanodroplets with the photosensitizer benzoyl peroxide, which undergoes a liquid-to-gas phase transition under NIR excitation, simultaneously releasing oxygen and enhancing photodynamic efficacy. However, oxygen regulation in tumor therapy requires balancing a dual effect: local oxygen sensitization can improve radiotherapy and phototherapy efficiency, but excessive oxygen supply may promote tumor proliferation. Therefore, designing smart oxygen-releasing systems that integrate stimulus-responsive mechanisms to achieve on-demand and controlled oxygen delivery has become an important development direction in this field. Hoang *et al*. [179] used stop-flow lithography (SFL) to prepare acid-degradable Janus-type multicompartment carriers capable of separately encapsulating piezocatalytic gold nanoparticle-coated polyethylene glycol-modified ZnO nanorods (Au@P-ZnO NRs) and generating CAT. During the SFL process, by adjusting the composition ratio of acid-cleavable monomers in the precursor solution, CAT and Au@P-ZnO NRs were released sequentially. Since CAT provides O_2_, the sequential release from the Janus carriers under US irradiation in hypoxic conditions significantly increased intracellular ROS levels. The study found that a single intratumoral injection of Janus particles encapsulating CAT and Au@P-ZnO NRs effectively alleviated tumor hypoxia and significantly inhibited tumor growth. This study revealed the significant potential of sequential release. Furthermore, sonodynamic therapy (SDT), which offers higher penetration intensity, has also been applied in tumor therapy. It uses a US to trigger sonosensitizers to detect excessive ROS (mainly singlet oxygen, ^1^O_2_) production and subsequent oxidative cell damage. For example, Fu *et al*. [156]used K_2_FeO_4_ as an oxygen-generating agent, loaded it into hollow mesoporous organosilica nanomaterials, anchored the organic sonosensitizer protoporphyrin (IX, PpIX) on the nanomaterial surface and prepared the FHPLP nano-system via LA and PEG modification. Using US stimulation as an adjuvant therapy, the results showed that under hypoxic conditions, the FHPLP nanosystem significantly enhanced ROS production and the more effective tumor inhibitory effect of SDT on osteosarcoma, while also significantly upregulating the tumor suppressor protein p53. Zhong *et al*. [83] combined photodynamic therapy with laser-induced photothermal therapy and used Chlorella as an oxygen-producing agent, with chlorophyll serving for fluorescence imaging to assist tumor treatment. Under NIR light irradiation at 650 nm, this approach effectively promoted ROS release and eliminated cancer cells. Second, biological oxygen-generating strategies that do not rely on photodynamic therapy or sonodynamic therapy offer alternative approaches for hypoxic tumor treatment. Tian *et al*. [180] developed a novel metal-free Type I photosensitizer with peroxidase-like activity, namely N-doped carbon dots/mesoporous silica nanoparticle (NCDs/MSN, ≈40 nm) hybrids. NCDs/MSN can simultaneously generate O_2_•^-^, •OH and O_2_ through an electron transfer process under 640 nm light irradiation, effectively enabling synergistic treatment of hypoxic tumors via Type I PDT and peroxidase-like catalytic activity. In addition, oxygen supply can synergistically enhance chemotherapy, further improving tumor treatment efficacy. Chen *et al*. [181] used temozolomide as the drug and hollow manganese dioxide (HMnO_2_) as the carrier material, coated with PDA Temozolomide and RAP-12 peptide were grafted onto the outer layer to further enhance stability and brain targeting. Under NIR irradiation and in the presence of glutathione and H_2_O_2_, MnO_2_ released O_2_, alleviating the hypoxic environment at the tumor site and further promoting the release of TMZ, thereby exerting chemotherapeutic effects. This demonstrates that oxygen can promote drug efficacy and facilitate tumor treatment. In summary, the application of oxygen-releasing scaffolds in tumor therapy has evolved into diverse composite systems integrated with chemotherapy, photodynamic therapy, sonodynamic therapy and photothermal therapy. In the future, achieving spatiotemporally precise regulation of oxygen release while avoiding potential pro-tumor risks and realizing smart oxygen release that adapts to the microenvironment will be key directions for advancing clinical translation in this field (Table 2).

**Table 2:** Applications of oxygen-releasing materials in tissue engineering.

Oxygen-releasing material	Application	Construction method	Therapeutic effect	Ref.
H_2_O_2_	Tissue engineering (nerve, muscle, retina, bone)	Encapsulate H_2_O_2_ in PLA microspheres, then mix it into oxidized pectin and grafted gelatin to form a hydrogel	Provides sustained oxygen release for 14 days, improving regeneration of electrically responsive tissues	[182]
Nanobubbles	Skin tissue	Oxygen-loaded nanobubble hydrogel	Downregulates IL-6 and TNF-α, alleviates hypoxia, accelerates wound closure and promotes skin healing	[168]
CPO	Diabetic chronic wound healing	Prepare an oxygen-releasing hydrogel containing antioxidant polyurethane (PUAO) and CPO, loaded with adipose-derived stem cell (ADSC)-derived exosomes	Promotes wound healing, reduces oxidative stress, induces angiogenesis, enhances collagen remodeling, prevents infection.	[183]
CPO	Bone tissue	3D-printed oxygen-releasing scaffold based on photocrosslinked silk fibroin, lithium magnesium silicate (XLG) and CPO	This scaffold upregulates the expression of VEGF, CD31 and the key osteogenic proteins RUNX2 and BMP-2, promoting collagen production, calcium deposition, vascularization and osteogenic differentiation.	[172]
CPO	Neural tissue engineering	Prepare an oxygen-releasing silk fibroin hydrogel containing CPO particles and ECM	Sustains oxygen release for over 2 weeks, effectively promoting cell growth under hypoxic conditions.	[106]
SPC	Skin Tissue Engineering	Prepare a multilayer electrospun scaffold embedding SPC in the intermediate layer of PCL and gelatin	Effectively releases oxygen, promotes co-culture of keratinocytes and adipose-derived stem cells, accelerates skin tissue regeneration.	[127]
SPC	Diabetic wound healing	Prepare PCL nanofibers loaded with SPC via electrospinning	Sustained oxygen release, promotes angiogenesis, accelerates diabetic wound healing.	[125]
SPC	Chronic wounds	Load SPC into a chitosan hydrogel and use a polyurethane coating to achieve sustained oxygen release	Sustained oxygen release, promotes angiogenesis, good antibacterial effect, accelerates wound healing.	[128]
PFC	Bone tissue repair	Co-load CAT and ROS-responsive oxygen-releasing nanoparticles (PFC@PLGA/PPS) into liposomes, then combine with GelMA hydrogel	Continuously scavenges ROS, provides oxygen, promotes bone regeneration, inhibits osteoclast generation.	[41]
PFO	Bone tissue repair (mandibular osteomyelitis)	Seed human periosteum-derived osteoblasts (hPDCs) on hollow microparticles loaded with PFO	Promotes bone regeneration, improves bone defect repair outcome, enhances bone tissue mineralization and angiogenesis.	[170]
PFO	Bone tissue repair	Prepare hollow microparticles (HPs) loaded with PFO via W/O/W emulsion solvent evaporation	Prolongs cell survival under hypoxic conditions, maintains osteogenic differentiation capacity of cells, promotes bone regeneration.	[91]
H_2_O_2_	Myocardial infarction	Enzyme crosslinking via horseradish peroxidase and H_2_O_2_ to prepare a composite injectable hydrogel with alginate, SF and tyrosine	Significantly reduces fibrotic scar formation and significantly improves left ventricular systolic and diastolic function	[176]
*Cyanobacteria*	Vascular tissue	Cyanobacteria are encapsulated in a biocompatible hydrogel, continuously producing and releasing oxygen through photosynthesis to promote vascular formation.	The oxygen release can be controlled by adjusting light intensity and duration. After implantation, the oxygen released from this photosynthetic hydrogel induces local vascular growth.	[178]
SrO_2_	Bone tissue repair/bone defect treatment	Decorate Ti implant surface with SrO_2_ via a PLLA coating	Enhances bone defect repair capacity, improves osseointegration, alleviates the inhibitory effect of hypoxic microenvironment on bone regeneration, while providing immunomodulatory function.	[171]
MPO	Bone tissue repair (large bone defect repair)	Prepare a scaffold by compositing PCL, β-tricalcium phosphate (β-TCP) and MPO using 3D-printing technology	Significantly improves survival, proliferation, migration and osteogenic differentiation capacity of BMSCs, promotes bone defect repair.	[118]
MPO	Bone tissue repair	Prepare a composite scaffold of PCL, nano-hydroxyapatite (nHA), MPO and PDA using 3D-printing technology	Possesses sustained oxygen release and enhanced photothermal therapeutic effects, enabling simultaneous osteosarcoma cell killing and bone defect repair.	[184]
H_2_O_2_	Myocardial infarction therapy	Prepare microspheres encapsulating H_2_O_2_ and PVP via emulsification, use PLGA as the shell to form a core-shell structure, then combine with a thermosensitive hydrogel	Sustained oxygen release under hypoxic conditions, improves stem cell survival and cardiomyocyte differentiation efficiency.	[44]
H_2_O_2_	Myocardial infarction therapy	Mix oxygen-releasing microspheres (containing H_2_O_2_ and CAT) with a thermosensitive hydrogel to form an injectable hydrogel	Sustained oxygen release, significantly improves cardiomyocyte survival, reduces myocardial fibrosis, improves cardiac function.	[185]
H_2_O_2_	Myocardial infarction therapy	Form microspheres by encapsulating an H_2_O_2_/PVP complex with PLGA, immobilize CAT on the surface, then modify with platelet membrane and targeting peptides	Sustained oxygen release, promotes cardiomyocyte survival and angiogenesis, improves cardiac function.	[186]
PFP	Head and neck cancer therapy	Prepared via water-emulsification method, encapsulating benzoyl peroxide (BPD) in nanodroplets containing PFP	Alleviates tumor hypoxia, enhances photodynamic therapeutic effect.	[187]
MnO_2_	Glioblastoma	Coat MnO_2_ with PDA, then externally graft RAP-12 peptide and temozolomide	Effectively inhibits glioblastoma growth while prolonging survival rate in nude mice.	[180]
K_2_FeO_4_	Osteosarcoma	Use K_2_FeO_4_ as an oxygen-generating agent, load it inside hollow mesoporous organosilica nanomaterials, prepare FHPLP nano-system via LA and PEG modification	Even under hypoxic conditions, the FHPLP nano-system significantly enhances ROS production and the more effective tumor inhibitory effect of SDT against osteosarcoma and significantly upregulates tumor suppressor protein p53.	[156]
CPO, CeO_2_	Jaw bone regeneration, tumor therapy	Coat the surface of a 3D-printed scaffold with PDA and load CPO and CeO_2_ particles	The scaffold exhibits effective osteoinductivity, inducing osteoblast differentiation and promoting osseointegration.	[153]
*Chlorella*	Facial nerve	Use photoresponse to control the release of *Chlorella* in a hydrogel for facial nerve injury therapy	The scaffold can modulate oxygen release and inflammation via photoresponse, enhancing myelin regeneration.	[174]

## Big data analysis of oxygen-releasing materials and tissue engineering in the past 5 years

**Figure 8:**
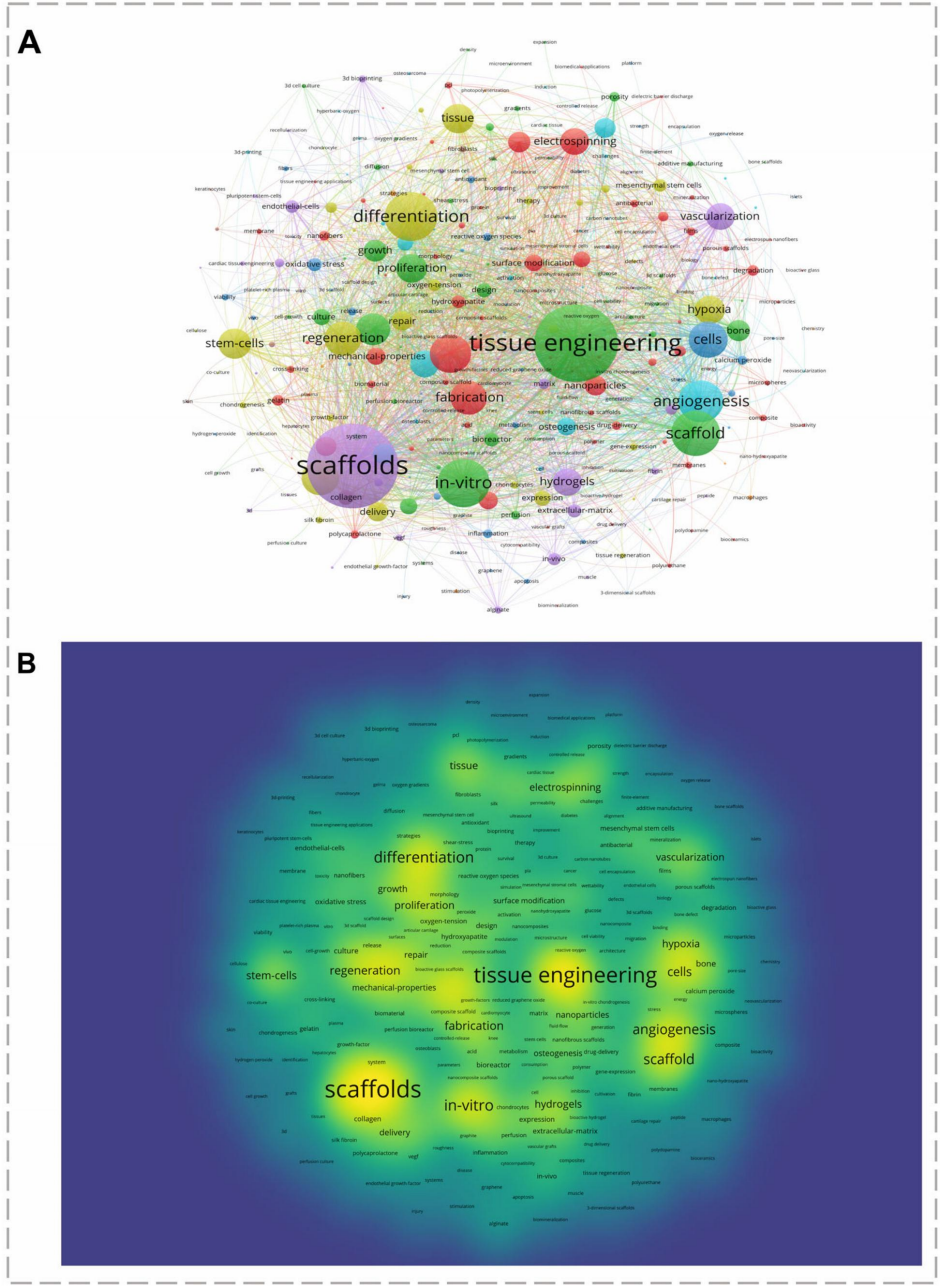
Visualization analysis of literature search on oxygen-releasing materials and tissue engineering. (**A**) Network visualization analysis map of the field of oxygen and tissue engineering. (**B**) Density visualization analysis map of the field of oxygen and tissue engineering. Created with VOSviewer.

Literature related to oxygen and tissue engineering was retrieved from the Web of Science database. A total of 1363 publications from the past five years were screened for keyword co-occurrence analysis using VOSviewer software. After filtering, 456 keywords were analyzed. The resulting network visualization analysis is shown in Figure 8A, illustrating the relationships and cluster structure among keywords. High-frequency keywords were 3D printing (151 times), 3D cell culture (117 times), angiogenesis (60 times), antibiosis (46 times) and bone regeneration (28 times). It can be observed that 3D culture research accounts for a significant proportion. The density visualization map (Figure 8B) highlights the distribution density and hotspot areas of keywords, calculated based on keyword occurrence frequency. Darker colors indicate higher frequency, representing research hotspots. The yellow areas are the most core and active research foci in the current field, including tissue engineering, angiogenesis, hydrogel, microparticle, etc., indicating that oxygen-releasing materials focusing on hydrogels and microparticles to promote vascularization and bone regeneration are the main current research directions. Based on the visualization map and the above analysis, it can be seen that the red cluster primarily revolves around tissue engineering and is closely connected with keywords such as scaffolds, 3D printing and biomaterials, indicating extensive research on biomaterials and scaffolds in the field of tissue engineering. Notably, oxygen as a key node also belongs to this category, further highlighting the indispensable biological role of oxygen in tissue construction, especially the important function of oxygen-releasing materials in alleviating tissue hypoxia and promoting cell survival and functional expression. The blue cluster focuses on stem cells, including differentiation, proliferation and mesenchymal stem cells. It has clear associations with the previous two clusters, indicating that oxygen levels influence stem cell behavior, which is crucial for tissue regeneration. Other clusters represent more specific research directions. For example, drug delivery and controlled release indicate current research on using carriers such as microspheres or hydrogels to achieve precise oxygen delivery, while wound healing illustrates the diversity and importance of therapeutic strategies, requiring the design of different oxygen-releasing scaffolds for various tissue injuries (e.g. nerve, bone, skin or heart). In summary, this indicates that materials (scaffolds and carriers), biology (cells and vasculature) and scaffold fabrication technologies (3D printing, controlled release) together form the research pillars of oxygen-releasing scaffolds in tissue engineering. Furthermore, oxygen as a fundamental node traverses all clusters, also underscoring the importance of oxygen in the field of tissue engineering.

Currently, the core challenge for oxygen-releasing materials in tissue engineering lies in achieving efficient and controlled oxygen delivery. Constructing systems based on microsphere carriers and hydrogels has become a key technological approach to address this challenge. Current research has gradually formed a complete system encompassing material design, oxygen release, cellular and vascular support and ultimately tissue regeneration. Currently, intelligent responsive biomaterials (e.g. responsive to temperature, light, US) and material fabrication technologies such as 3D-printed scaffolds, hydrogel preparation and nanotechnology provide technical support for advancing the development of next-generation bionic oxygen delivery systems. Looking ahead, the development trend of oxygen-releasing materials will focus on creating intelligent oxygen carriers capable of responding to endogenous biological signals, achieving spatiotemporally precise on-demand oxygen release that better aligns with the characteristics of treating different tissue injuries, thereby enabling more accurate oxygen release at the required time and location. Simultaneously, integrating artificial intelligence technology, gene therapy and implantable sensing electronic devices will lead to the construction of multifunctional integrated tissue engineering scaffolds. Furthermore, such technologies will be increasingly applied to complex scenarios requiring substantial oxygen support, such as nerve regeneration, myocardial repair and large organ construction. In summary, oxygen delivery strategies have become a key research direction in tissue engineering. Relying on material innovation and multidisciplinary collaboration, they aim to fundamentally address the oxygen supply issue during tissue regeneration, holding significant scientific value and broad application prospects.

## Conclusion and prospect

This article offers a systematic overview of the advancements in oxygen-releasing scaffolds for applications in tissue repair and regeneration. It comprehensively discusses the types of oxygen carrier materials, scaffold construction strategies, controlled oxygen release techniques, ROS scavenging methods and their applications across various tissue engineering fields. By continuously improving the local hypoxic microenvironment, oxygen-releasing scaffolds have shown considerable promise in fields including bone, skin, nerve and cardiovascular repair, as well as in tumor therapy. Current research has established a complete system ranging from basic material design to functional applications. Studies on sustained-release oxygen scaffolds are relatively extensive, and currently, intelligent responsive oxygen-releasing materials have become a research hotspot. This review aims to offer references and a foundation for the continued advancement of tissue engineering oxygen-releasing scaffolds and their practical implementation in tissue regeneration.

As an innovative material in tissue engineering and regenerative medicine, oxygen-releasing scaffolds provide an effective strategy for addressing postimplantation cell hypoxia by continuously regulating the local oxygen microenvironment. Current research has developed various functionalized scaffolds, including chemical oxygen-releasing scaffolds based on peroxides such as CPO, MPO and H_2_O_2_, physical oxygen-carrying materials based on Hb and PFCs; and intelligent responsive oxygen-releasing systems triggered by photothermal, pH or US stimuli. Through technologies such as 3D printing, microsphere encapsulation and hydrogel integration, these scaffolds enable controlled oxygen release and have demonstrated significant potential in bone, cartilage, cardiovascular and neural regeneration. Furthermore, interdisciplinary strategies such as photosynthetic algae systems and cell membrane-anchored oxygen carriers further expand the diversity of oxygen release mechanisms.

However, current research on tissue engineering oxygen-releasing scaffolds still faces several key challenges. Regarding the regulation of oxygen release kinetics, although various intelligent responsive oxygen release systems have been developed for on-demand oxygen release, initial burst release and subsequent decay remain common issues, which complicates the matching of the long-term, dynamic oxygen needs during tissue regeneration. Future efforts need to combine material modification and microstructural design to further improve the precision and environmental responsiveness of oxygen release behavior. In terms of functionality, current oxygen-releasing scaffolds have relatively single functions, with extensive research focused primarily on their oxygen-release capabilities [149, 150, 188]. Future work should integrate oxygen release synergistically with bioactive factors to construct multifunctional and integrated scaffolds, addressing the multiple demands of complex tissue microenvironments and improving therapeutic efficiency. For instance, incorporating the synergistic release of Mg^2+^ and oxygen in bone tissue engineering enhances both vascularization and osteogenic differentiation [189, 190], while combining neurotrophic factors in neural regeneration can help guide axonal regeneration and precise connection [191, 192]. Furthermore, electrical stimulation has been proven to promote tissue repair such as neural regeneration [193–196], yet the combination of electrical stimulation systems with oxygen release is relatively rare. In the future, by integrating conductive materials with oxygen-releasing materials—for instance, combining oxygen-releasing materials with conductive biomaterials or piezoelectric biomaterials—thermal effects, deformation or electrical stimulation induced by applied electric fields or external stimuli could be utilized to enhance oxygen carrier diffusion, thereby controlling the oxygen release rate and achieving synergistic effects between electrical stimulation and precise oxygen release. To achieve real-time regulation of oxygen generation as a new, precise and controllable strategy, matching the timing of oxygen release with the regeneration requirements of different tissues for more efficient oxygen supply. In the future, by designing carriers capable of responding to endogenous signals specific to the hypoxic injury microenvironment—such as pH, temperature and ROS levels—or by utilizing external stimuli like light, sound or magnetism for remote precise control, on-demand oxygen release can be realized. Advanced manufacturing technologies such as high-precision 3D printing, microfluidics and electrospinning can be employed to customize personalized oxygen-releasing scaffolds with biomimetic microstructures and controllable release kinetics. Although current oxygen-releasing scaffolds still face multiple challenges, including release stability and compatibility with complex microenvironments, the future should focus on integrating cutting-edge technologies from materials science, bioengineering, clinical medicine and other disciplines to develop next-generation oxygen-releasing scaffolds capable of dynamic sensing, precise response and synergistic interaction with human tissue physiological processes, thereby advancing their clinical applications. However, during clinical translation and large-scale manufacturing, oxygen-releasing scaffolds still face significant challenges, including insufficient stability of the preparation process, high batch-to-batch variability of oxygen carrier materials, lack of quality control standards, incomplete storage protocols and cost and supply chain issues. Specifically, techniques commonly used in research, such as 3D printing, microsphere encapsulation and electrospinning, are prone to batch-to-batch variations during scale-up production, affecting the consistency of oxygen release kinetics. Chemical oxygen-releasing materials such as CPO, MPO and H_2_O_2_ are sensitive to temperature and humidity, while physical oxygen-carrying materials like Hb and PFCs are susceptible to denaturation and inactivation during large-scale processing. Furthermore, there is a lack of online monitoring methods for oxygen release rates and unified quality control standards. Moreover, the high preparation cost of some oxygen carriers further restricts the feasibility of clinical translation. In the future, large-scale manufacturing should adopt more stable preparation methods such as microfluidics, establish monitoring systems for oxygen release capacity, optimize storage conditions and reduce preparation costs, thereby promoting the clinical translation and application of oxygen-releasing scaffolds in tissue regeneration.

## Supplementary Material

rbag096_Supplementary_Data

## Data Availability

The data that support the findings of this study are available from the corresponding author upon reasonable request.

## Supplementary data

All copyright permissions for figures reproduced or adapted from other sources are provided in the Supplementary Material. Supplementary data are available at *Regenerative Biomaterials* online.
