# Supplementary material for: Oxygen-releasing scaffolds in tissue engineering: design strategies, fabrication and regenerative applications
Source: Regen Biomater. 2026 May 13;13:rbag096. doi: 10.1093/rb/rbag096 (PMC13250950; doi:10.1093/rb/rbag096)

*Supplementary File*

Oxygen-Releasing Scaffolds in Tissue Engineering: Design Strategies, Fabrication, and Regenerative Applications

Yuqing Shang^1^，Lele Wang^1^，Huoyun Shen^1^，Mingzhu Jia^1^，Hongxia Gao^1^, Yaqiong Liu^1^, Yumin Yang^1^, Jian Yang^2*^, Linliang Wu^3*^, Guicai Li^1*^

^1^ Jiangsu Key Laboratory of Tissue Engineering and Neuroregeneration, Key Laboratory of Neuroregeneration of Ministry of Education, Co-innovation Center of Neuroregeneration, Nantong University, Nantong 226001, China

^2^ Department of Neurosurgery, People’s Hospital of Deyang City, Sichuan Clinical Research Center for Neurological Diseases, Deyang 618000, China

^3^Affiliated Rugao Hospital of Xinglin College, The People's Hospital of Rugao, Co-innovation Center of Neuroregeneration, Nantong University, 226500, Nantong, P. R. China.

Content：Copyright of all the figures cited from the published references.

FIG2:

1.


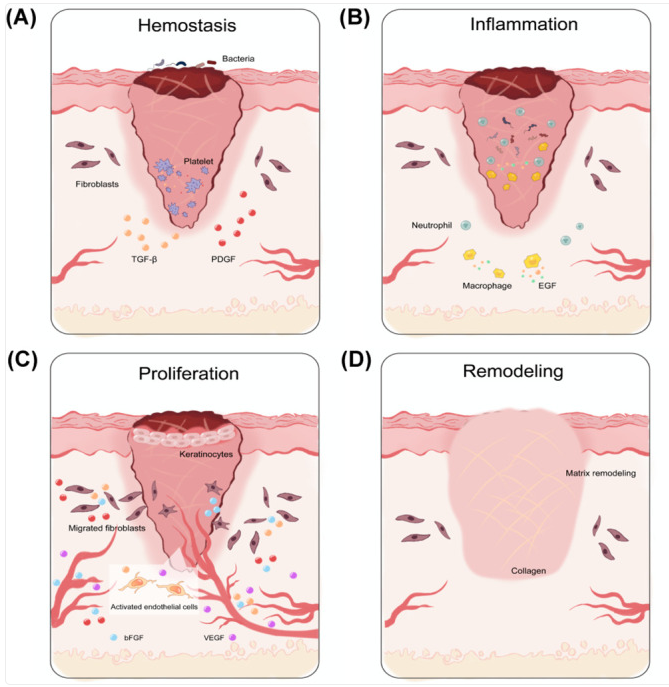


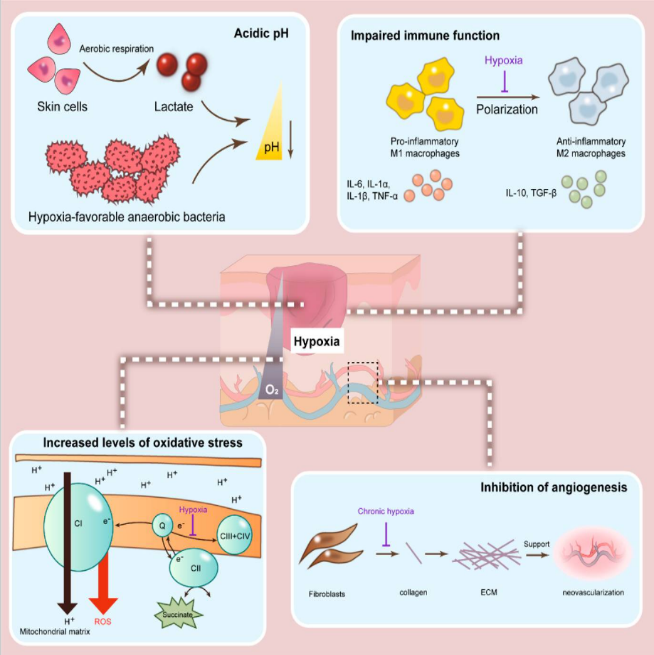


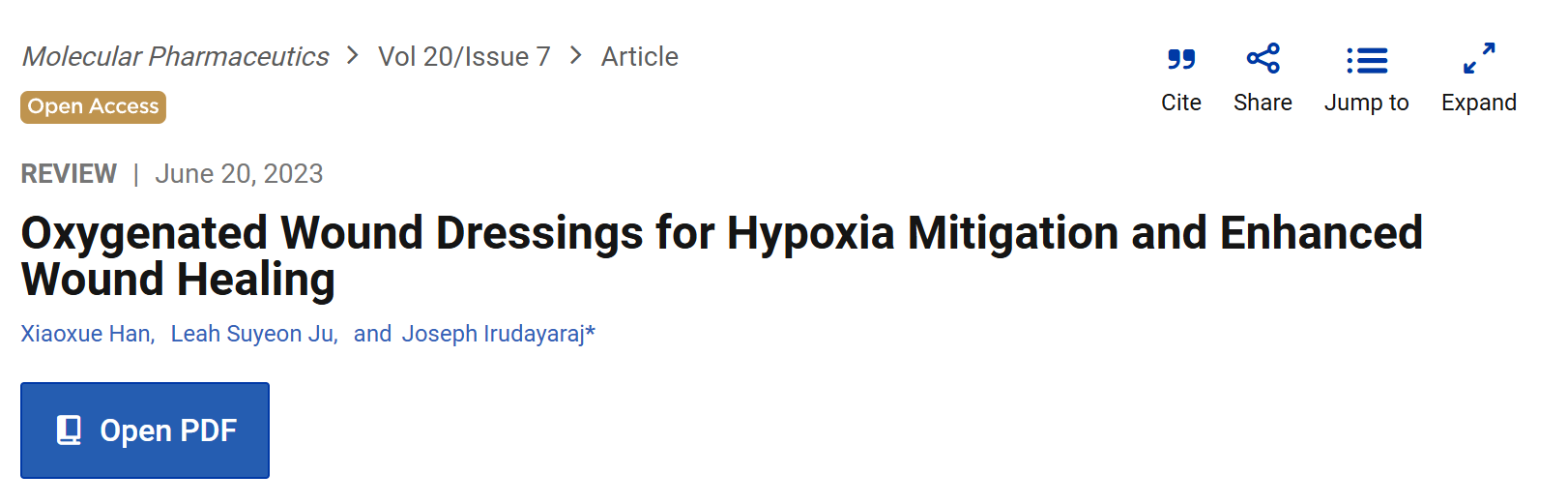


2.
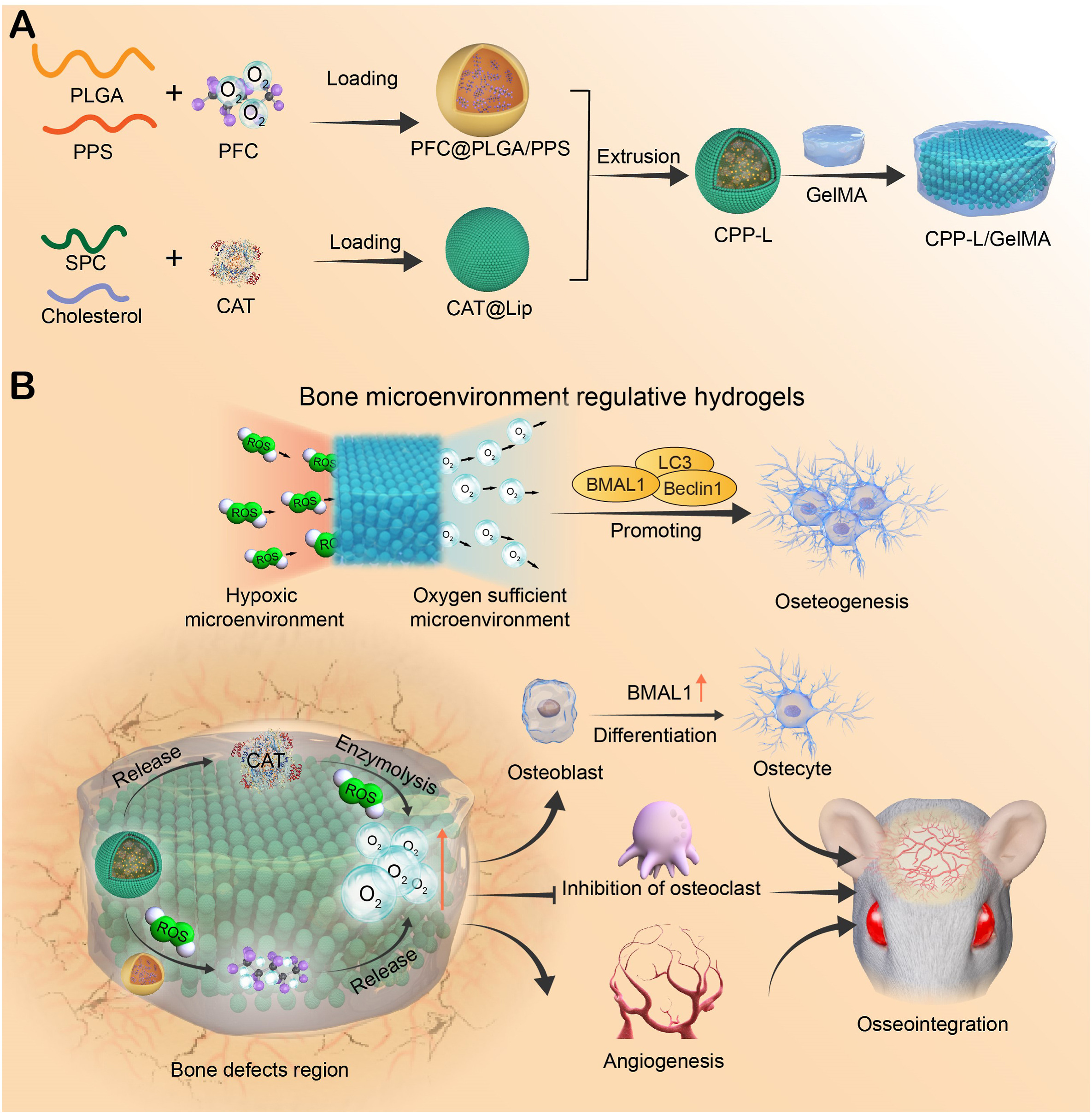


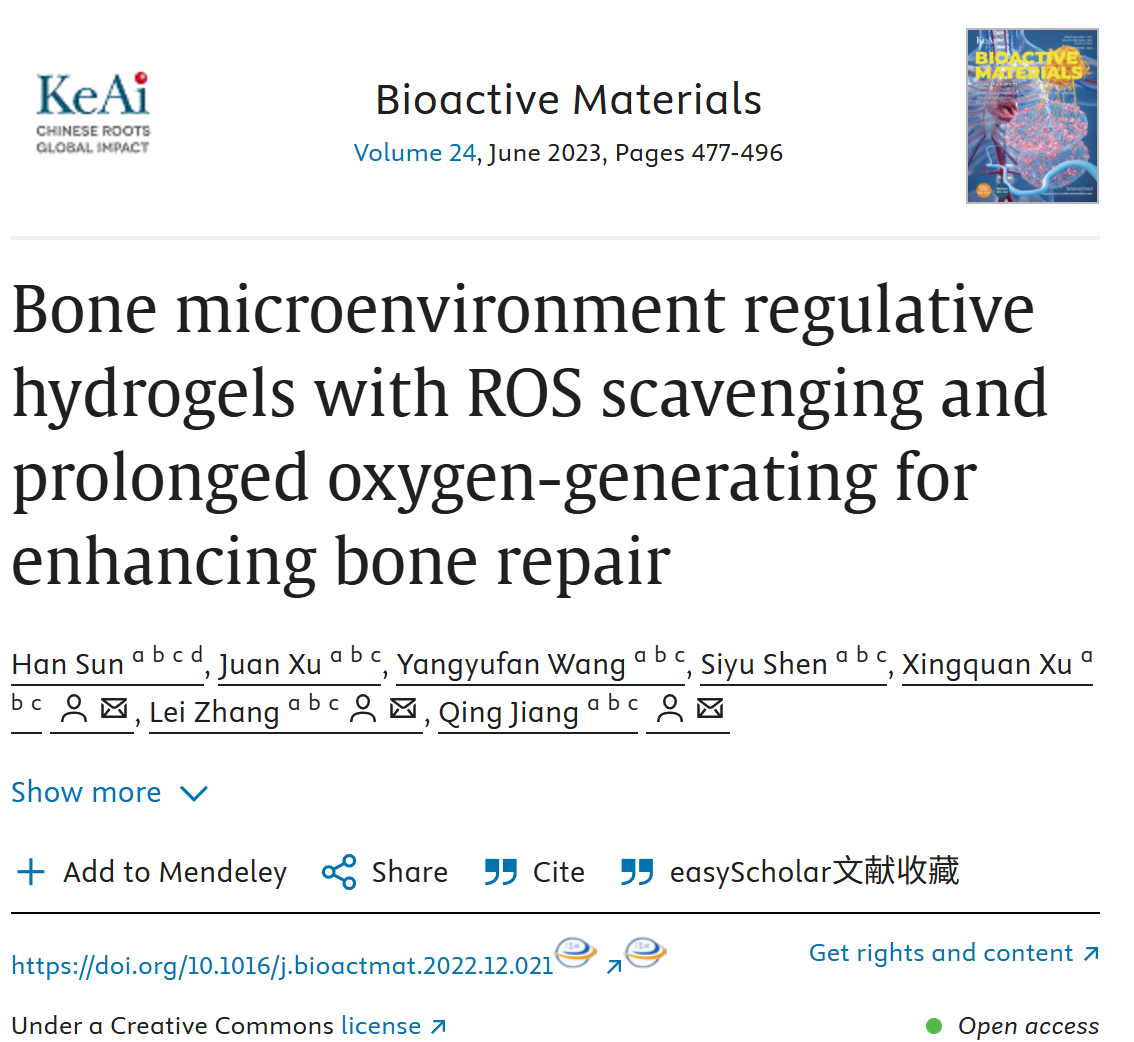


3.


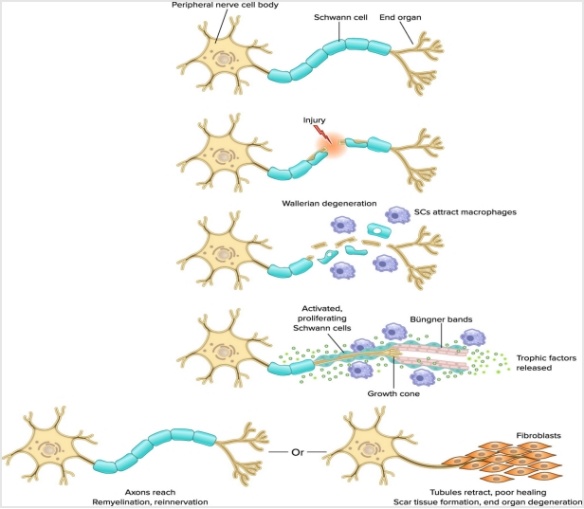

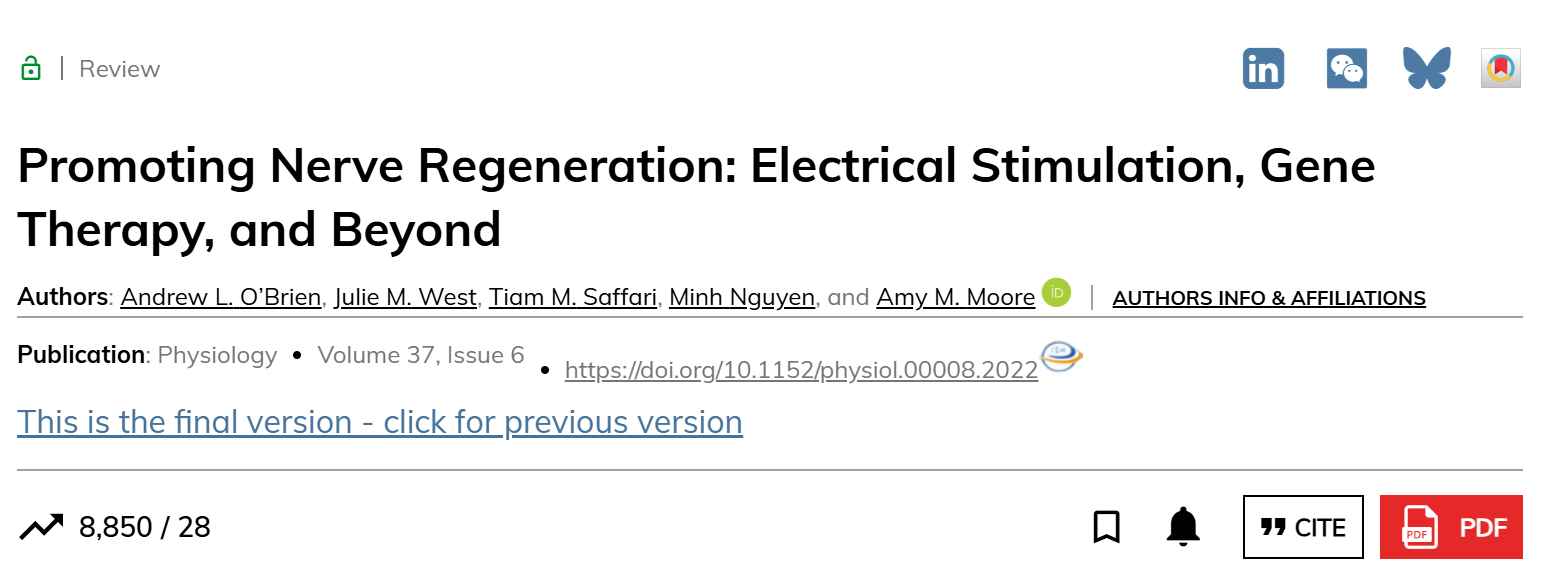


4.
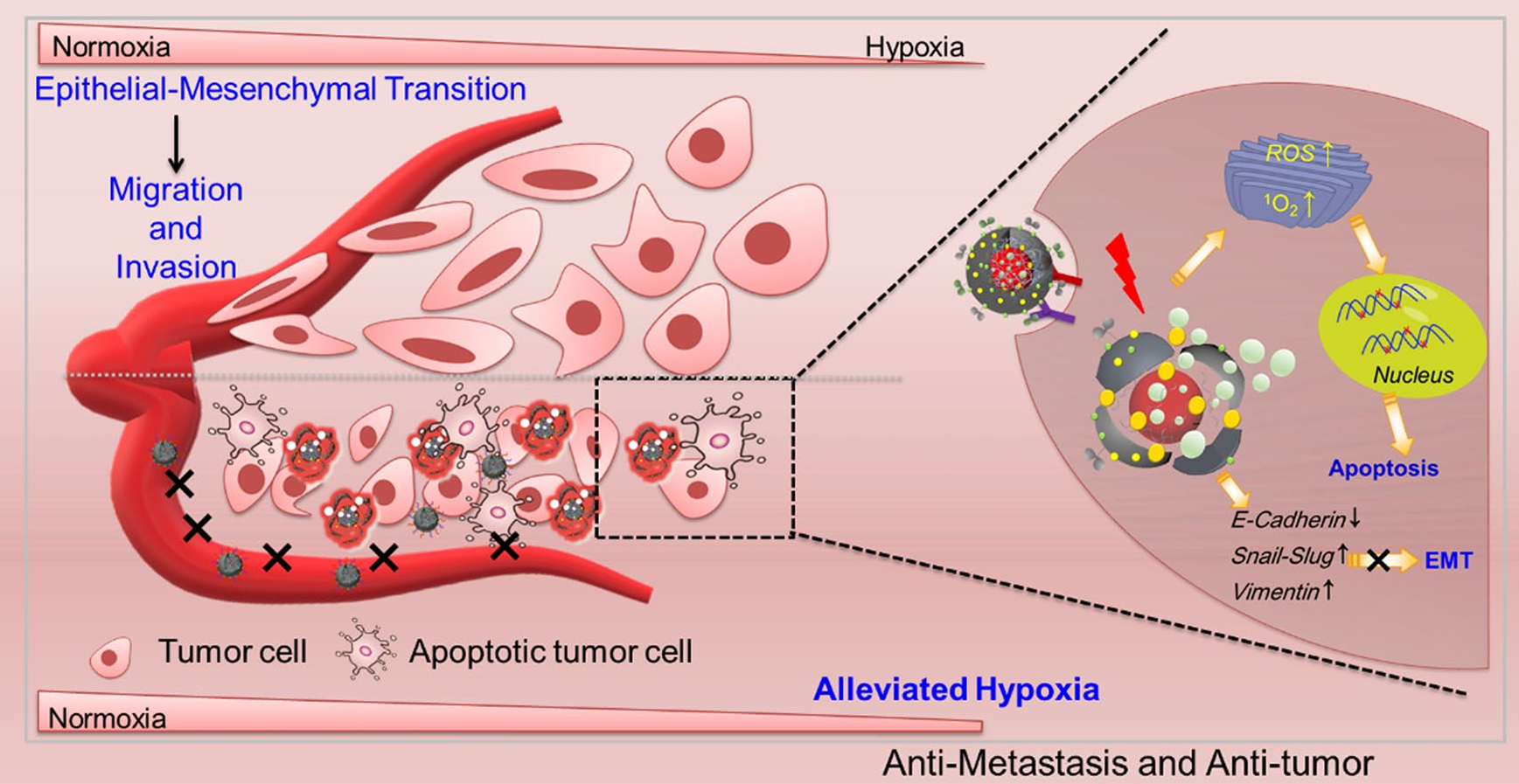

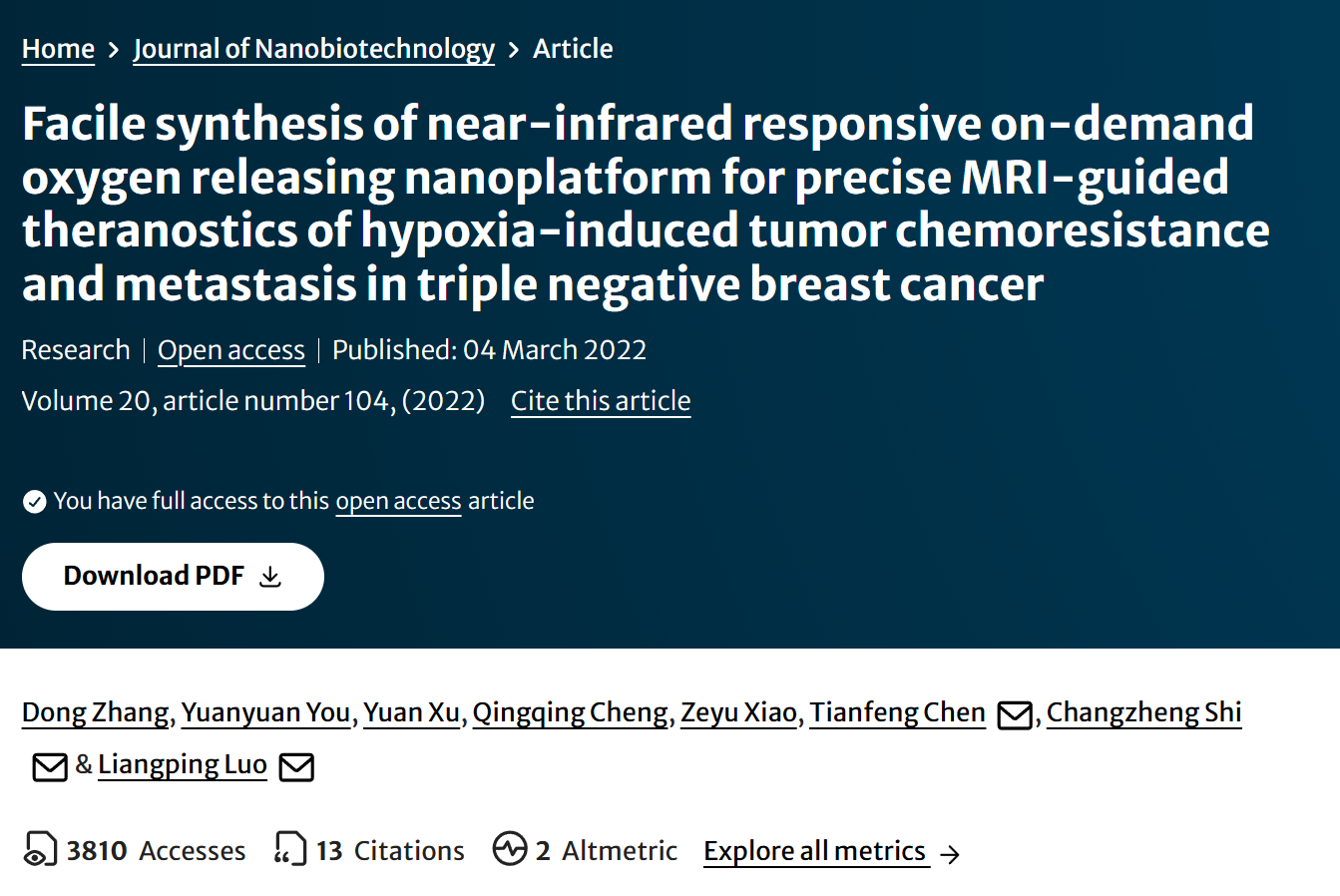


5.


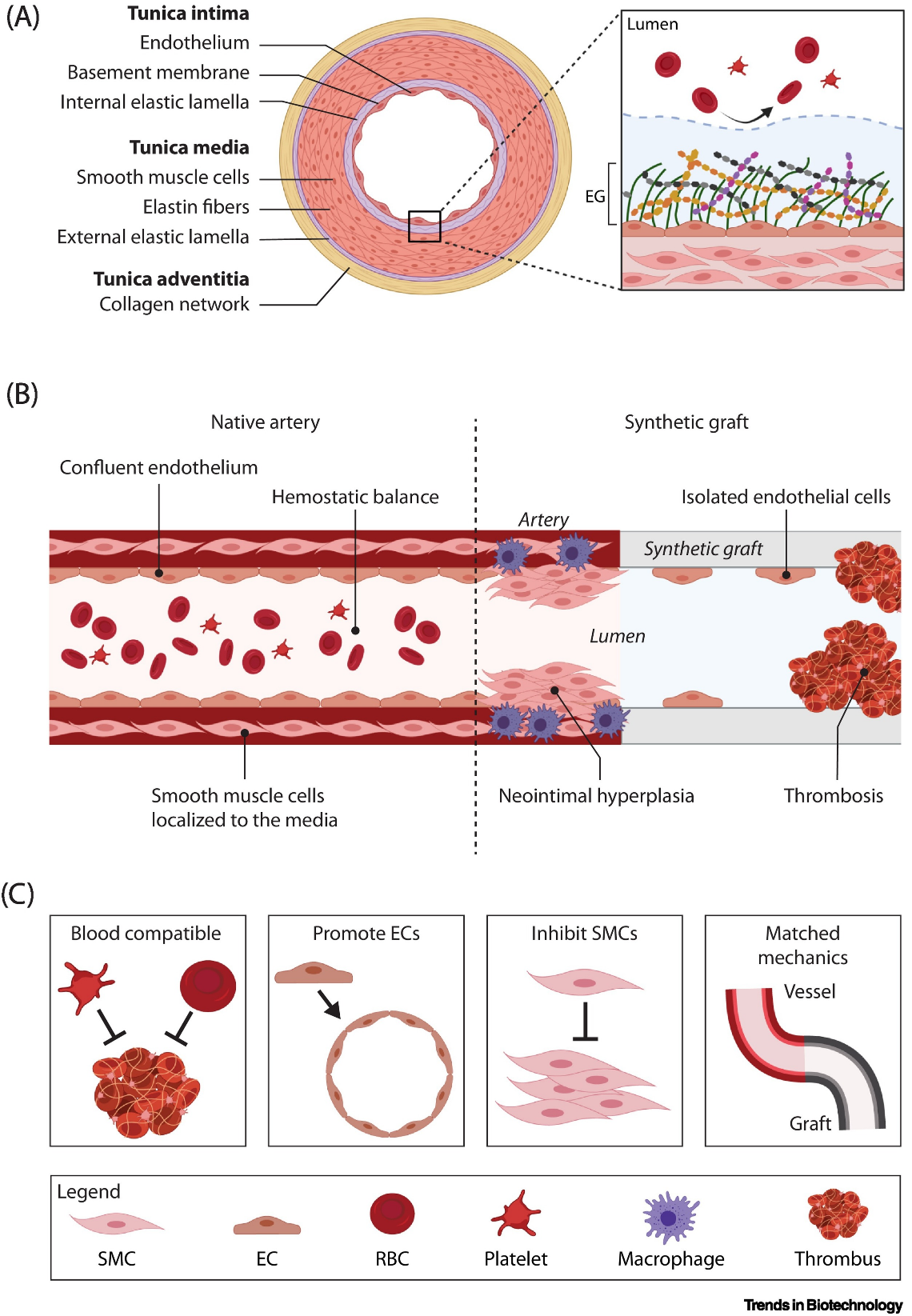

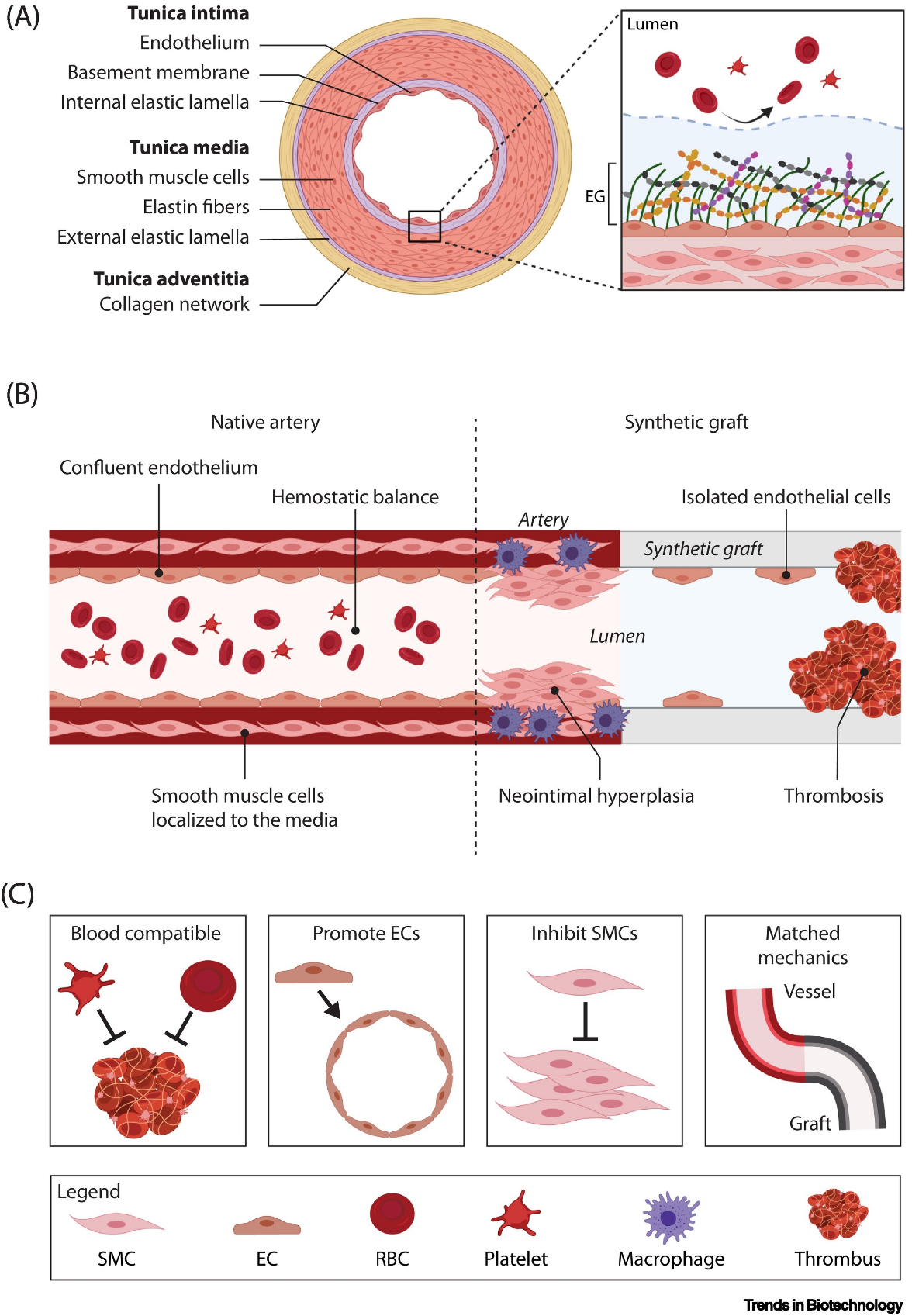

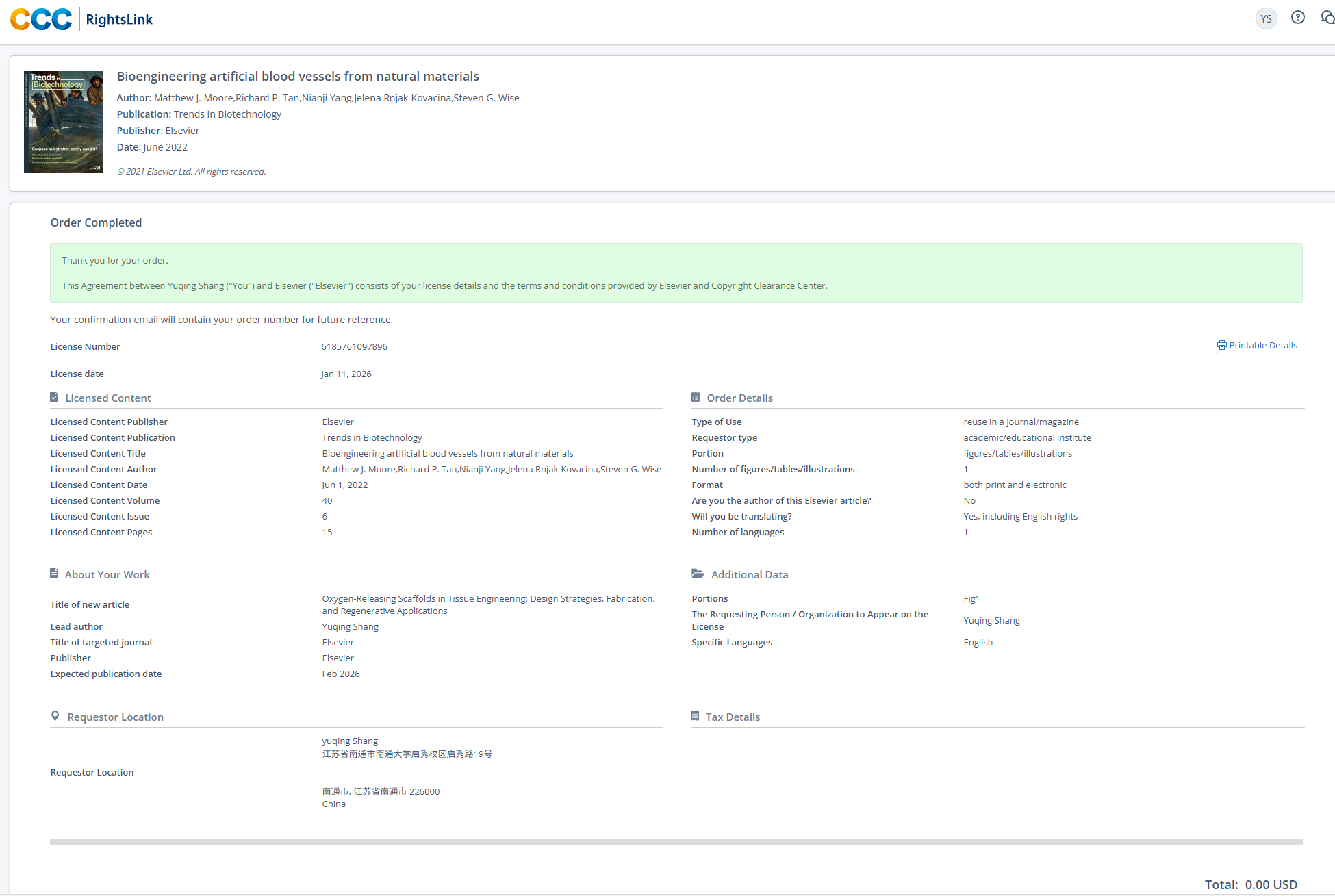


FIG3：

1.


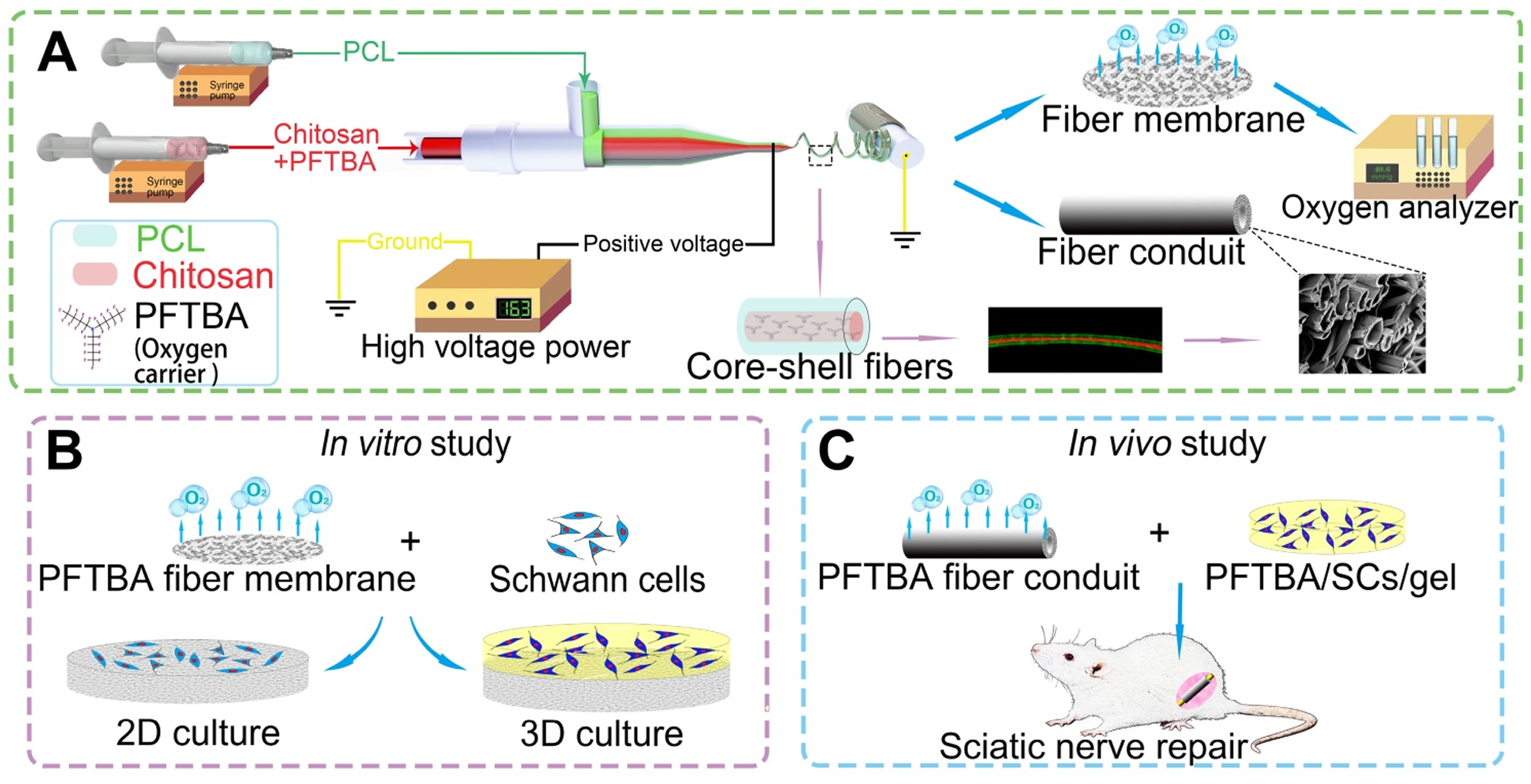


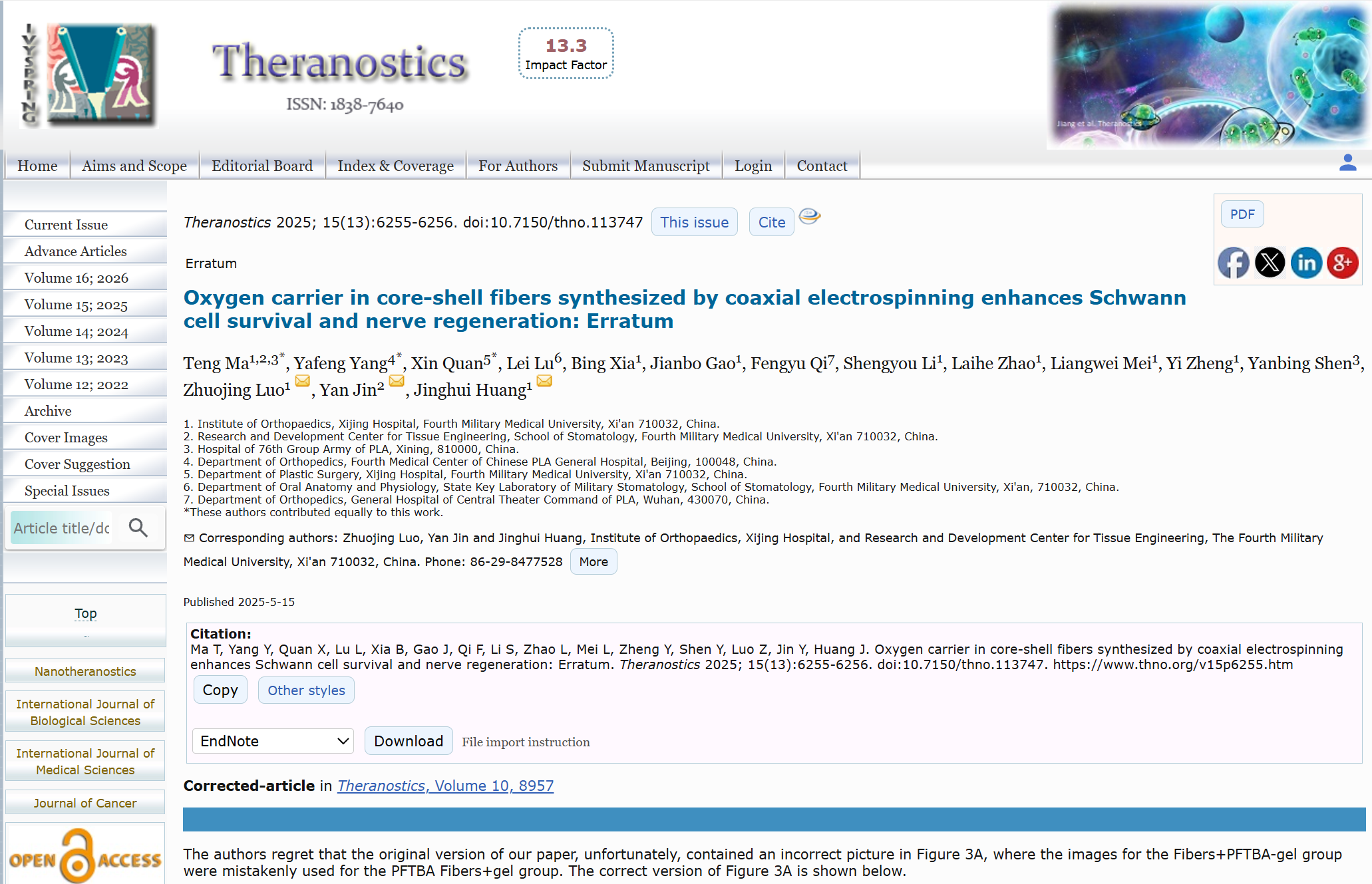


2.
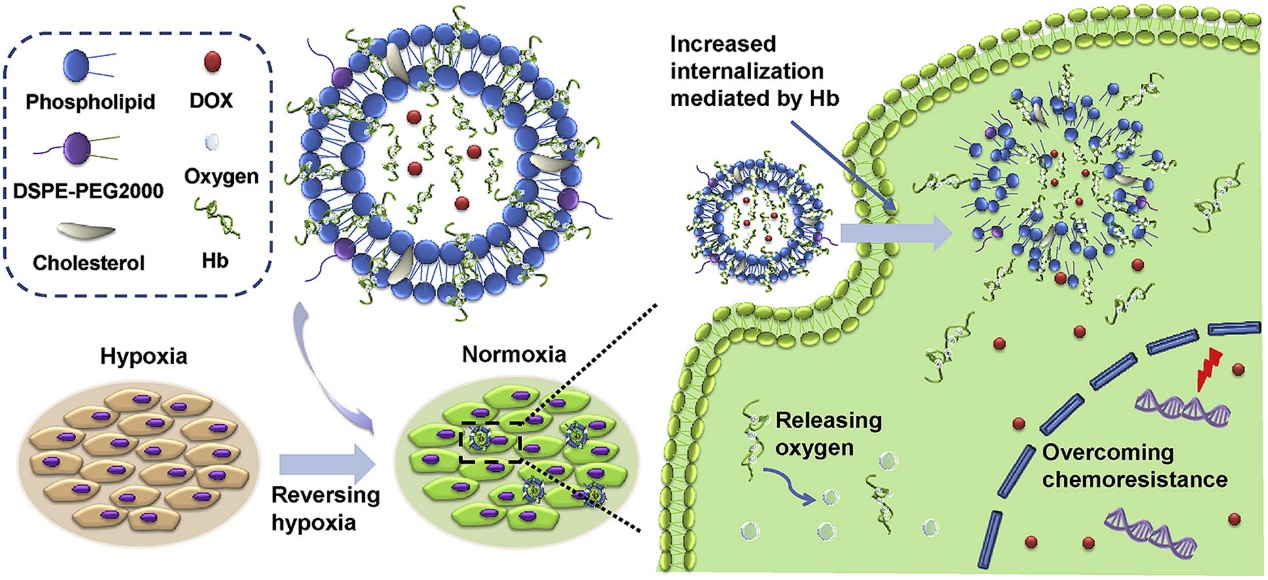


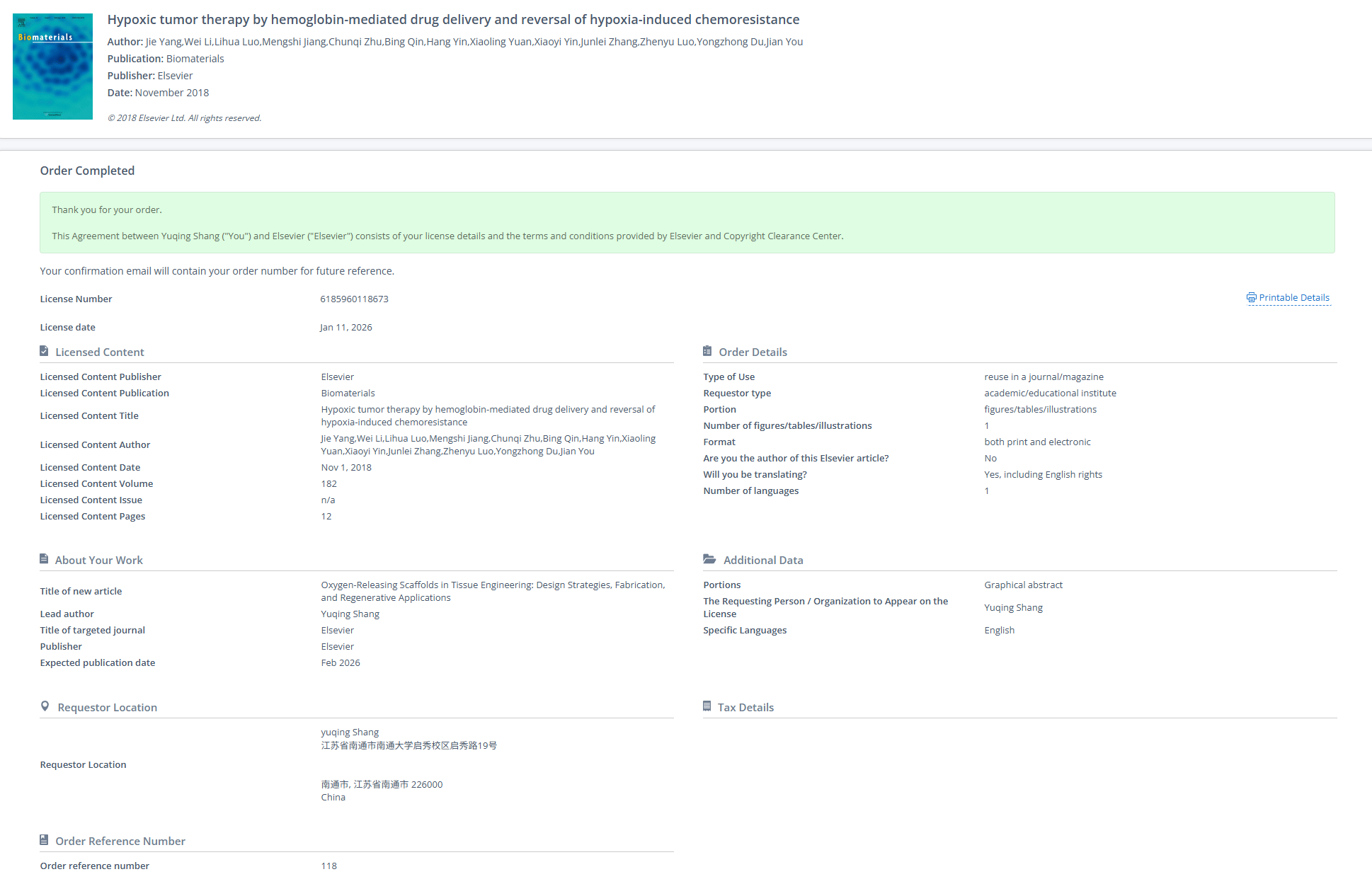


3.
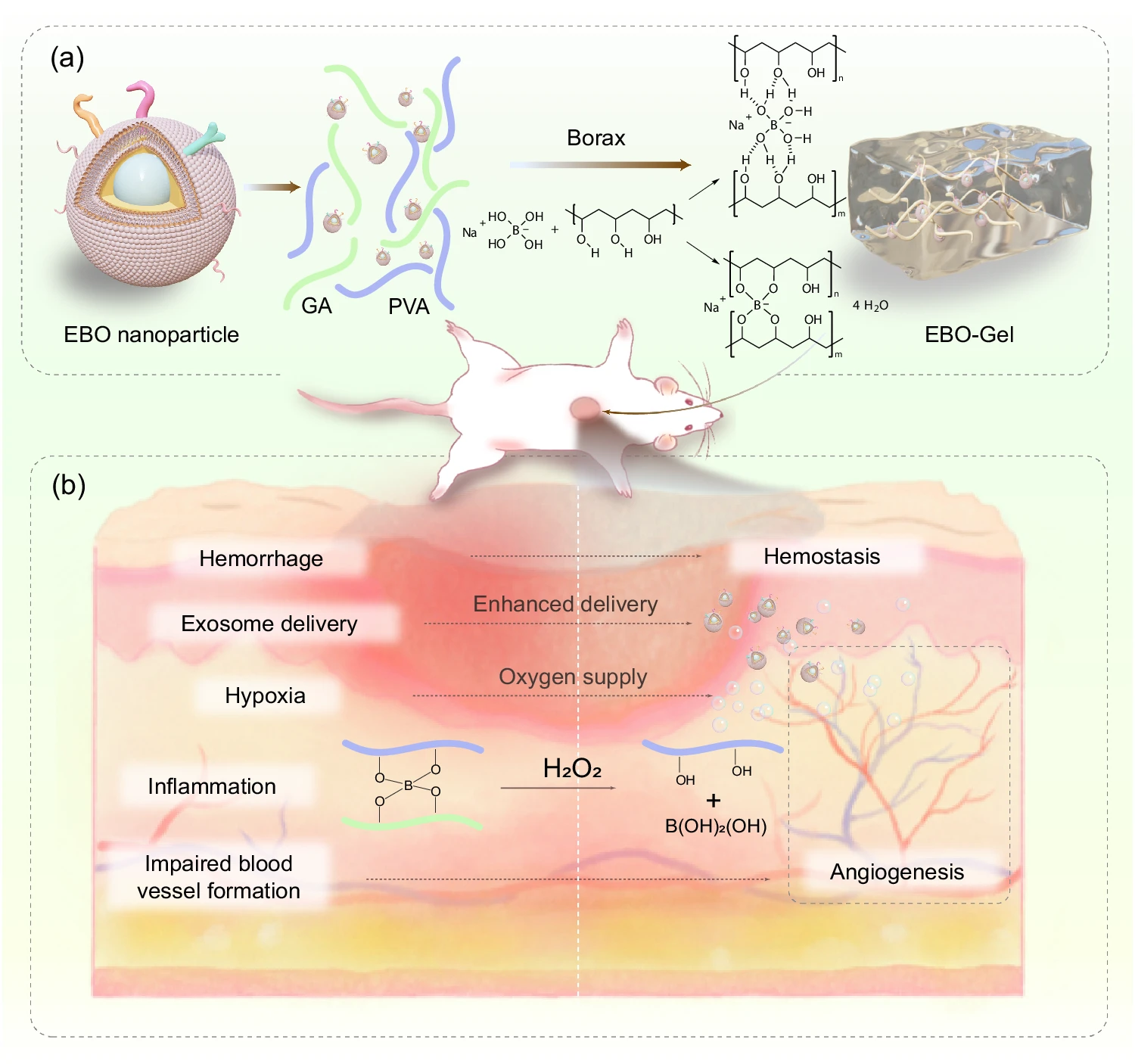


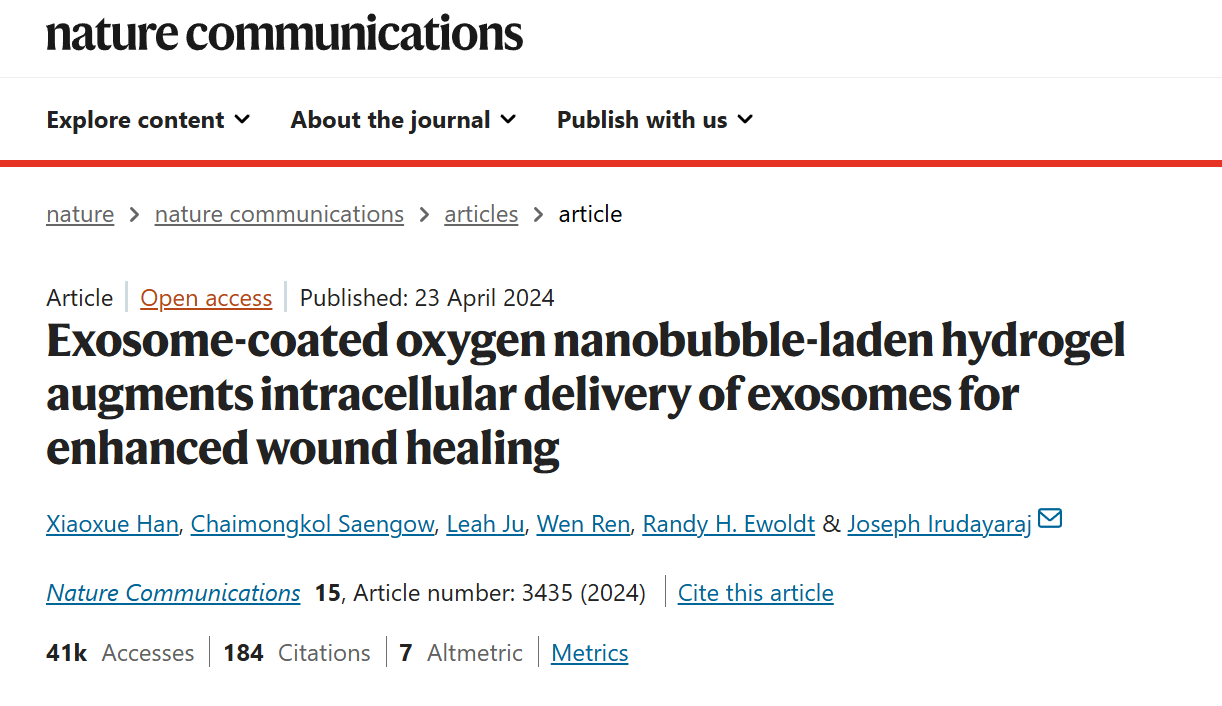


FIG4:


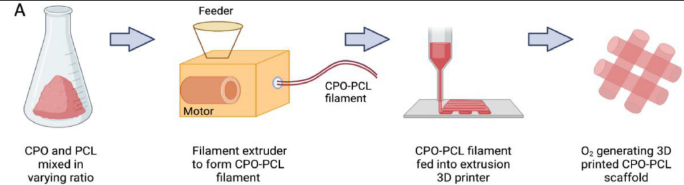

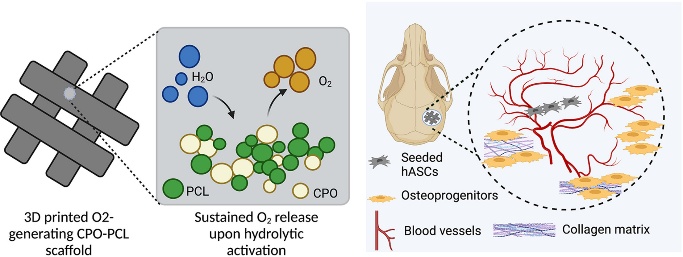


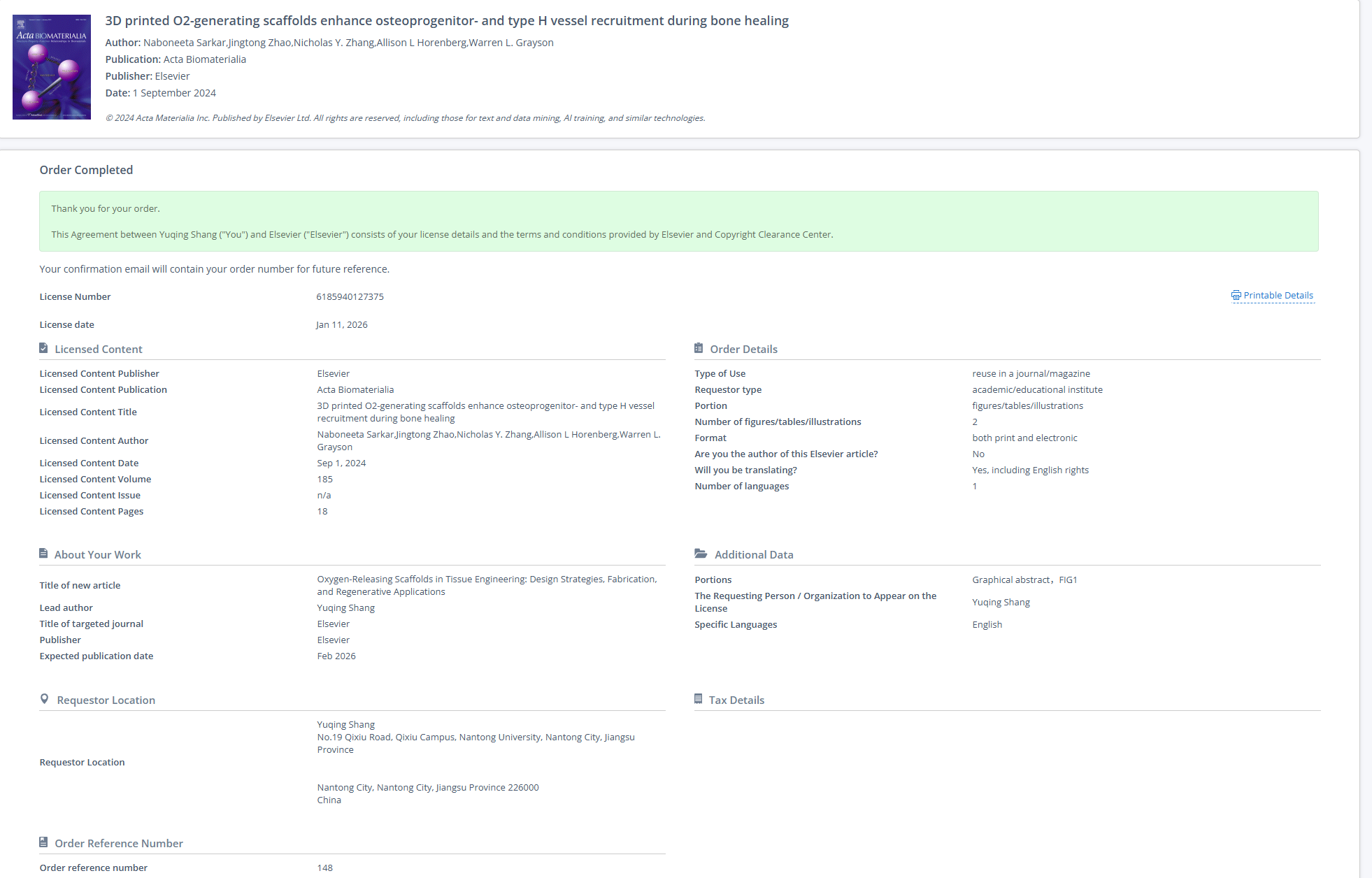


2.
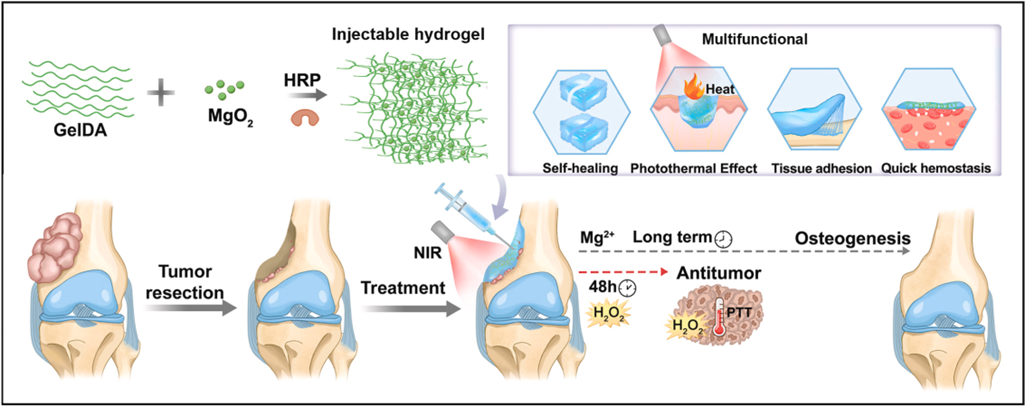


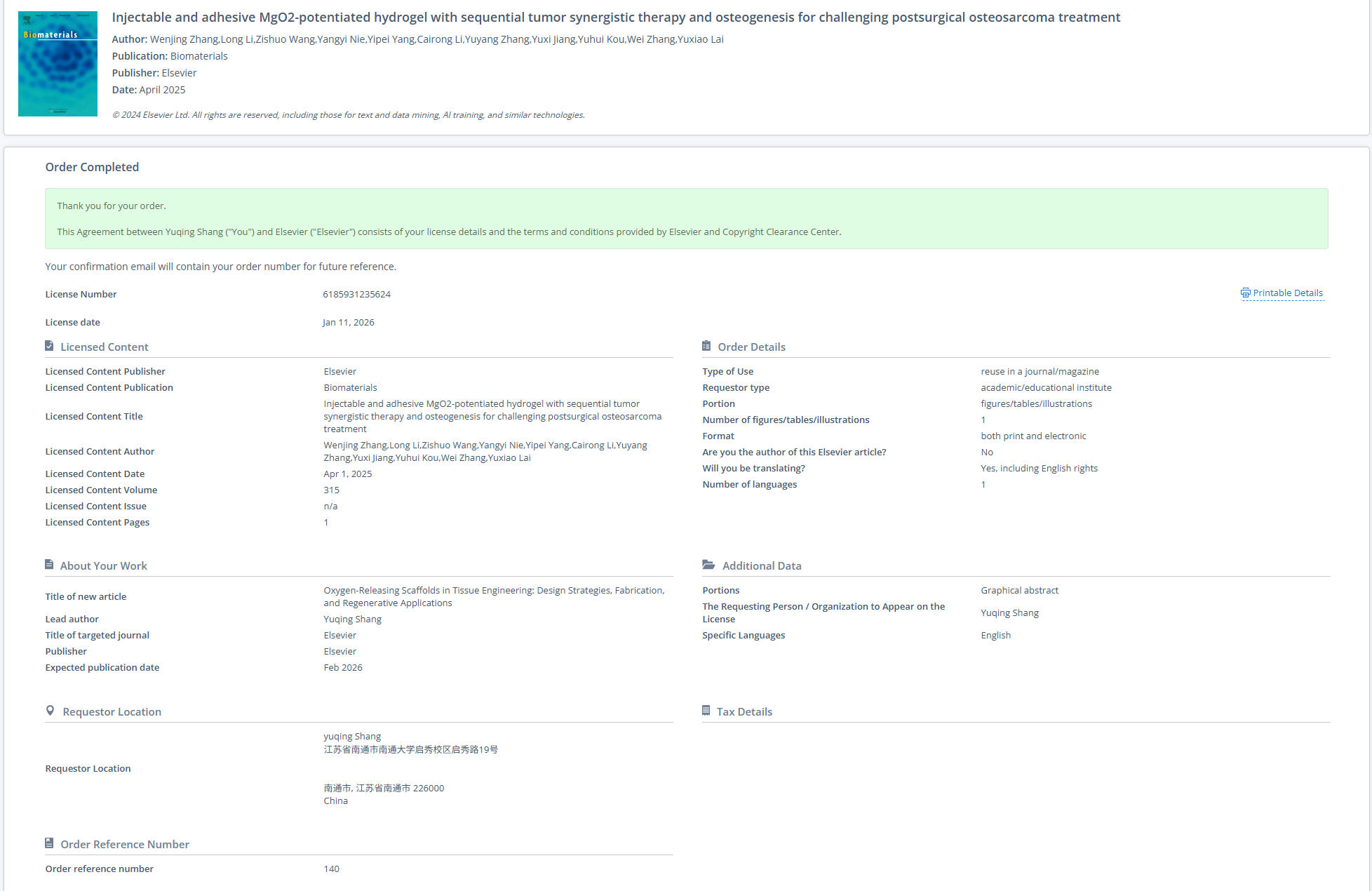


3.
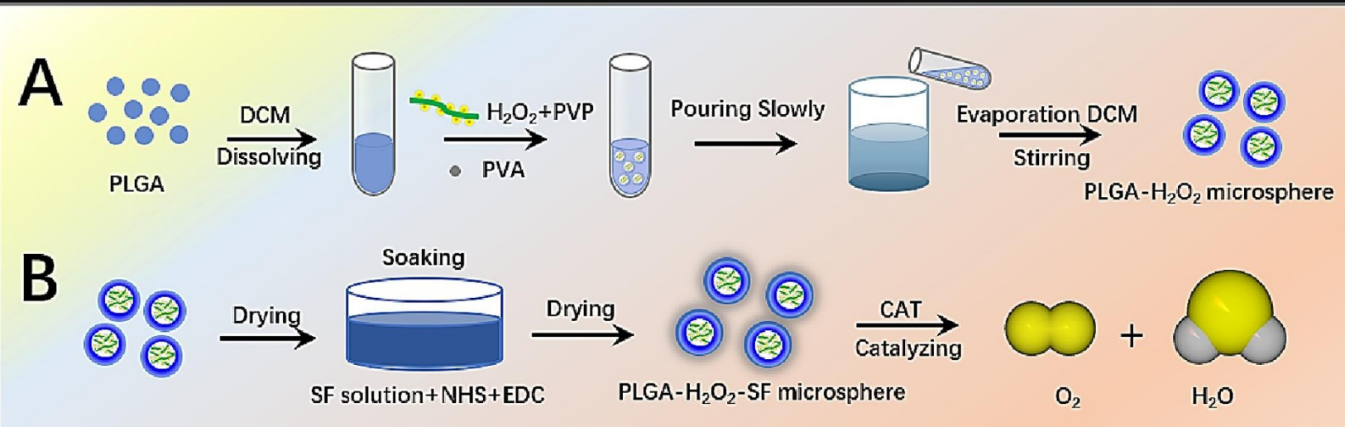

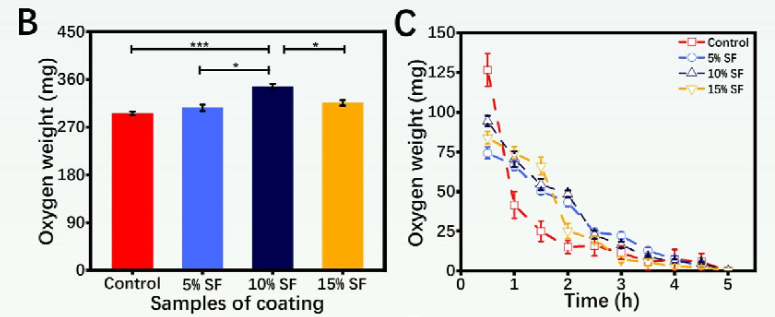


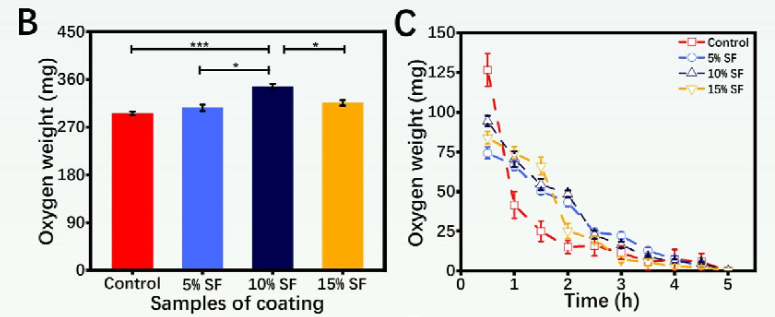


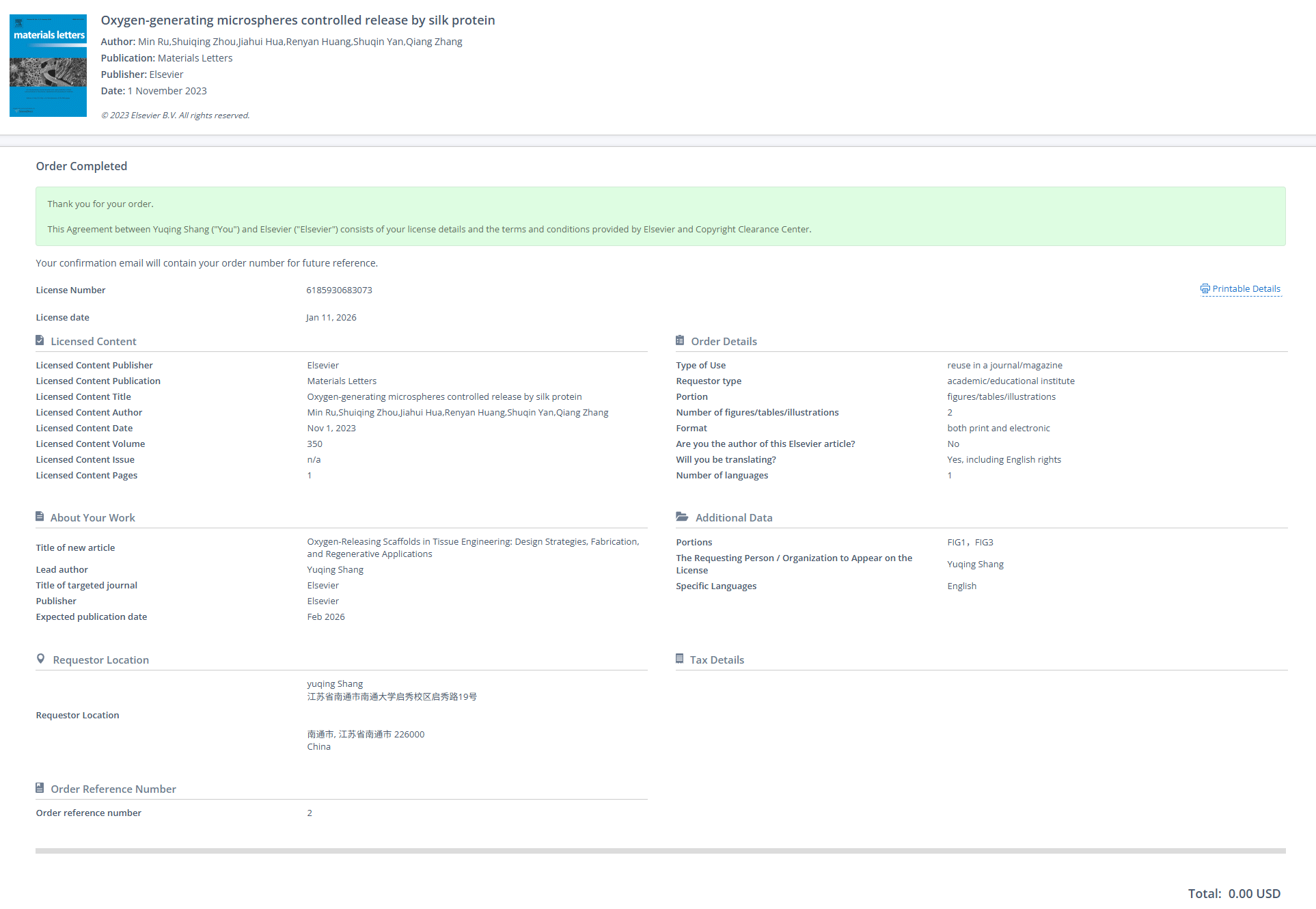


Fig5

1.


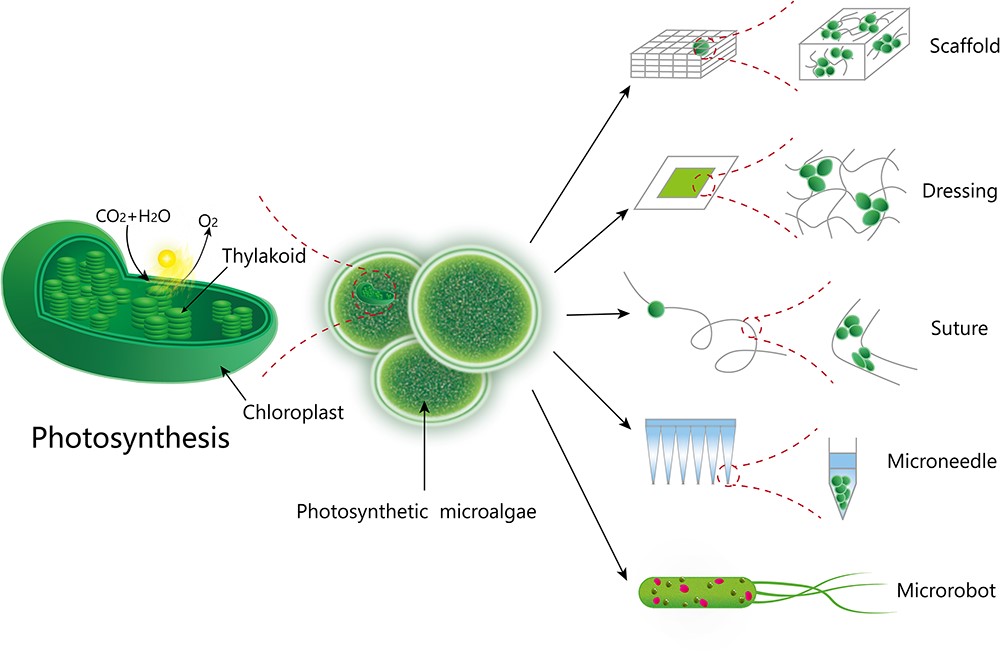


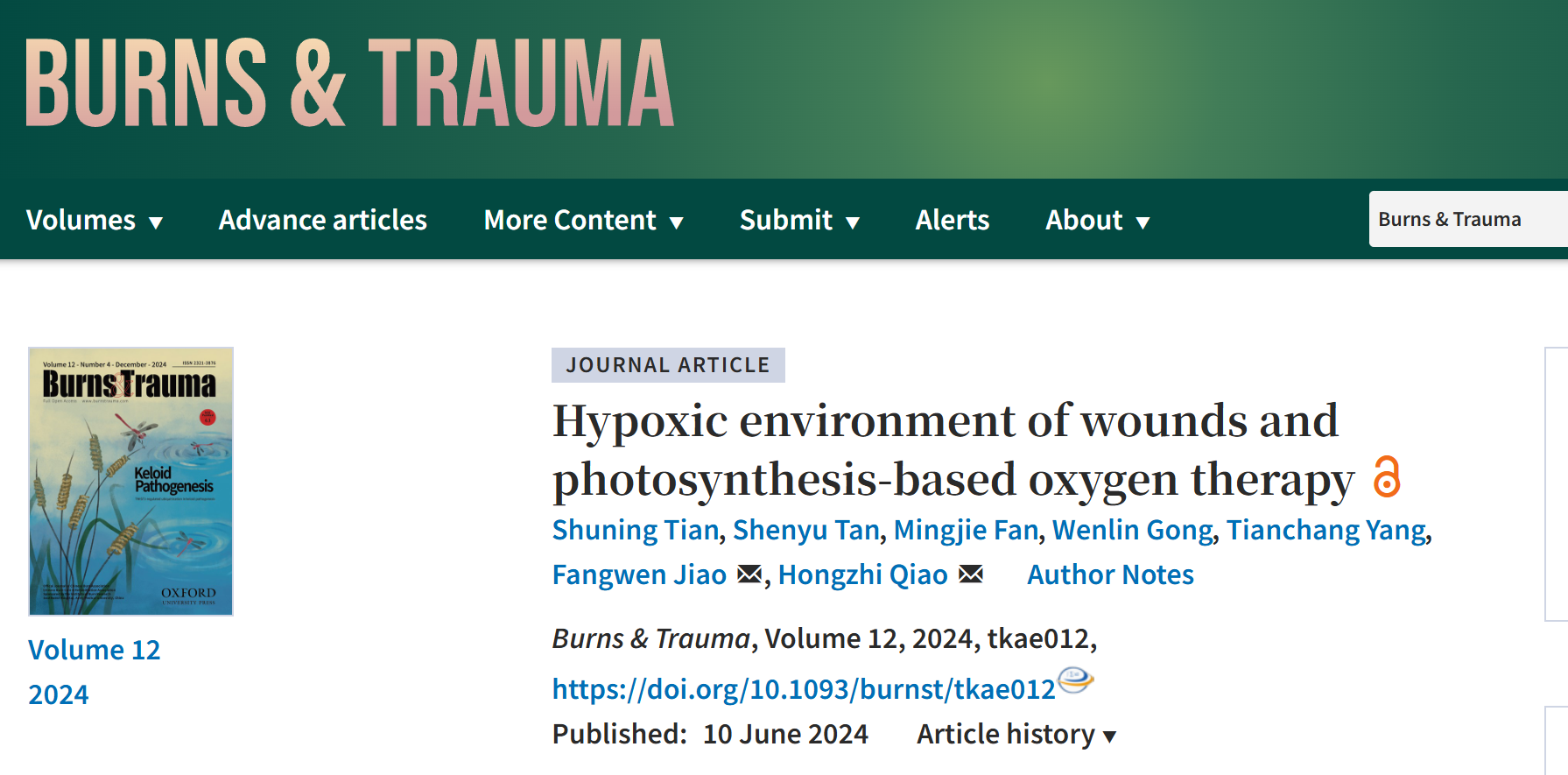


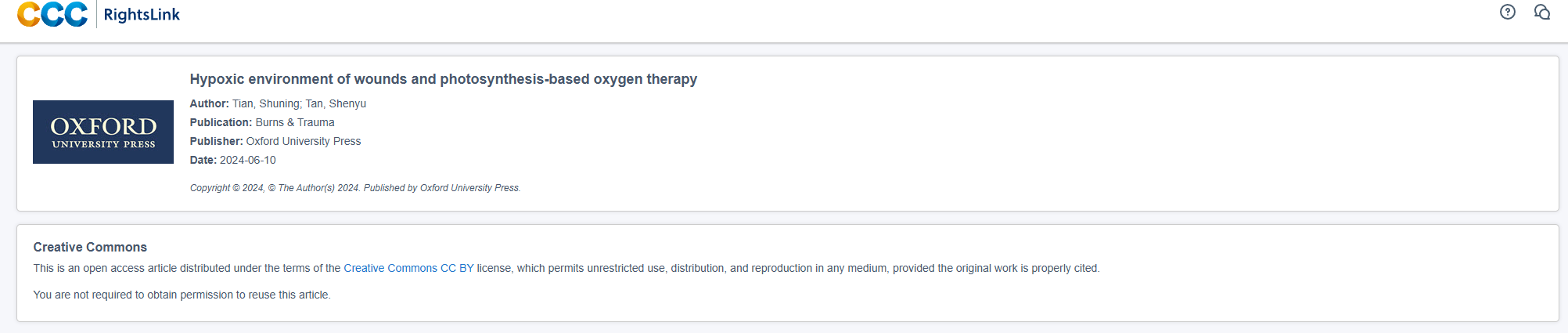


2.


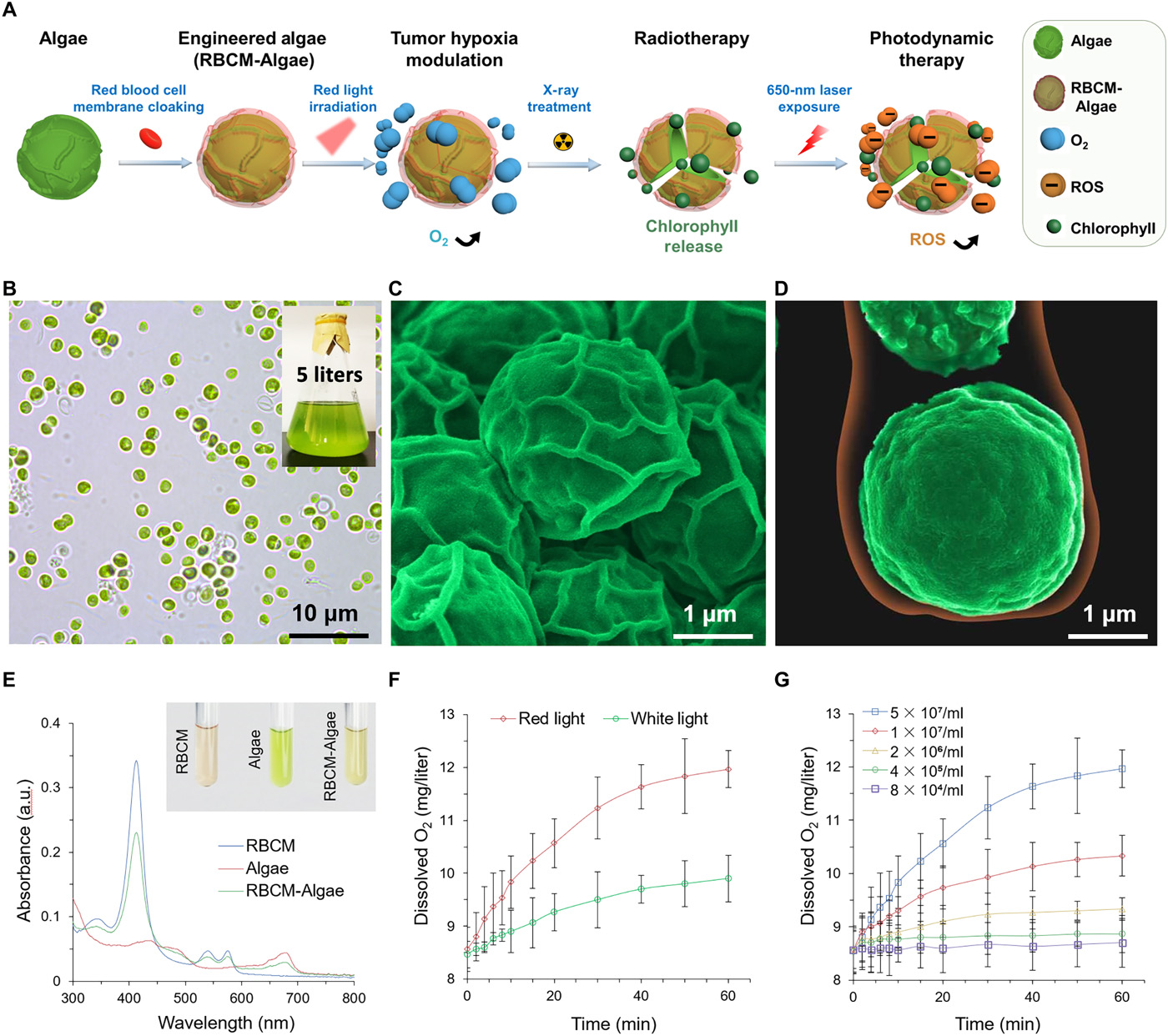


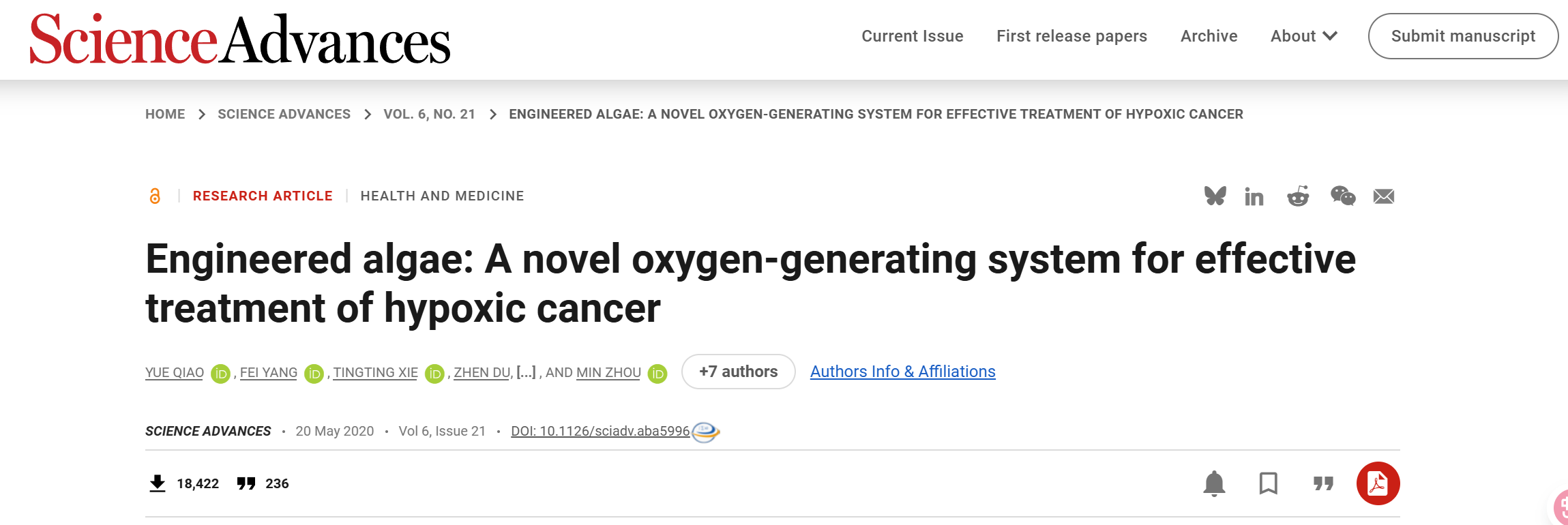


3.


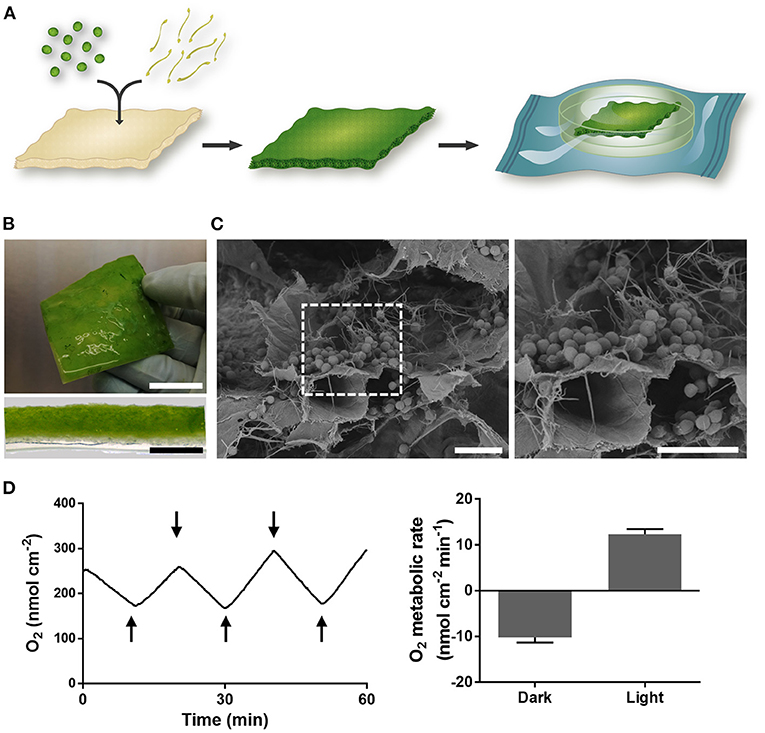


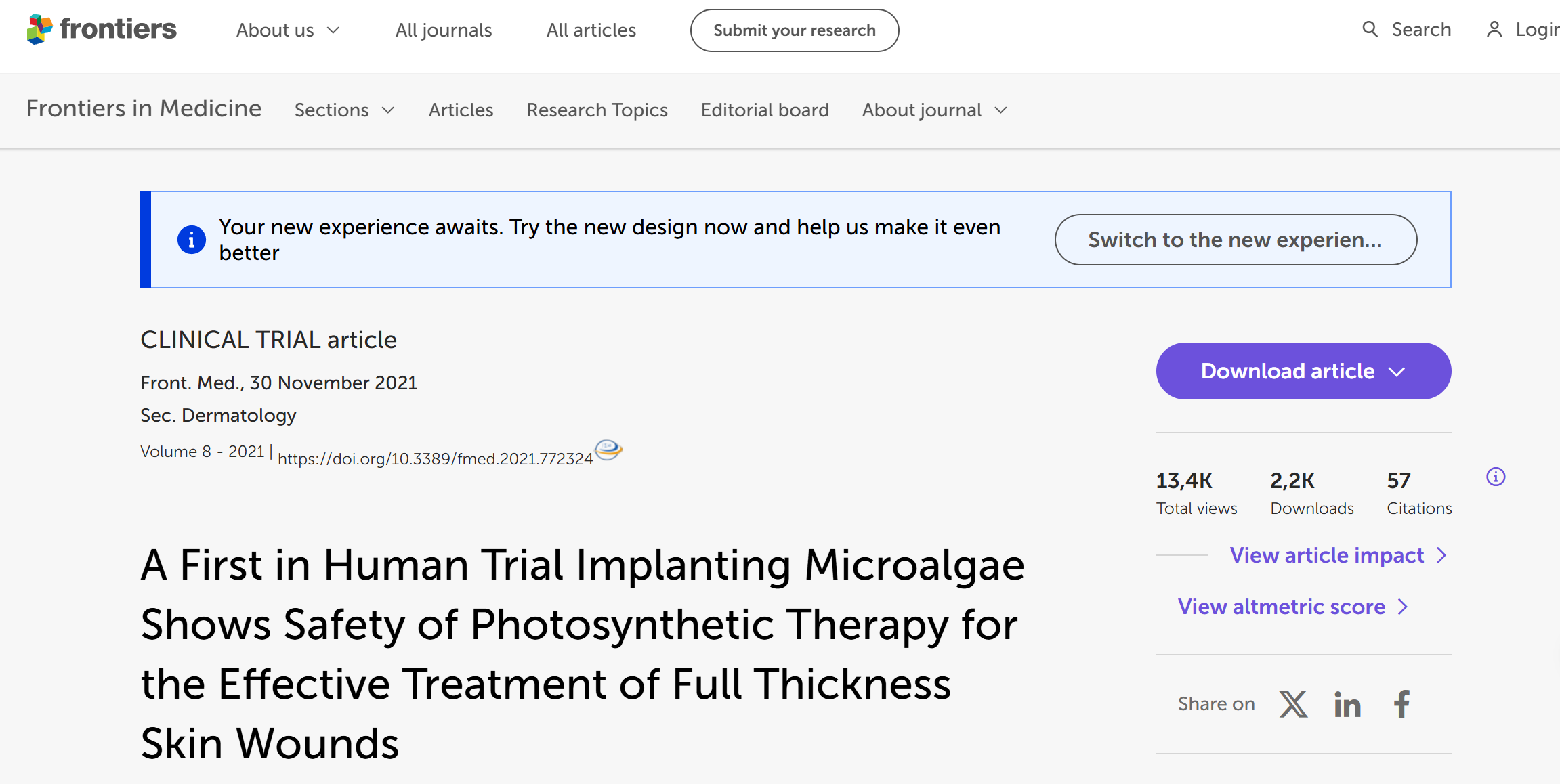


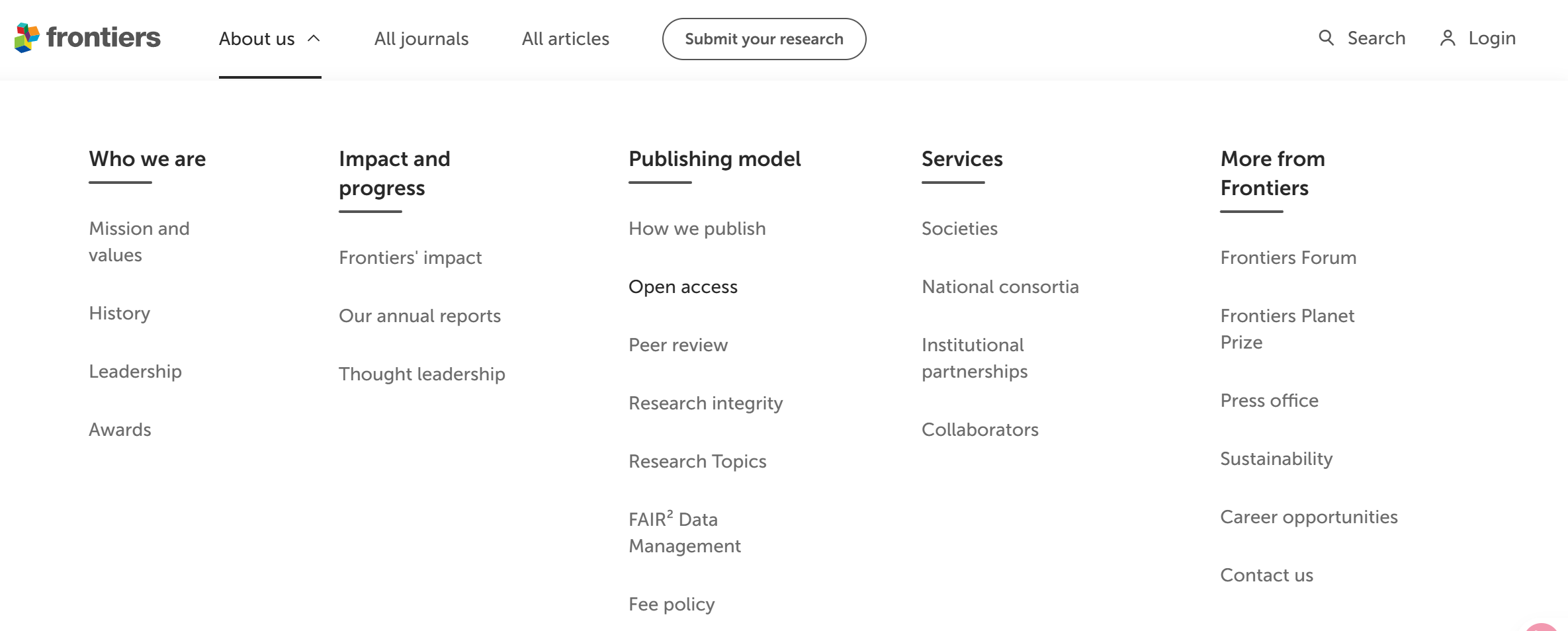


FIG6：

1


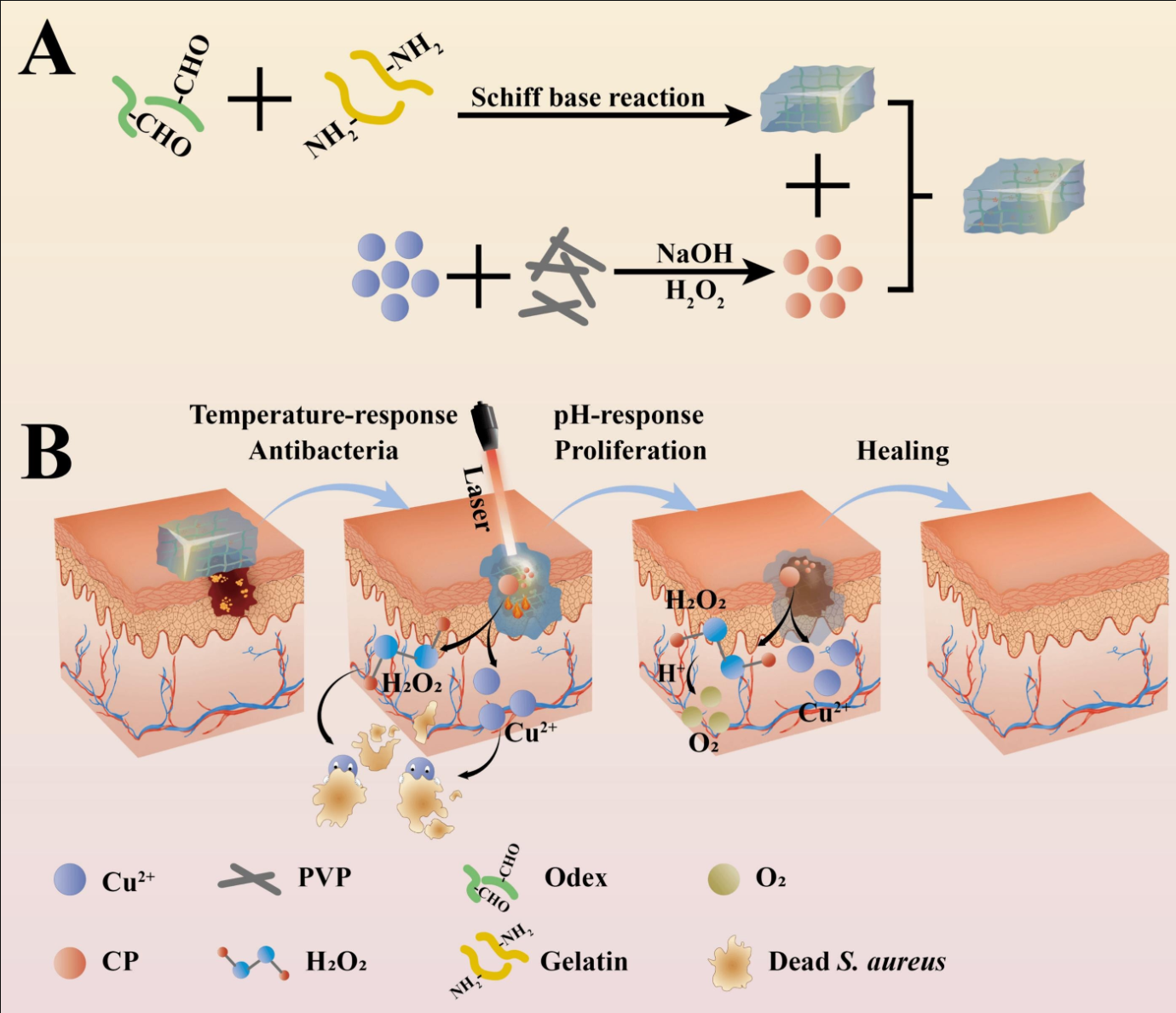


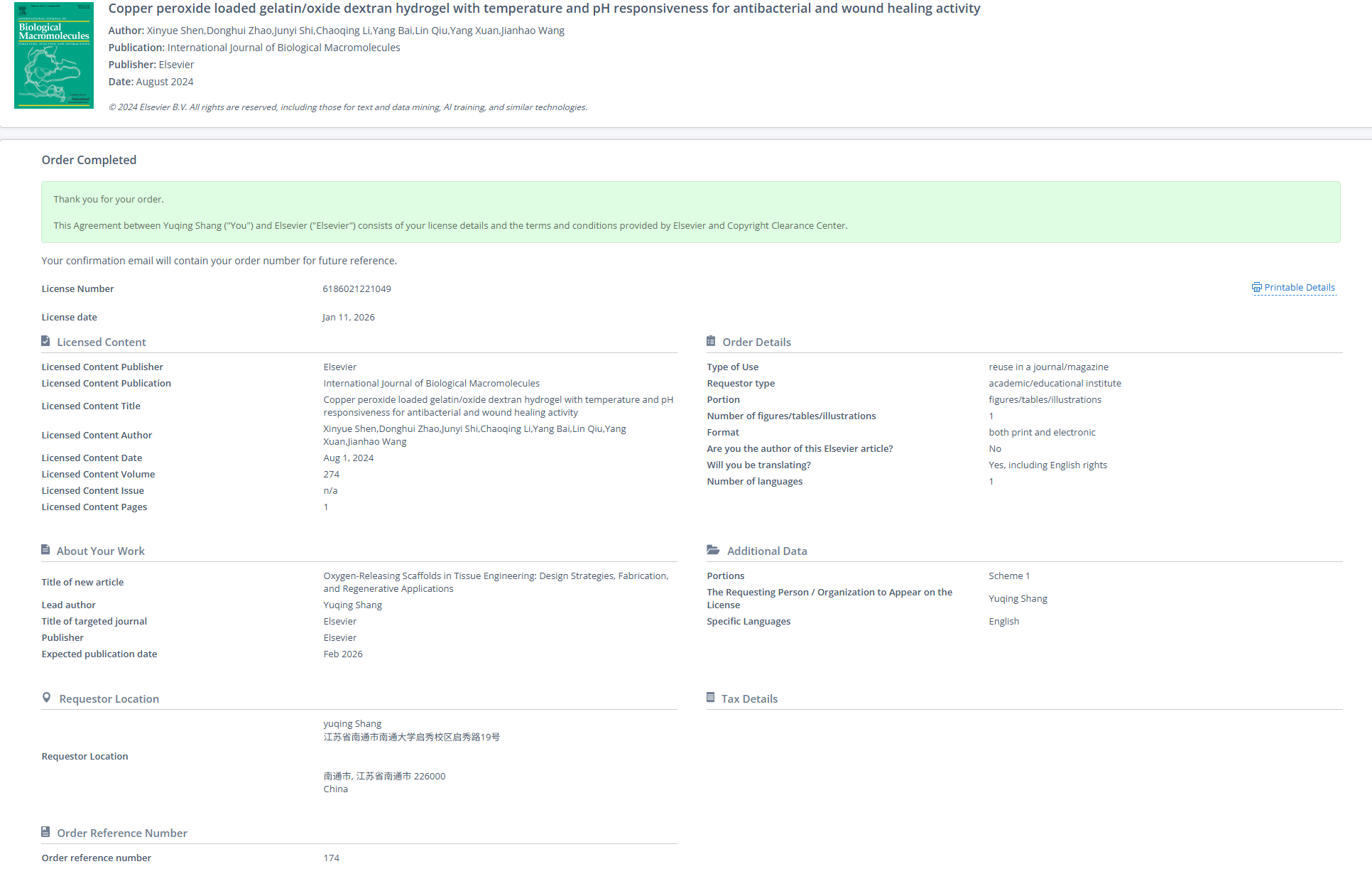


2.

2.
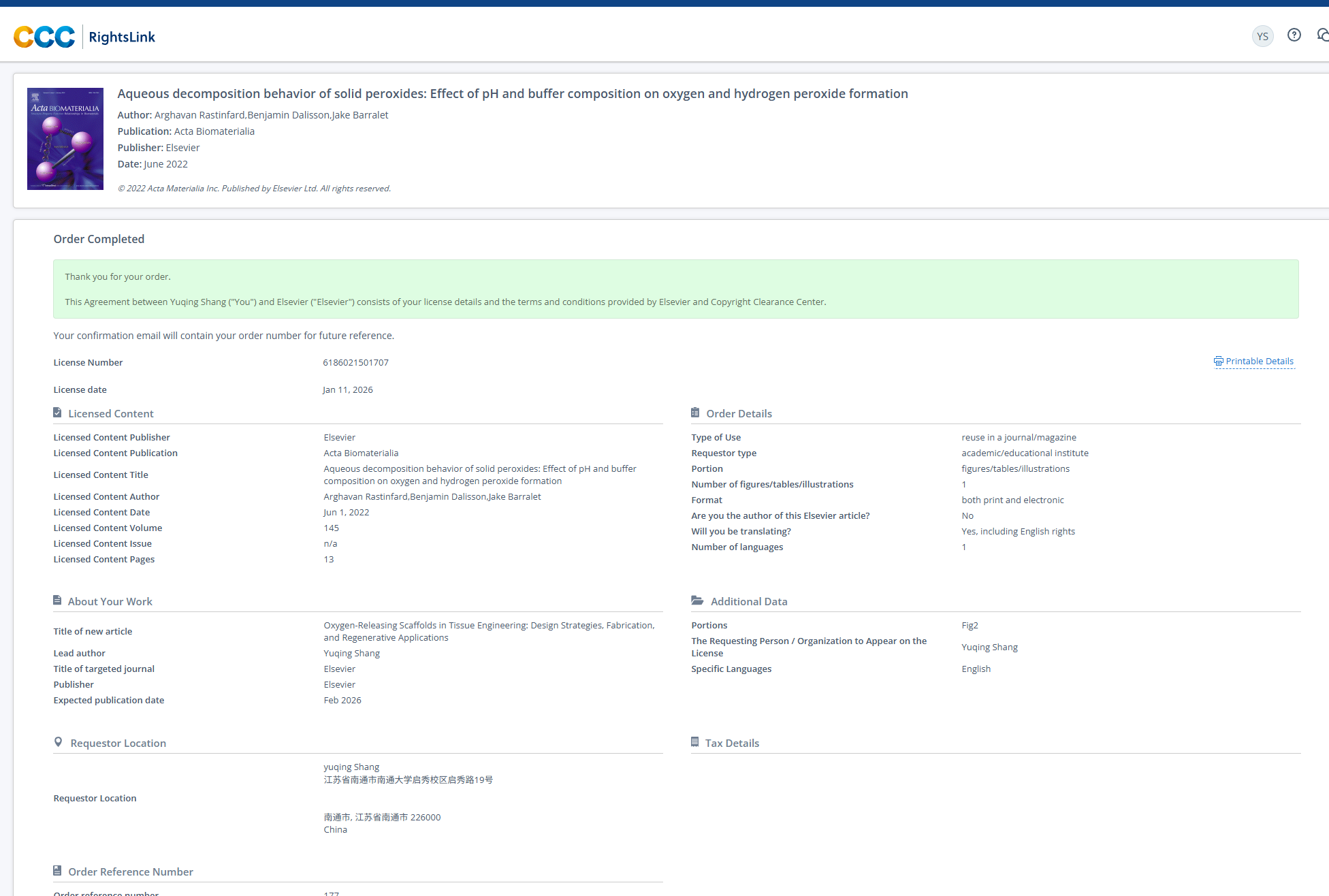


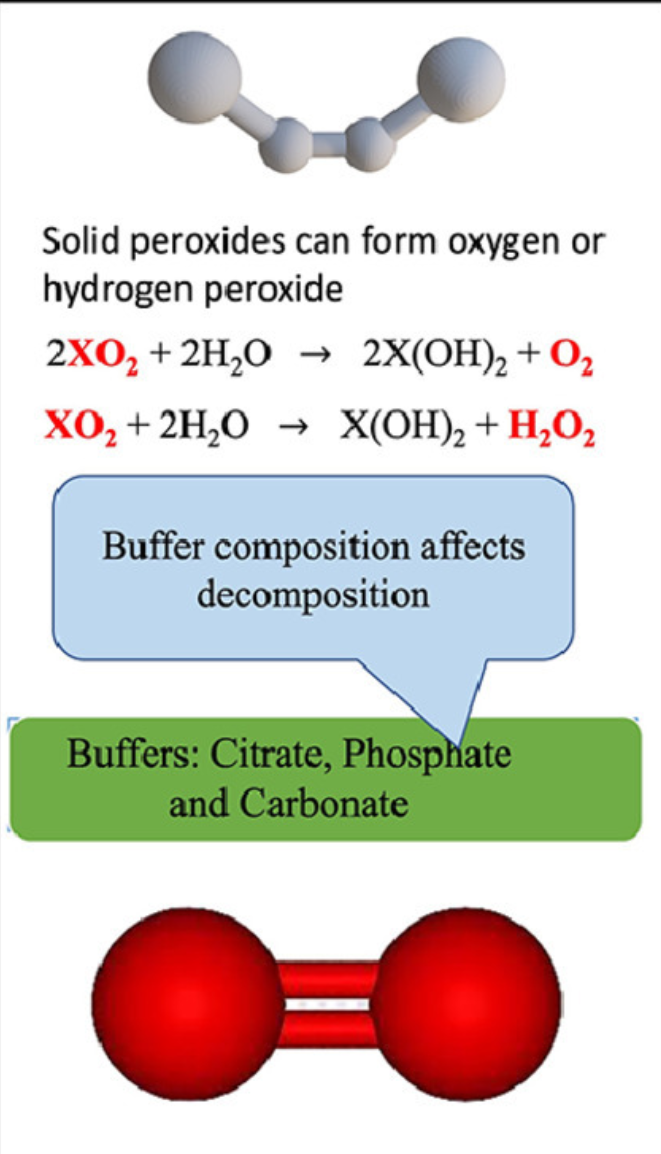

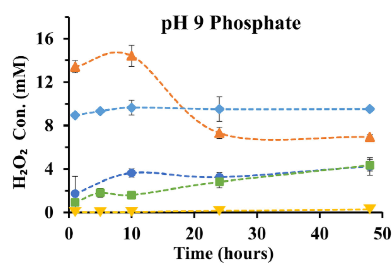

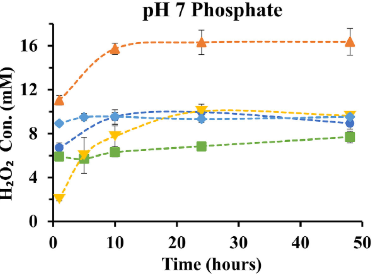

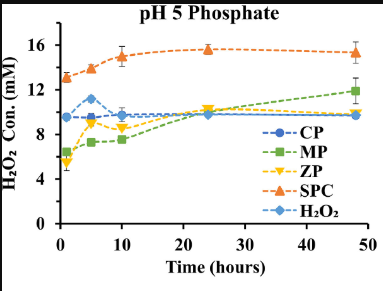


3.


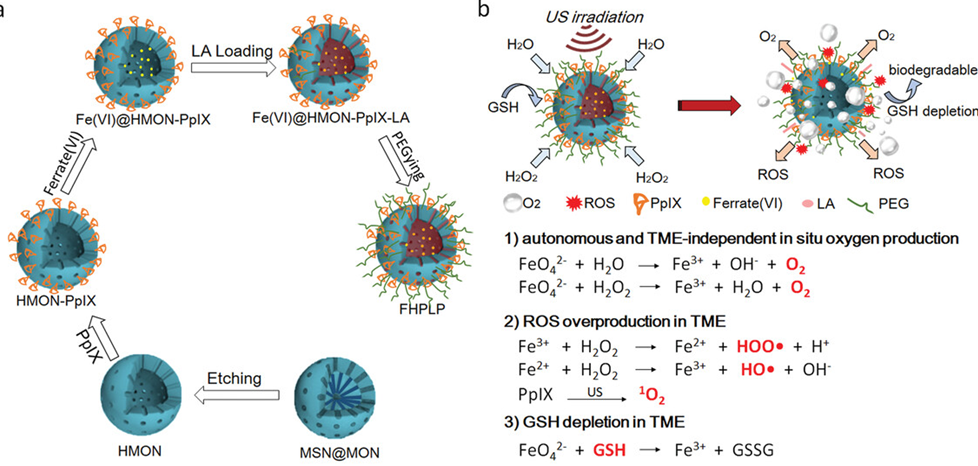


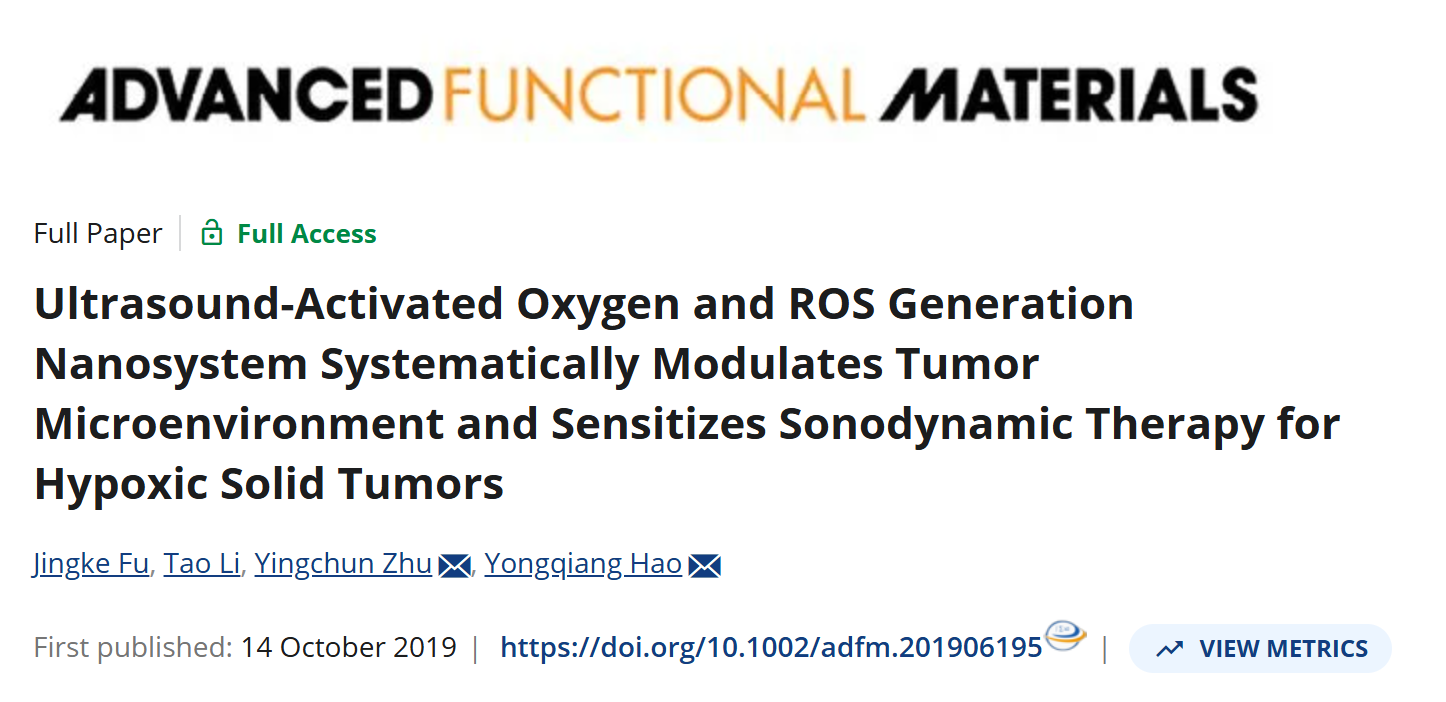


Fig7：

1.


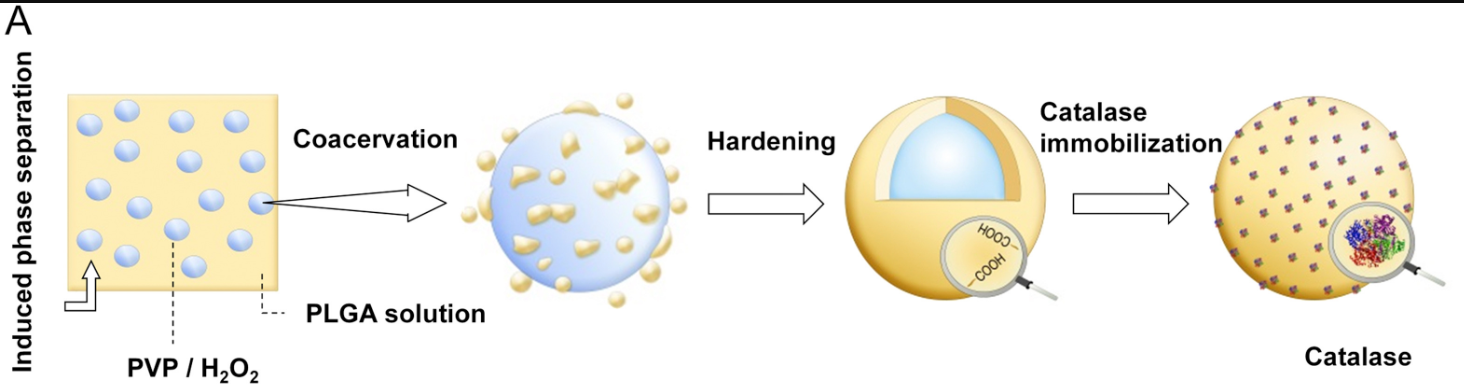


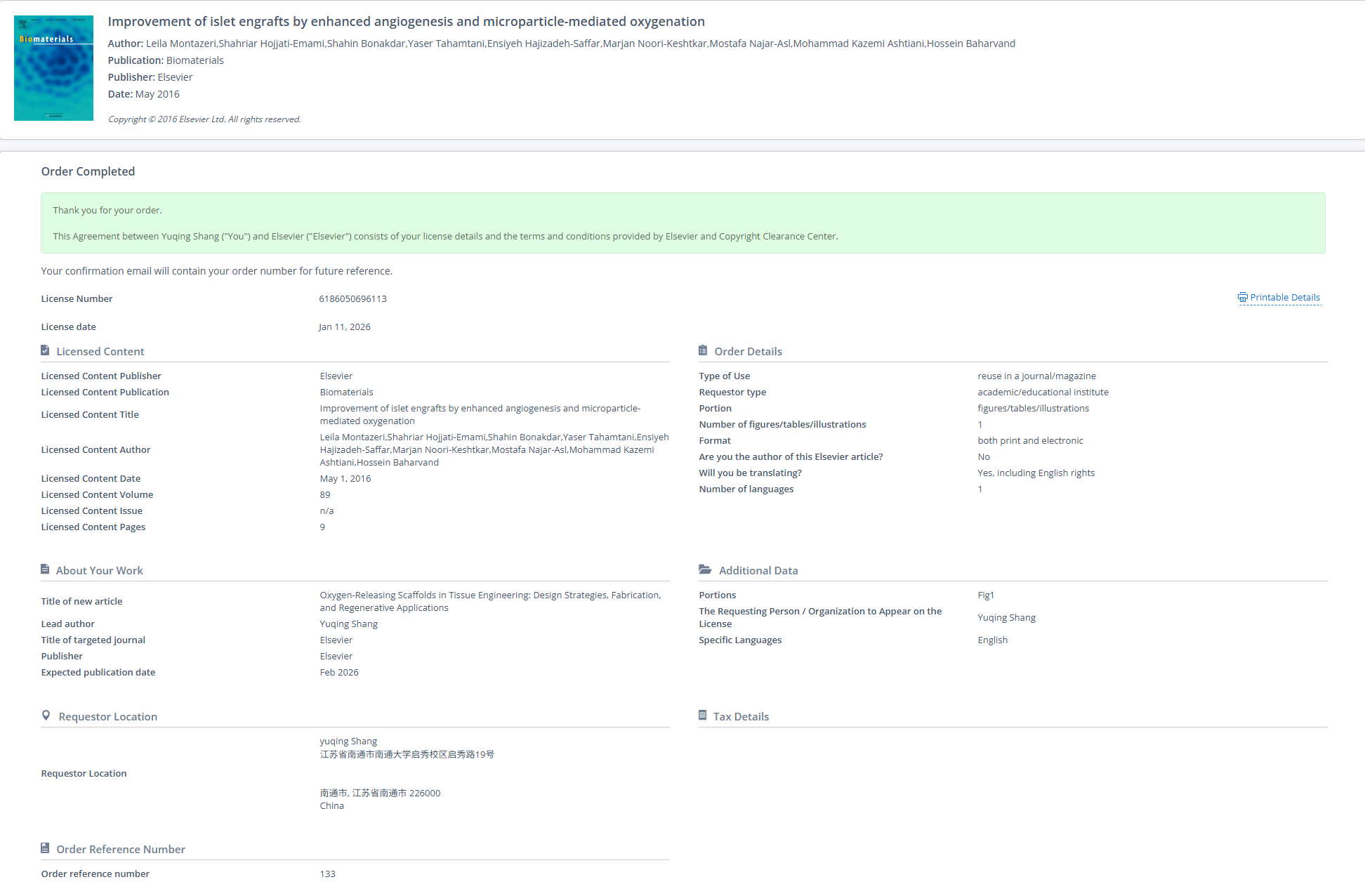


2.


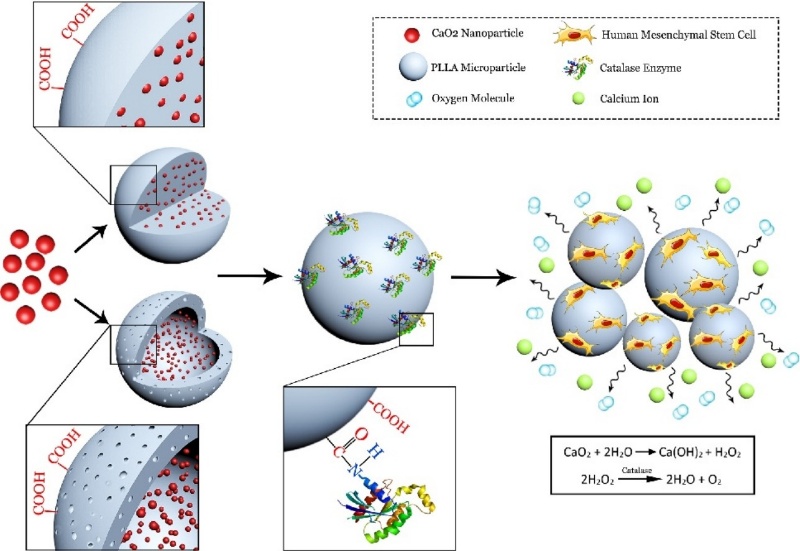


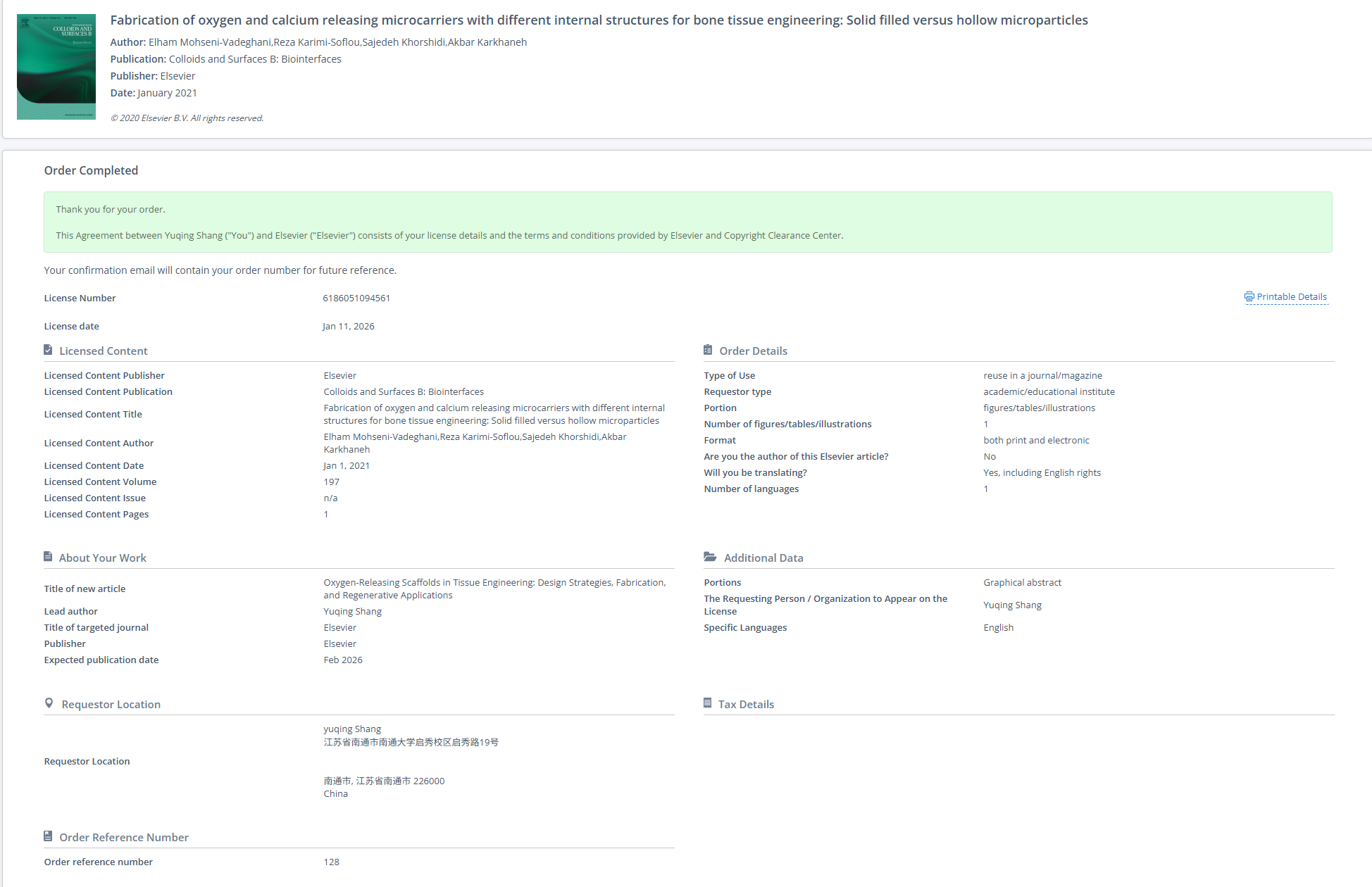


3.


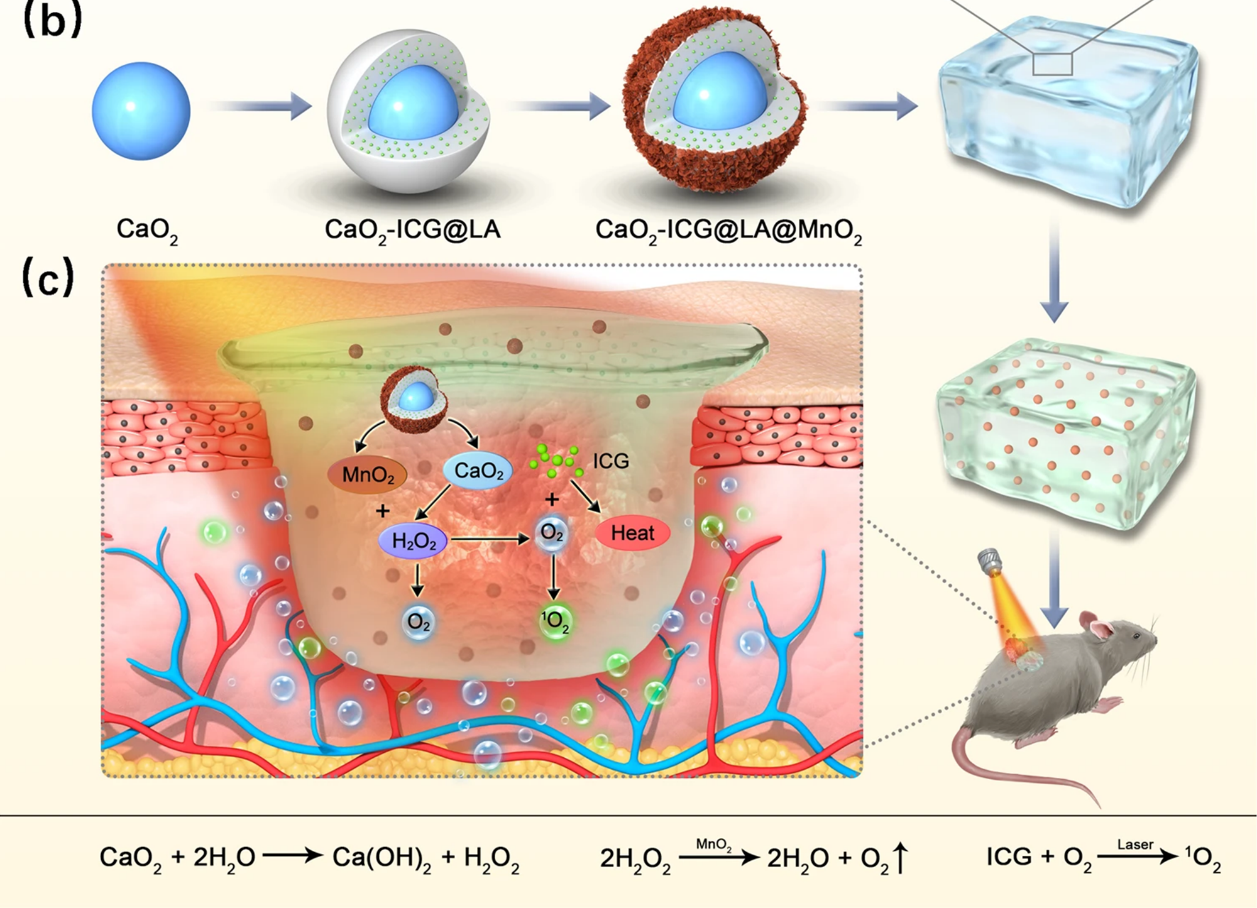


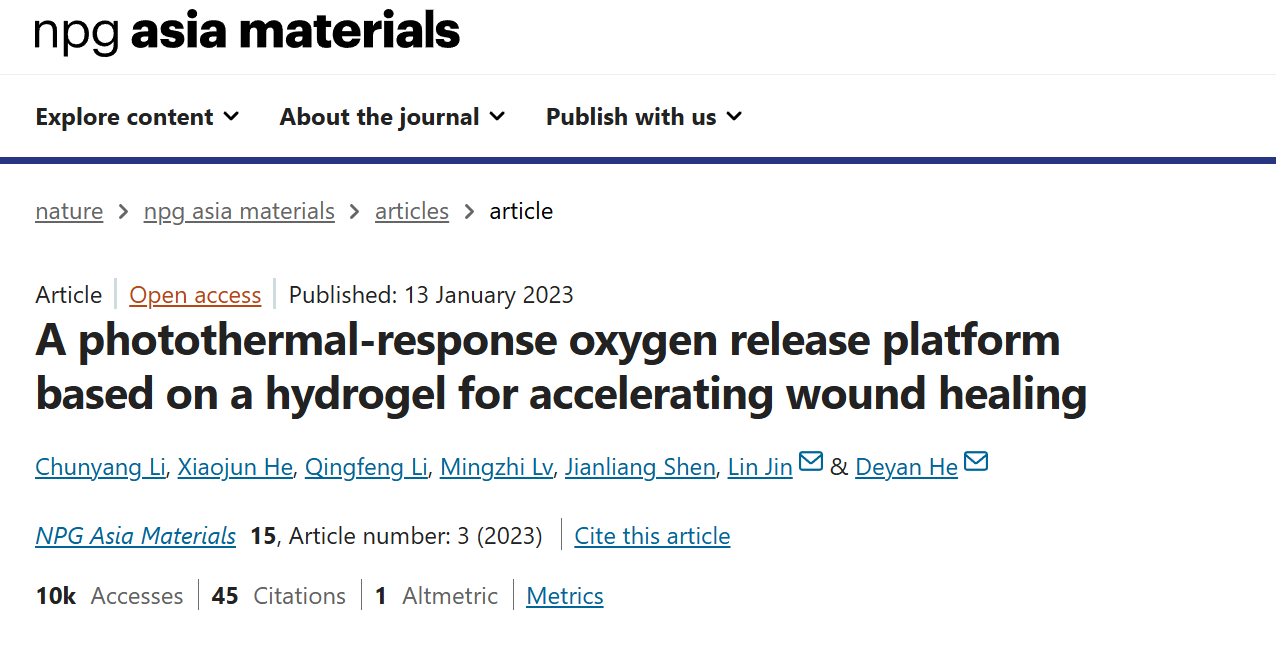


4.
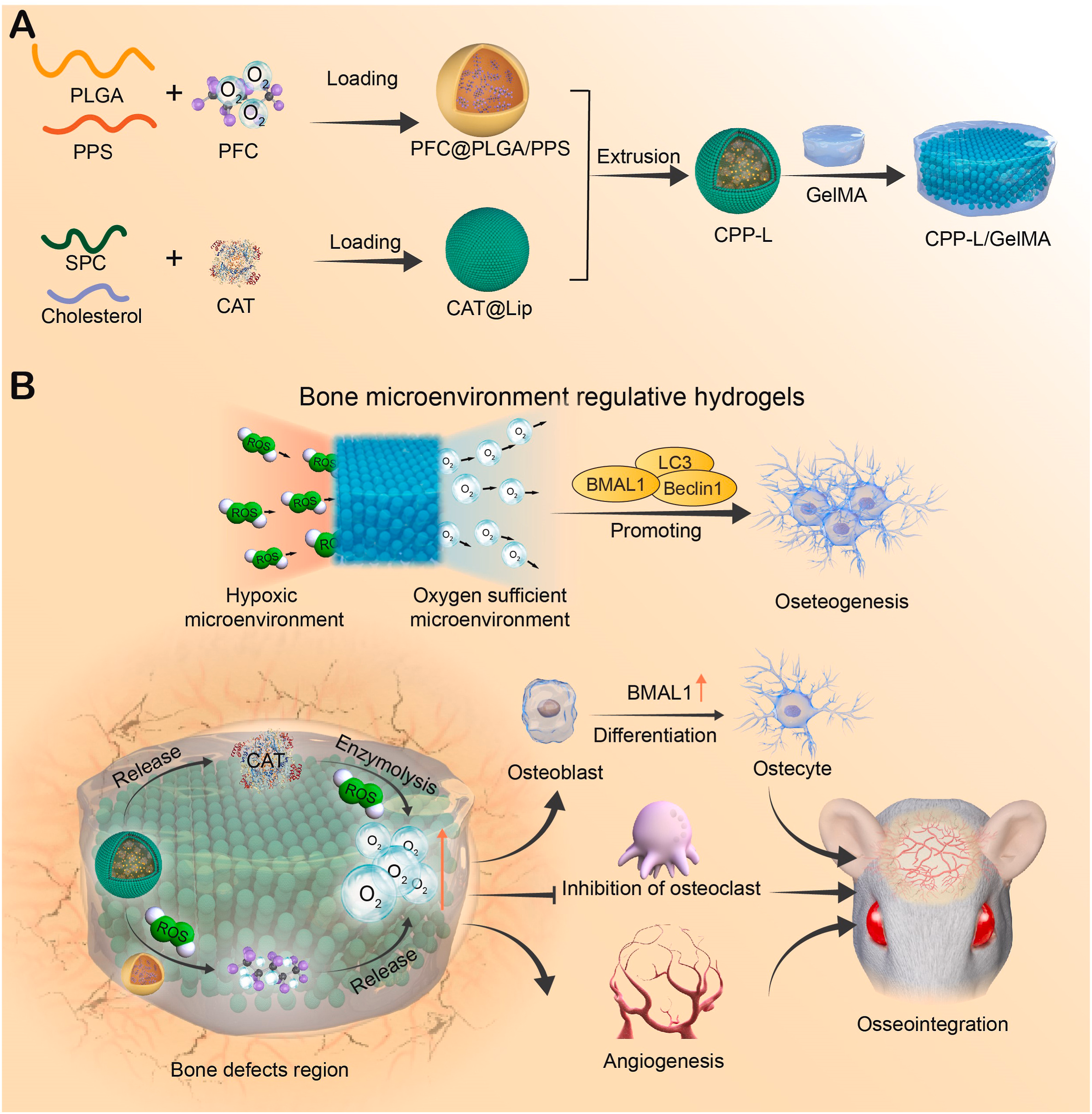


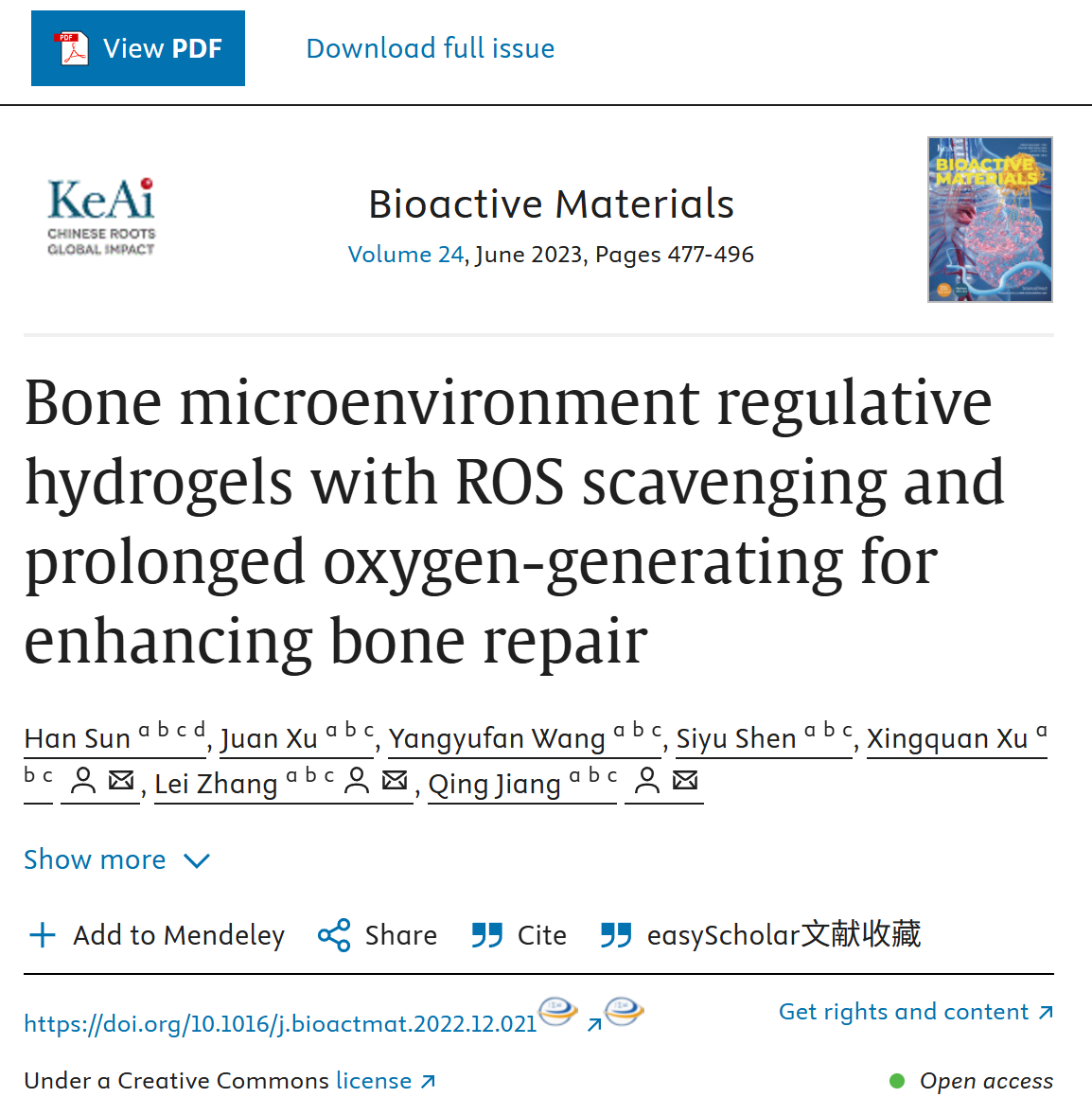

Supplement: rbag096_Supplementary_Data [file rbag096_supplementary_data.docx]
